# The N-Terminal Region of Fibrillin-1 Mediates a Bipartite Interaction with LTBP1

**DOI:** 10.1016/j.str.2017.06.003

**Published:** 2017-08-01

**Authors:** Ian B. Robertson, Hans F. Dias, Isabelle H. Osuch, Edward D. Lowe, Sacha A. Jensen, Christina Redfield, Penny A. Handford

**Affiliations:** 1 Department of Biochemistry, University of Oxford, South Parks Road, Oxford OX1 3QU, UK

**Keywords:** fibrillin, LTBP, TGF-β, extracellular matrix, NMR, SAXS, solution structure

## Abstract

*Fibrillin-1* (*FBN1*) mutations associated with Marfan syndrome lead to an increase in transforming growth factor β (TGF-β) activation in connective tissues resulting in pathogenic changes including aortic dilatation and dissection. Since FBN1 binds latent TGF-β binding proteins (LTBPs), the major reservoir of TGF-β in the extracellular matrix (ECM), we investigated the structural basis for the FBN1/LTBP1 interaction. We present the structure of a four-domain FBN1 fragment, EGF2-EGF3-Hyb1-cbEGF1 (FBN1^E2cbEGF1^), which reveals a near-linear domain organization. Binding studies demonstrate a bipartite interaction between a C-terminal LTBP1 fragment and FBN1^E2cbEGF1^, which lies adjacent to the latency-associated propeptide (LAP)/TGF-β binding site of LTBP1. Modeling of the binding interface suggests that, rather than interacting along the longitudinal axis, LTBP1 anchors itself to FBN1 using two independent epitopes. As part of this mechanism, a flexible pivot adjacent to the FBN1/LTBP1 binding site allows LTBP1 to make contacts with different ECM networks while presumably facilitating a force-induced/traction-based TGF-β activation mechanism.

## Highlights

• The structure of the FBN1 N-terminal region shows a near-linear domain organization
• LTBP1 binds to FBN1 via a bipartite mode of interaction involving two discreet sites
• This allows LTBP1 to connect 10–12 nm FBN1 microfibrils to other ECM networks
• This may facilitate force-induced/traction-based activation of TGF-β via integrins

## Graphical Abstract

**Figure:**
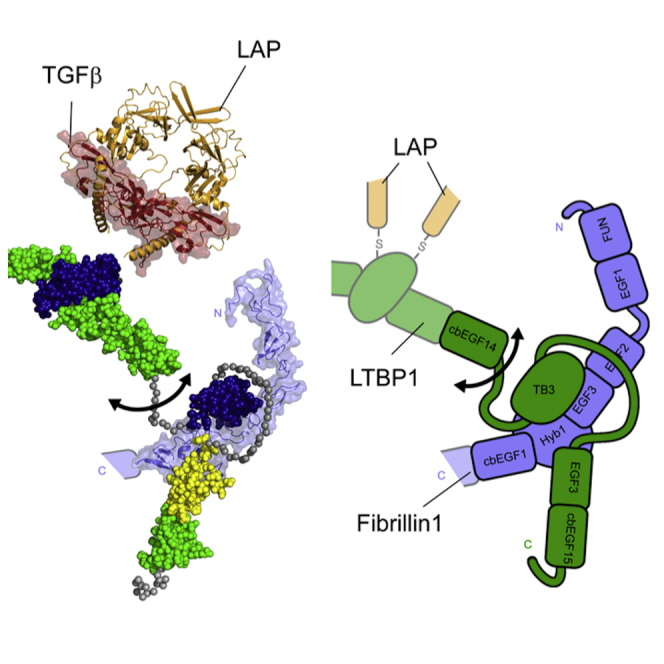

## Teaser

Improving our knowledge of TGF-β regulation by matrix biomechanics is vital for understanding the biology of this enigmatic growth factor. Robertson et al. present a bipartite model for the structure of the fibrillin-1-LTBP1 interaction that functions as a holdfast for TGF-β in the matrix.

## Introduction

**Figure 1:**
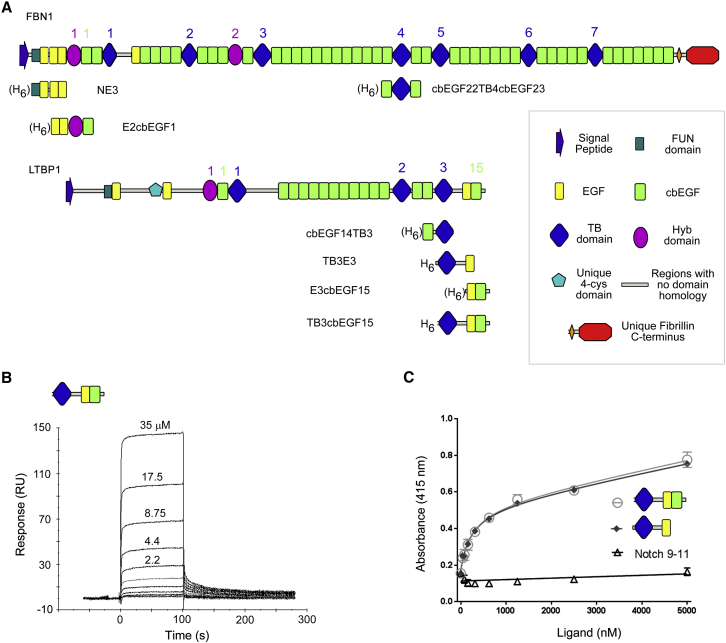
Overlapping Protein Constructs Used to Study the Interaction between FBN1 and LTBP1 (A) The FBN1 and LTBP1 constructs used in this study are shown in the context of the domain organization of the full-length proteins. “H_6_” in front of the constructs denotes that the protein is expressed with a hexa-His tag and the brackets indicate that it can be cleaved by factor Xa without degradation of the protein. Domain numbers for the TB and Hyb domains and for selected cbEGF domains are indicated and are color coded according to domain type. (B) Multi-cycle SPR data showing the concentration-dependent interaction of the LTBP1^TB3cbEGF15^ construct with FBN1^E2cbEGF1^ amine-coupled to the surface of the sensor chip. (C) Plate-binding assay showing the interaction of the His-tagged three-domain LTBP1^TB3cbEGF15^ (○) or two-domain LTBP1^TB3E3^ (♦) constructs with a four-domain FBN1^E2cbEGF1^ construct immobilized on the plate surface. No binding was seen with a control three-domain His-tagged Notch EGF9-11 construct (▵). The data presented are from a single plate; three repeats of each protein concentration were carried out to determine experimental error (SD). See also Figure S1.

The fibrillin/latent transforming growth factor β (TGF-β) binding protein (LTBP) family of extracellular matrix (ECM) proteins are calcium-binding glycoproteins whose domain organization is dominated by multiple tandem repeats of calcium-binding epidermal growth factor-like (cbEGF) and TGF-β binding protein-like (TB) domains (Figure 1A) (Robertson et al., 2011). Fibrillin (FBN) is an evolutionarily ancient protein which plays an important structural role in connective tissues through its higher-order association into 10–12 nm microfibrils (Jensen et al., 2012, Keene et al., 1991). The LTBPs are responsible for sequestering the small latent complex, comprising latency-associated propeptide (LAP) and TGF-β in the matrix (Saharinen and Keski-Oja, 2000). Recent research suggests that FBN also has an important regulatory role in development and homeostasis by transmitting diverse information about the extracellular environment to cells (Sengle and Sakai, 2015). Such signal transduction might occur through direct interactions between FBN and cell-surface integrin receptors (Pfaff et al., 1996, Sakamoto et al., 1996, Zeyer and Reinhardt, 2015) and/or through its direct or indirect sequestration of growth factors including various TGF-β superfamily members (Robertson and Rifkin, 2016).

Studies of genetic diseases and mouse models have demonstrated important physiological connections between FBN1 and TGF-β signaling *in vivo*. Marfan syndrome, caused by loss-of-function *FBN1* mutations, can result in aortic dilatation and dissection, as well as pathological changes to the skeleton and the eye (Doyle et al., 2012). Inhibition of TGF-β, either directly using neutralizing antibodies or indirectly using drugs such as Losartan, has demonstrated reversal of pathological changes in the aorta, lung, and skeleton in *FBN1* mouse models, suggesting that FBN1 levels in the ECM influence TGF-β activity (Cohn et al., 2007, Habashi et al., 2011, Lima et al., 2010, Neptune et al., 2003). Subsequent studies have shown that both integrin levels and changes in mechano-sensation by the angiotensin receptor can further modulate conditional *FBN* knockout mouse phenotypes indicating a complex cell/matrix interplay (Cook et al., 2014, Cook et al., 2015).

Cell biology studies have shown co-localization of FBN microfibrils with LTBPs in skin (Raghunath et al., 1998), bone (Dallas et al., 1995), and tissue culture (Dallas et al., 2000, Taipale et al., 1996). The LTBP isoforms LTBP1, 3, and 4 all covalently bind the small latent complex (comprising LAP and TGF-β) (Robertson et al., 2015, Saharinen and Keski-Oja, 2000). Subsequent biochemical studies have shown that there are interactions between eukaryotically expressed recombinant fragments of the N-terminal region of FBN1 and the C-terminal region of several of the LTBPs (Isogai et al., 2003, Massam-Wu et al., 2010, Ono et al., 2009). These studies identified the EGF2/EGF3 and Hybrid1 (Hyb1) domains of FBN1 and the TB3-EGF3-cbEGF15 region of LTBP1 as being important for binding. Collectively, mouse models, cell biology, and biochemical data place FBN, LTBP, and TGF-β at the center of a complex mechano-sensory network within connective tissue, the structural basis of which is unknown.

Here the structural basis for the interaction between FBN1 and LTBP1 has been investigated utilizing biophysical and biochemical techniques. The solution structure of a four-domain LTBP1-binding FBN1 fragment, EGF2-EGF3-Hyb1-cbEGF1, based on nuclear magnetic resonance (NMR) and small-angle X-ray scattering (SAXS) data, reveals a near-linear arrangement of domains, completing the structure of the N terminus of FBN1. Detailed dissection of the binding interface and subsequent modeling of the LTBP1/FBN1 complex indicates that LTBP1 binds FBN1 via its C-terminal TB3 and EGF3 domains in a bipartite interaction involving two different faces of the FBN1 molecule. This localized interaction ensures tight binding to FBN1 while allowing the N-terminal region of LTBP1 to engage with other ECM networks. This may facilitate regulated TGF-β activation by traction-based mechanisms involving integrins, and suggests that FBN1 deficiency precludes optimal localization of LTBP in the ECM for regulated TGF-β activation.

## Results and Discussion

### Binding Studies Identify a Specific Interaction between LTBP1 and FBN1

Previous data utilizing eukaryotically expressed fragments identified a specific interaction between the C-terminal region of LTBP1 and the N-terminal region of FBN1 (Isogai et al., 2003, Ono et al., 2009). Here, overlapping protein fragments derived from the N terminus of FBN1 and the C terminus of LTBP1 have been bacterially expressed and refolded *in vitro* (Figures 1A and S1), as described previously (Robertson et al., 2013a, Robertson et al., 2013b, Yadin et al., 2012), to probe their interaction at the molecular level and to determine a model of the interaction complex.

We observed a specific interaction between a three-domain C-terminal LTBP1^TB3cbEGF15^ construct and a four-domain FBN1^E2cbEGF1^ construct using both surface plasmon resonance (SPR) and a plate-based binding assay (Figures 1B and 1C). This confirms the interaction reported previously using eukaryotically expressed material (Isogai et al., 2003, Ono et al., 2009), and demonstrates that the core recognition elements are contained in the amino acid sequence of the proteins.

We compared the binding of the three-domain LTBP1^TB3cbEGF15^ and two-domain LTBP1^TB3E3^ constructs to the four-domain FBN1^E2cbEGF1^ construct using the plate-based assay (Figure 1C). The binding responses of the two LTBP1 fragments to FBN1 are the same, suggesting that the cbEGF15 domain of LTBP1 does not contribute to the interaction with FBN1.

**Figure 2:**
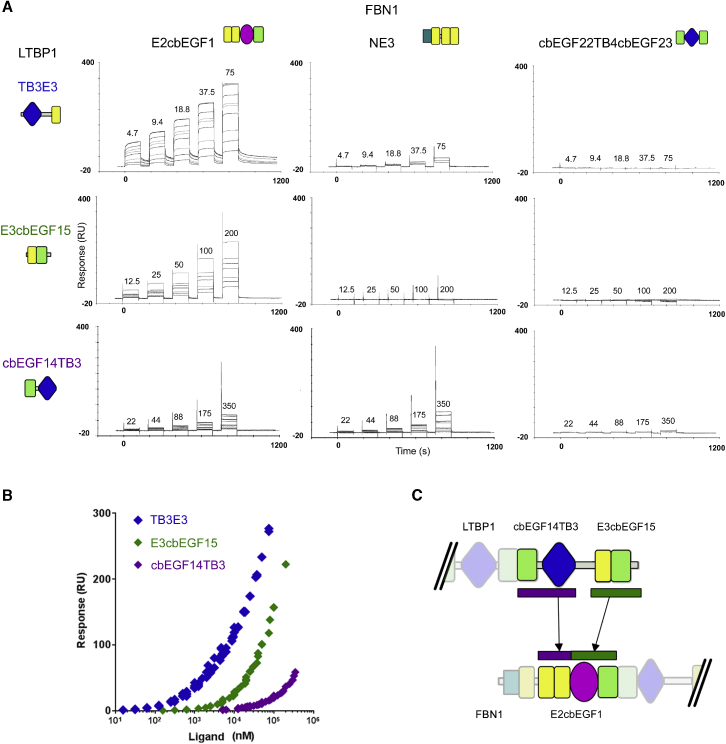
Interactions of FBN1 and LTBP1 Domain Constructs Measured by SPR (A) Single-cycle SPR data showing the interaction of three overlapping two-domain LTBP1 constructs (analytes) with three SPR flow cells coated with different FBN1 constructs. LTBP1 concentrations are shown in μM above the relevant part of the sensorgram for the highest concentration single-cycle experiment. The FBN1 construct containing the cbEGF22-TB4-cbEGF23 domains was used as a control. (B) Plot of SPR responses from three different LTBP1 constructs binding to FBN1^E2cbEGF1^; this demonstrates that for the interaction with FBN1^E2cbEGF1^ K_d_(TB3E3) < K_d_(E3cbEGF15) < K_d_(cbEGF14TB3). Concentrations are shown on a logarithmic scale to accommodate the weak binding of LTBP1^cbEGF14TB3^. (C) Schematic representation of the interaction model suggested by the SPR data. See also Figures S1 and S2.

To dissect the interaction further using SPR, analytes LTBP1^TB3E3^, LTBP1^E3cbEGF15^, and LTBP1^cbEGF14TB3^, each containing a pair of domains, were flowed over immobilized FBN1^E2cbEGF1^ (Figure 2A). The largest response was observed for LTBP1^TB3E3^ with moderate binding for LTBP1^E3cbEGF15^ and even weaker binding for LTBP1^cbEGF14TB3^ (Figure 2B). The high-affinity binding observed for LTBP1^TB3E3^ suggests that the TB3 and EGF3 domains, rather than the flanking cbEGF14 and cbEGF15 domains, are important for maximal binding. The observation of weak binding for both the LTBP1^cbEGF14TB3^ and LTBP1^E3cbEGF15^ constructs, which do not have any domains in common, suggests that more than one domain from LTBP1 interacts with FBN1. The relative binding strengths of the LTBP1 fragments also suggest that the EGF3 domain of LTBP1 makes a more significant contribution to the interaction with FBN1 than the TB3 domain of LTBP1.

The two-domain LTBP1 constructs were also flowed over a smaller immobilized FBN1 fragment, FBN1^NE3^, which shares the EGF2-EGF3 domains with FBN1^E2cbEGF1^ (Figures 1A and 2A). The substantially weaker interaction observed for LTBP1^TB3E3^ with FBN1^NE3^ than with FBN1^E2cbEGF1^ suggests that the Hyb1 and cbEGF1 domains of FBN1 contain the major LTBP1 binding site. The similar strength of interaction of LTBP1^cbEGF14TB3^ with FBN1^NE3^ and FBN1^E2cbEGF1^ indicates that the TB3 domain of LTBP1 must interact with the EGF2-EGF3 region of FBN1. The lack of an interaction between LTBP1^E3cbEGF15^ and FBN1^NE3^ suggests that the EGF3 domain of LTBP1 must interact with the Hyb1-cbEGF1 region of FBN1.

Dissociation constants (K_d_) of ∼100 ± 20 and ∼300 ± 100 μM for the interaction of FBN1^E2cbEGF1^ with LTBP1^E3cbEGF15^, and LTBP1^cbEGF14TB3^, respectively, can be estimated from the SPR data (Figure S2). In contrast, the binding of LTBP1^TB3E3^ to FBN1^E2cbEGF1^ gives a non-linear Scatchard plot (Figure S2); this is not surprising as the multi-site mode of interaction for this LTBP1 construct may give rise to complicated binding kinetics. Nevertheless, a K_d_ of ∼0.5–1 μM can be estimated from the SPR data at the lowest analyte concentrations (which are similar to concentrations used in previous studies [Massam-Wu et al., 2010, Ono et al., 2009]). Thus, the pair of interaction sites between FBN1 and LTBP1 results in a substantial enhancement in overall binding affinity. A cartoon summarizing our proposed binding model based on our domain dissection data is shown in Figure 2C.

### HSQC Titrations of FBN1 and LTBP1 Reveal Distinct Binding Sites

**Figure 3:**
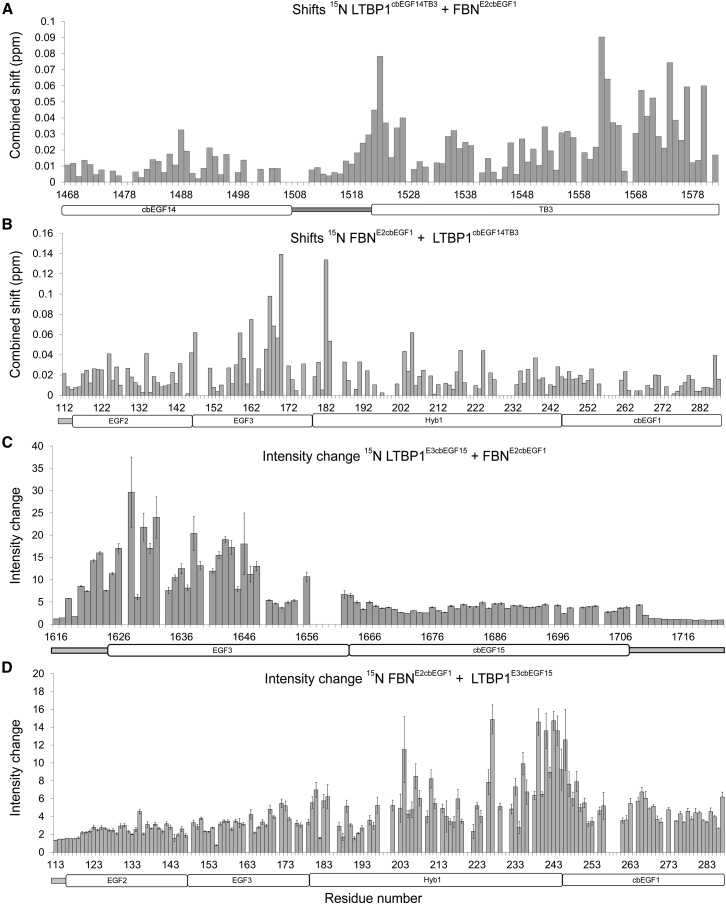
HSQC Titration Data Highlights Multiple Binding Sites in Both FBN1 and LTBP1 Titrations were carried out by sequential addition of lyophilized unlabeled protein to ^15^N-labeled protein samples and monitored using ^1^H-^15^N HSQC spectra. (A and B) Peaks were observed to shift in titrations of (A) ^15^N-LTBP1^cbEGF14TB3^ with FBN1^E2cbEGF1^ added, or of (B) ^15^N-FBN1^E2cbEGF1^ with LTBP1^cbEGF14TB3^ added. The combined ^1^H^N^ and ^15^N chemical shift change is plotted as a function of protein sequence. (C and D) Peak broadening was observed in titrations of (C) ^15^N-LTBP1^E3cbEGF15^ with FBN1^E2cbEGF1^ added or of (D) ^15^N-FBN1^E2cbEGF1^ with LTBP1^E3cbEGF15^ added. Peak intensity changes, measured as the ratio of peak intensity in the absence of ligand to that in the presence of ligand, are plotted as a function of protein sequence. Error bars are determined from the effect of background noise on peak height (SD). Gaps in the plots occur for residues with unassigned or very weak peaks in the HSQC or for prolines.

The SPR studies described above indicated that two sites are responsible for the interaction of the C-terminal region of LTBP1 and the N-terminal region of FBN1. To provide residue-specific information about each binding site, NMR titrations were carried out with a number of ^15^N-labeled protein constructs with assigned heteronuclear single quantum coherence (HSQC) spectra (Robertson et al., 2013b, Robertson et al., 2014). Titrations of the four-domain FBN1^E2cbEGF1^ construct with the two-domain LTBP1 construct, LTBP1^cbEGF14TB3^, exhibit specific chemical shift changes which identify residues that are involved in the interaction (Figures 3A and 3B); this fast exchange behavior on the NMR timescale is consistent with the relatively weak interaction between the two protein constructs seen by SPR. These titrations show that residues throughout the TB3 domain of LTBP1 interact with residues located in EGF3 and the N-terminal region of Hyb1 of FBN1 (Figures 3A and 3B). Titration of the four-domain FBN1^E2cbEGF1^ construct with the two-domain LTBP1 construct, LTBP1^E3cbEGF15^, shows specific broadening effects rather than chemical shift changes; this indicates intermediate/slow exchange behavior consistent with the stronger interaction between this pair of protein constructs seen by SPR (Figures 3C and 3D). These titrations show that residues located in the EGF3 domain of LTBP1, and some of the unstructured region that immediately precedes EGF3, interact with residues located in the Hyb1 and cbEGF1 domains of FBN1 (Figures 3C and 3D).

### SAXS and NMR Support an Extended Near-Linear Conformation for FBN1^E2cbEGF1^ in Solution

**Figure 4:**
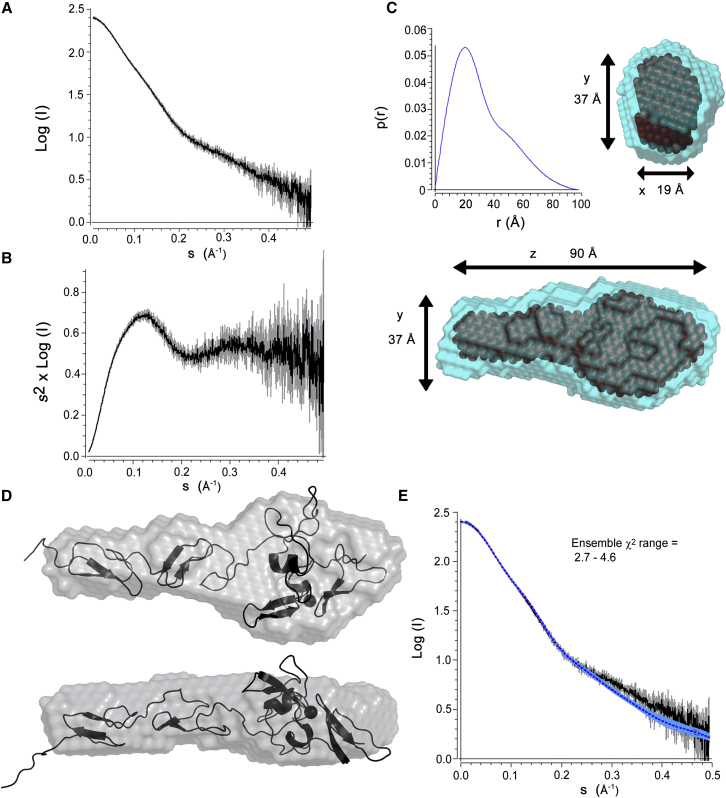
SAXS Data for FBN1^E2cbEGF1^ Reveals a Linear Shape (A) Scaled, merged, and averaged X-ray scattering curves collected with purified FBN1^E2cbEGF1^ at concentrations of 11.55, 5.78, 2.89, and 1.44 mg/mL. Analysis of these data confirm that the protein behaves as a monomer in solution. (B) Kratky plot of scaled, merged, and averaged SAXS data showing a peak falling to a plateau; this behavior is characteristic of a folded and relatively rigid protein. (C) P(r) distribution and *ab initio* modeling of particle shape using DAMMIF. The blue transparent surface represents the shape produced from averaging the 20 independently generated *ab initio* structures with DAMAVER, and the darker spheres within this envelope represent the core shared particle shape calculated by the DAMFILT algorithm. Fitting this DAMFILT particle shape to the scattering data gave a χ^2^ value of 0.7240. (D) Comparison of a selected model (black cartoon) from the NMR structural ensemble with the envelope produced by DAMFILT *ab initio* modeling (gray surface). (E) Fitting of the 20 structures in the NMR ensemble to the SAXS data. The SAXS data and error bars are shown in black and gray, respectively. The fits of the NMR structures are shown in blue with a dark blue dashed line showing the fit of model 1 of the NMR ensemble. The error bars in (A), (B), and (E) are derived from the SCATTER software package using the data collected at four proteins concentrations.

To investigate the overall shape of the FBN1^E2cbEGF1^ fragment and to assess its flexibility in solution, SAXS measurements were collected. *Ab initio* structural modeling and analysis of P(r) distributions support the idea of the EGF2-EGF3-Hyb1-cbEGF1 fragment adopting a linear conformation in solution. The Kratky plot is also consistent with a folded protein and does not show signs of significant flexibility (Figures 4A–4C).

**Figure 5:**
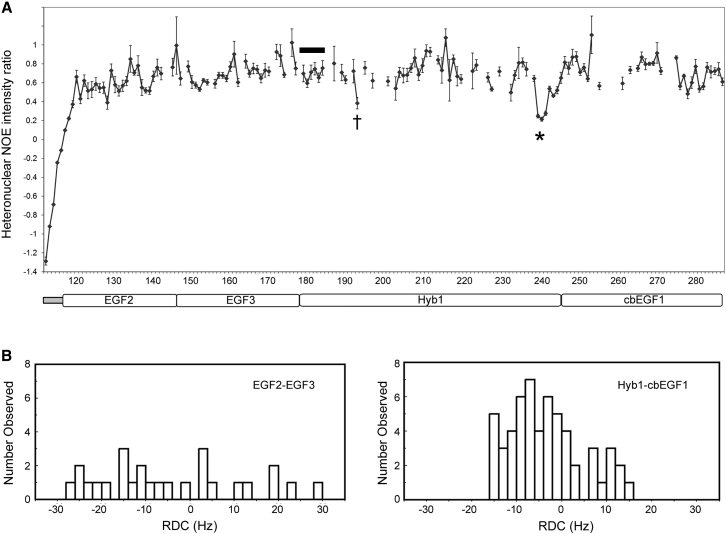
Heteronuclear NOE and RDC Data for FBN1^E2cbEGF1^ (A) {^1^H}-^15^N heteronuclear NOE ratios are plotted as a function of sequence for FBN1^E2cbEGF1^. Low heteronuclear NOE ratios (<0.5) at the N terminus of EGF2 and for two loops in Hyb1 (†, ^∗^) indicate significant flexibility on a picosecond to nanocsecond timescale in these regions. The residues linking EGF3 to Hyb1, indicated by a thick black line, show NOE ratios characteristic of a rigid protein backbone demonstrating that the EGF3-Hyb1 linker is not flexible on a fast timescale. Error bars for the NOE ratio were estimated from 500 Monte Carlo simulations using baseline noise as a measure of the error in the peak heights. (B) Distribution of RDC values measured in 4% bicelles for the EGF2-EGF3 and Hyb1-cbEGF1 domain pairs of FBN1^E2cbEGF1^. The different distribution of RDC values for the two pairs suggests that there may be some slower timescale flexibility at the EGF3-Hyb1 interface. Similar differences in RDC distribution are observed for RDCs measured in 2.2% C12E6/n-hexanol (data not shown).

In our previous studies of the C-terminal domains of LTBP1, {^1^H}-^15^N heteronuclear nuclear Overhauser effect (NOE) data identified the presence of flexible linkers between the cbEGF14, TB3, and EGF3 domains (Robertson et al., 2014). Heteronuclear NOE data collected here for FBN1^E2cbEGF1^ show fast timescale flexibility at the N terminus of EGF2 and for loop regions in Hyb1 (Figure 5A). However, no evidence of fast timescale dynamics is observed for the residues linking EGF3 to Hyb1. Furthermore inter-domain ^1^H-^1^H NOEs are observed between residues at the C terminus of EGF3 and the N terminus of Hyb1, consistent with the presence of a specific interface (data not shown).

Residual dipolar couplings (RDCs) were collected for FBN1^E2cbEGF1^. RDCs are a useful NMR parameter for assessing the relative orientations of protein domains in solution (Braddock et al., 2001, Weisshuhn et al., 2016) and were used previously to confirm the linear orientation of LTBP1 EGF3-cbEGF15 (Robertson et al., 2014). The distribution of RDCs observed for the EGF2-EGF3 and Hyb1-cbEGF1 pairs are not the same, indicating that some slower timescale movement of the domain pairs relative to each other may exist in solution (Figure 5B). Overall, these NMR data suggest that EGF3 and Hyb1 form an interface, but this may not be quite as rigid in solution as the EGF2-EGF3 and Hyb1-cbEGF1 interfaces.

### Solution Structure of FBN1^E2cbEGF1^

The solution structure of the N-terminal region of FBN1, comprising the unique N-terminal domain (FUN) and EGF1-3 (Figure 1A), has been determined previously (Yadin et al., 2013). This showed a rigid orientation of the FUN-EGF1 pair, a flexible linker between EGF1 and EGF2, and a rigid orientation of the EGF2-EGF3 pair. Structural information for the N-terminal region of FBN1 beyond EGF3, which contains the main LTBP1 binding site, has not been available. Attempts to crystallize the four-domain FBN1^E2cbEGF1^ construct were unsuccessful.

**Figure 6:**
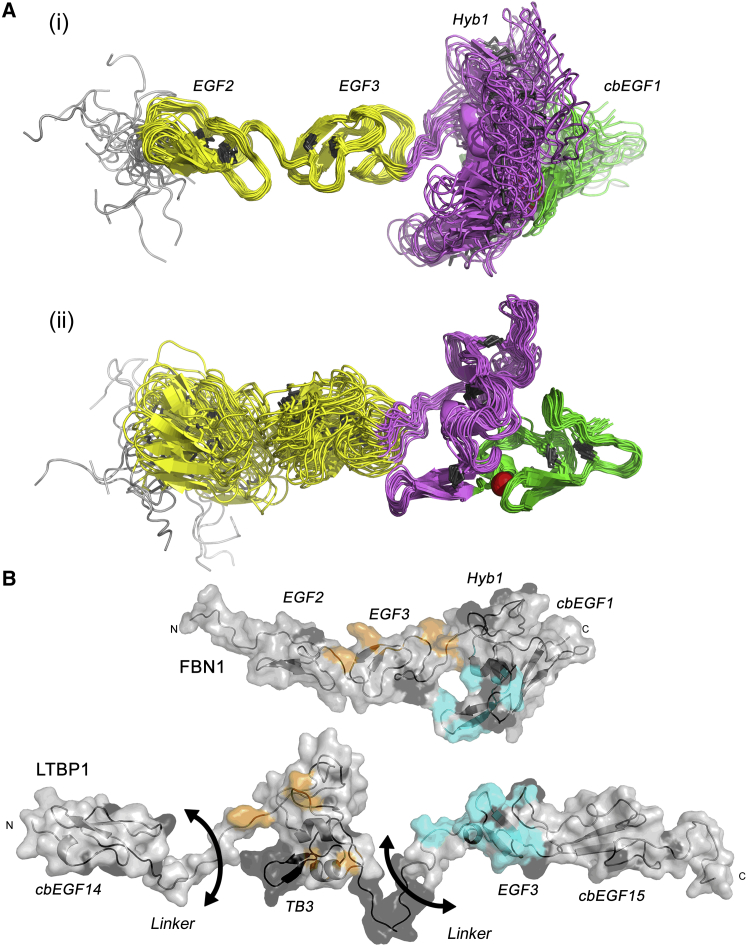
Solution Structural Ensemble of FBN1^E2cbEGF1^ and Interaction Sites Mapped onto the FBN1 and LTBP1 Structures (A) Cartoon representation of the 20-structure ensemble of FBN1^E2cbEGF1^ with structures aligned to (i) the EGF2-EGF3 domain pair or (ii) the Hyb1-cbEGF1 domain pair. The EGF, Hyb, and cbEGF domains are colored in yellow, purple, and green, respectively, the calcium ion is shown as a red sphere, and disulfide bonds are shown as dark gray lines. (B) Residues identified by peak shifts or peak broadening in HSQC spectra from FBN1 and LTBP1 titrations are highlighted on the solution structure of the EGF2-EGF3-Hyb1-cbEGF1 region of FBN1 and on a validated homology model of the cbEGF14-TB3-EGF3-cbEGF15 region of LTBP1 (Robertson et al., 2011, Robertson et al., 2014). Residues in FBN1 and LTBP1 that experience combined peak shifts of more than 0.06 and 0.05 ppm, respectively, in titrations of FBN1^E2cbEGF1^ with LTBP1^cb14TB3^ are shown in orange. Residues in FBN1 and LTBP1 that experience at least a 7- or 10-fold loss of intensity, respectively, in titrations of FBN1^E2cbEGF1^ with LTBP1^E3cbEGF15^ are shown in cyan. In both proteins, residues that have shifts or intensity changes below the relevant thresholds are shown in light gray, whereas residues that were not assigned are shown in dark gray. See also Figures S3 and S4.

Here, the solution structure of FBN1^E2cbEGF1^ was determined by a simulated annealing approach using distance restraints derived from 2D and 3D nuclear Overhauser effect spectroscopy (NOESY) spectra, torsion angle restraints derived from TALOS+ analysis of chemical shifts (Shen et al., 2009), hydrogen bond restraints derived from hydrogen-deuterium exchange experiments, RDC restraints collected in two alignment media, and a linearity restraint consistent with the SAXS data (Figure 6A; Table 1). Each domain within FBN1^E2cbEGF1^ adopts its expected characteristic fold. The EGF2-EGF3 and Hyb1-cbEGF1 pairs each contain a rigid interface defined by extensive NOEs and RDCs, and superposition of these domain pairs results in relatively low root-mean-square deviation values (Table 1). The structure and inter-domain interactions of the EGF2-EGF3 domain pair are consistent with the previously published solution structure of FBN1^NE3^ (Yadin et al., 2013). However, the C-terminal loop of EGF3 is better defined in the FBN1^E2cbEGF1^ structure presented here since residues in this region are involved in specific packing interactions with the Hyb1 domain that are absent in FBN1^NE3^ (Robertson et al., 2013b). The interface formed between Hyb1 and cbEGF1 is characterized by a number of specific side-chain interactions (Figure S3). The side chains of F235 (Hyb1) and V266 (cbEGF1) form a packing interaction that is likely to stabilize the Ca^2+^-binding site, as similar packing interactions are observed in other high-affinity Hyb-cbEGF and TB-cbEGF pairs (Jensen et al., 2009).

**Table 1:** NMR Structure Calculation Statistics

	FBN1^E2cbEGF1^
NOE-derived distance restraints	
Total	3,222
Total unambiguous	3,072
Intra-residue	1,261
Inter-residue	1,811
Sequential (|i − j| = 1)	726
Short-range (|i − j| < 5)	351
Long-range (|i − j|≥ 5)	734
Ambiguous	150
Hydrogen bond restraints	78
Dihedral angle restraints	161
RDCs	169
^1^D_NH_ 2.2% C12E6/n-hexanol	85
^1^D_NH_ 4% bicelles	84
Calcium-binding restraints	7
Restraint violations (average of full ensemble)	
Distance restraint violations >0.5 Å	4.35
Dihedral angle violations >5°	2.85
RMSD from experimental restraints	
Distance restraints (Å)	0.057 ± 0.003
Dihedral angle restraints (°)	1.323 ± 0.402
RDC restraints (Hz)	2.155 ± 0.141
RMSD from idealized geometry	
Bonds (Å)	0.007 ± 0.000
Angles (°)	0.822 ± 0.021
Impropers (°)	2.50 ± 0.12
Ramachandran plot (%)	
Residues in most favored regions	80 ± 2
Residues in additional allowed regions	15 ± 2
Outlier residues	5 ± 1
Coordinate precision (RMSD; Å)	
Backbone	
EGF2-EGF3 residues 119–178	0.80
Hyb1-cbEGF1 residues 204–287	0.71
Heavy atom	
EGF2-EGF3 residues 119–178	1.17
Hyb1-cbEGF1 residues 204–287	1.15

NOE, nuclear Overhauser effect; RDCs, Residual dipolar couplings; RMSD, root-mean-square deviation.

The EGF3-Hyb1 interface is less well defined in the solution structures (Figure S4). NOEs are observed between residues 170–173 in EGF3 and residues 179–183 in Hyb1. The latter residues are restricted to the very N terminus of Hyb1, which is well defined with respect to the EGF2-EGF3 domain pair. The ambiguity in domain orientation hinges around residues 183 and 184, with only limited NOEs between these “linking” Hyb1 residues preceding 184 and residues 203, 204, 205, and 217 in the rest of the Hyb1 domain.

The ensemble of 20 solution structures has been used to back calculate the SAXS data. All structures give good agreement with the data with χ^2^ values ranging from 2.7 to 4.6. The overall shape of the solution structure fits well with the envelope determined from the SAXS data, supporting an extended arrangement of the domains (Figures 4D and 4E).

### Structural Modeling and Mutagenesis of the LTBP1-FBN1 Complex

The NMR titration data (Figure 3) can be mapped onto the structure of FBN1^E2cbEGF1^ and our previous model of the cbEGF14-TB3-EGF3-cbEGF15 domains of LTBP1 (Robertson et al., 2014). Two distinct binding sites are apparent that suggest two different faces of the FBN1 N terminus are involved in interacting with the two separate FBN1-binding domains in the LTBP1 C terminus (Figure 6B). Since attempts to crystallize the complex were unsuccessful, HADDOCK, a data-driven protein-protein docking approach, was used to generate models of the complex (van Zundert et al., 2016).

**Figure 7:**
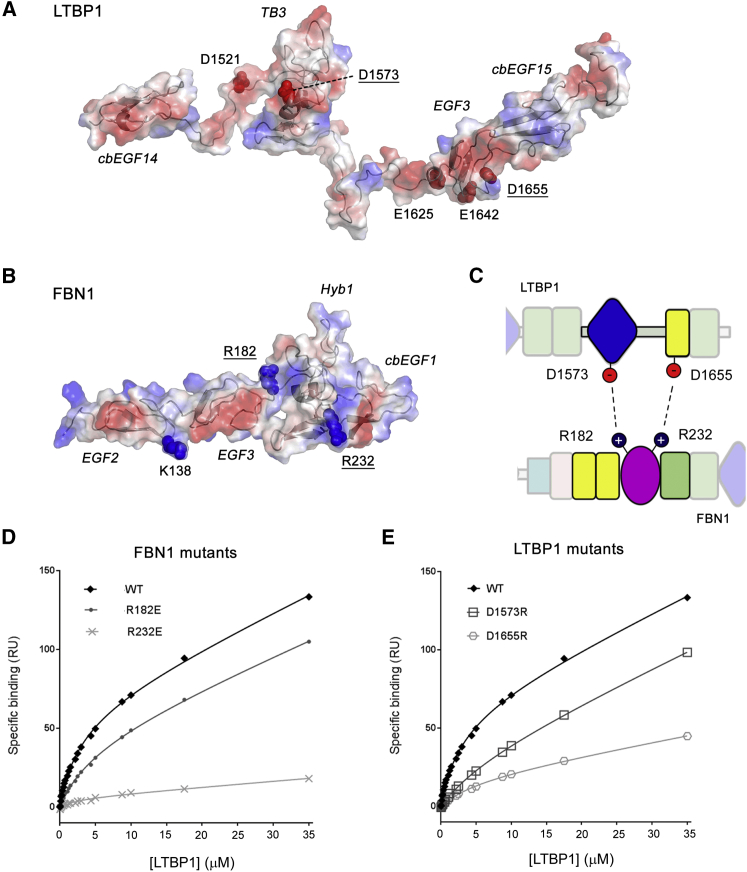
Effects of Salt Bridge Substitutions on FBN1-LTBP1 Interactions (A) Electrostatic surface representation for LTBP1 (cbEGF14-TB3-EGF3-cbEGF15); negatively charged surface is shown in red and positively charged surface in blue. Acidic residues identified in LTBP1 as potential salt bridge partners by preliminary HADDOCK modeling are shown as dark red spheres. D1521 and D1573 are involved in salt bridges in HADDOCK models of the FBN1-LTBP1^TB3^ complex, while E1625, E1642, and D1655 are involved in salt bridges in models of the FBN1-LTBP1^E3cbEGF15^ complex. Substitution of underlined residues showed reductions in binding. (B) Electrostatic surface representation for FBN1 (EGF2-EGF3-Hyb1-cbEGF1). Basic residues identified in FBN1 as potential salt bridge partners in initial HADDOCK modeling are shown as dark blue spheres. K138 and R182 are involved in salt bridges in HADDOCK models of the FBN1-LTBP1^TB3^ complex, while R232 is involved in salt bridges in models of the FBN1-LTBP1^E3cbEGF15^ complex. (C) Simplified model representing charge-charge interactions between the Hyb1 domain of FBN1 and the TB3 and EGF3 domains of LTBP1 with domains colored as in Figure 1. (D) Plot of SPR responses from binding of WT LTBP1^TB3E3^ analyte to three different FBN1^E2cbEGF1^ constructs immobilized to different flow cells of the same chip. (E) Plot of SPR responses from three different LTBP1^TB3E3^ constructs binding immobilized WT FBN1^E2cbEGF1^. See also Figures S1 and S5; Table S2.

Initial calculations, based only on the NMR titration data, identified a number of possible binding orientations. One common feature in the models was the presence of salt bridges that were frequently seen between basic residues in FBN1 and acidic residues in LTBP1 (Figures 7A and 7B); different pairs of these residues were involved in the formation of inter-molecular salt bridges in different models. K138 and R182 of FBN1 and D1521 and D1573 in LTBP1 are involved in salt bridges in HADDOCK models of the FBN1/LTBP1^TB3^ complex, while R232 of FBN1 and E1625, E1642 and D1655 of LTBP1 are involved in salt bridges in models of the FBN1/LTBP1^E3cbEGF15^ complex. To test the importance of these potential salt bridges, a number of charge-reversal substitutions were introduced in FBN1 and LTBP1. Variant proteins were produced (Figure S1) and their interactions assessed using SPR and plate-based assays.

Variants of FBN1 containing the R182E and R232E substitutions, and variants of LTBP1 containing the D1573R and D1655R substitutions, all showed reduced binding to wild-type (WT) LTBP1 and FBN1, respectively (Figures 7D, 7E, and S5), while substitutions K138D, D1521K, E1625R, and E1642R did not (data not shown). D1655R and R232E had the larger effect on binding consistent with a role for these residues in the stronger LTBP1-EGF3/FBN1-Hyb1 interaction (Figure 7C). No further reduction in binding was observed for the interaction of FBN1-R232E and LTBP1-D1655R constructs (Figure S5); if these residues acted independently, as part of two separate salt bridges, then an additive effect and a further reduction in binding would be expected (Hwang and Warshel, 1988, Venkatachalan and Czajkowski, 2008). A similar observation is made for the FBN1-R182E/LTBP1-D1573R interaction. These results are consistent with specific salt bridges forming between R232 and D1655 and between R182 and D1573 in the interaction of FBN1 with LTBP1 (Figure 7C).

**Figure 8:**
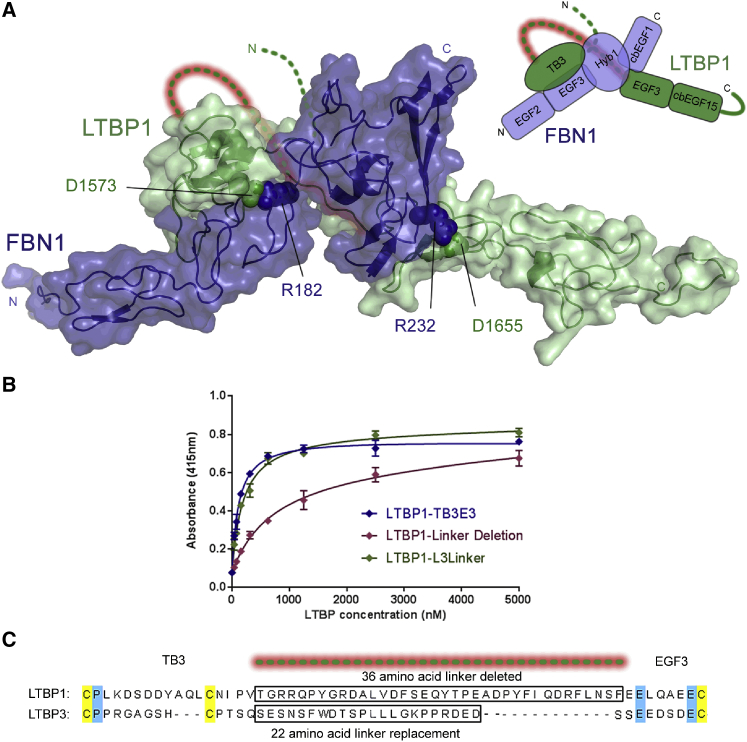
Model of LTBP1-FBN1 Interaction Derived from HADDOCK and Role of LTBP1 TB3-EGF3 Linker in the Interaction (A) Combined interaction model for FBN1 and LTBP1 created by merging the best models calculated for the two interaction sites. The EGF2-EGF3-Hyb1-cbEGF1 domains of FBN1 are shown in blue and the TB3-EGF3-cbEGF15 domains of LTBP1 are shown in green. The side-chain atoms of residues that form salt bridges are shown as spheres. The dotted lines represent one of many possible positions for the flexible linkers that in LTBP1 join the C terminus of cbEGF14 to the N terminus of TB3 (green dotted line) and the C terminus of TB3 to the N terminus of EGF3 (green dotted line highlighted in red). Inset: cartoon summarizing the layout of protein domains shown. (B) Plate-binding assay, with the FBN1^E2cbEGF1^ construct immobilized on the plate surface, shows the effect of modifying the TB3-EGF3 linker in LTBP1. Deletion of the linker in LTBP1^TB3E3^ leads to a weakening of the interaction in comparison with WT LTBP1. Replacement of the LTBP1 linker with the LTBP3 linker shows binding similar to the WT construct. The data shown here are representative; they are taken from a single plate with three repeats of each protein concentration carried out to determine experimental error (SD). (C) Sequence alignment of the TB3-EGF3 region of LTBP1 and LTBP3 shows little or no sequence homology between the two linker regions. Regions of the linker deleted or replaced are highlighted by boxes. See also Figures S1 and S6; Table S1; Data S1.

The identification of residues in salt bridge interactions stabilizing the complex allowed refinement of the HADDOCK models by including specific distance restraints in each calculation. Figure 8A shows an overall model of the complex created by splicing together the best clusters obtained from docking of LTBP1 TB3 and EGF3-cbEGF15 with FBN1^E2cbEGF1^. In the HADDOCK model of the FBN1/LTBP1 complex, the distance separating the last residue of LTBP1-TB3 and the first residue of LTBP1-EGF3 is small enough to be easily accommodated by the 36-residue flexible linker joining the two domains (Figure 8A). An LTBP1^TB3E3^ variant in which this linker was deleted showed lower binding to FBN1^E2cbEGF1^ in a plate-based assay (Figures 8B and 8C), consistent with only one of the two LTBP1 domains being able to interact at any given time in this construct. Replacement of the LTBP1 linker with the shorter 22-residue linker from LTBP3, which has a very different amino acid sequence, restored binding to levels comparable with the WT interaction indicating the linker plays a passive role as a connector (Figures 8B and 8C).

### Biological Significance of FBN/LTBP Interaction

**Figure 9:**
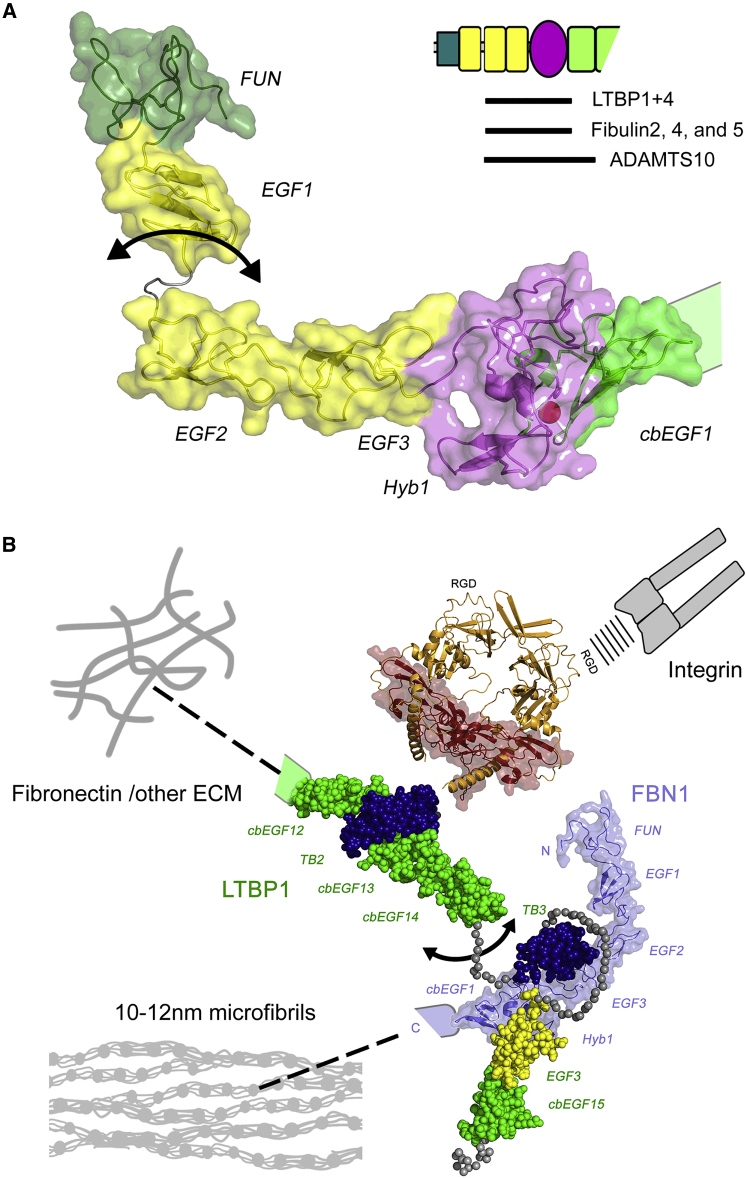
Molecular Model for Presentation of TGF-β by LTBP1 in the ECM (A) Structure of the FBN1 N terminus constructed from the structure presented here (5MS9) and the previously published structure of NE3 (2M74). The linker between EGF1 and EGF2 is flexible on a fast timescale as shown by an arrow. Domains are colored as in Figure 1 and the top right inset shows the minimal regions required for specific protein interactions. (B) Model of LAP and fibrillin binding of LTBP1S constructed using the program Modeller and coordinate files PDB: 1UZJ, 1EMN, and 1KSQ. LTBP1 atoms are represented as spheres colored by domain as in Figure 1, while FBN1 is colored slate blue and shown as a ribbon with transparent surface rendering. For the known flexible linkers in LTBP1 only the Cα atoms are shown (gray spheres). For scale, the structure of TGF-β (dark red ribbon and surface) bound to LAP (orange ribbon) from PDB: 3RJR (Shi et al., 2011) is included next to the second TB domain of LTBP1, which binds the small latent complex covalently via a disulfide linkage. LTBP1 can also bind to other ECM fibers via its N terminus (Robertson et al., 2015), and to cell-surface integrins via the RGD motif in LAP. Integrins are thought to bind LAP in order to exert traction and release TGF-β.

Our solution structure of a four-domain FBN1 fragment, EGF2-EGF3-Hyb1-cbEGF1, identifies a near-linear domain organization, which, together with the previously determined NE3 structure, reveals the complete structural organization of the N-terminal region of FBN1 (Figure 9A). The extended shape of FBN1, but with a flexible linker between EGF1 and EGF2, may help expose multiple binding sites that allow FBN1 to act as an interaction hub, with numerous protein-protein interactions reported for this region including LTBP1 and 4 (Ono et al., 2009), fibulin-2, -4, and -5 (Choudhury et al., 2009, El-Hallous et al., 2007, Ono et al., 2009), and ADAMTS10 (Kutz et al., 2011).

Our detailed dissection of LTBP1 binding to FBN1 EGF2-EGF3-Hyb1-cbEGF1 has identified a bipartite mode of interaction, with the C-terminal region of LTBP1 utilizing two discrete sites to anchor itself to FBN1. The two orders of magnitude change in K_d_ seen when the binding of LTBP1^TB3E3^ (∼0.5–1 μM), which contains two binding sites, is compared with the binding of the LTBP1^E3cbEGF15^ and LTBP1^cbEGF14TB3^ fragments (∼100 and ∼300 μM, respectively), which contain single binding sites, is similar to other bipartite interactions mediated by modular proteins. For example, in a collagen/fibronectin interaction, two individual sites with K_d_ values of 2 mM and 248 μM, combine to give a K_d_ of 26 μM (Erat et al., 2013). For fibronectin F1 modules which interact with streptococcal SfbI peptides, two sites with K_d_ values of 159 and 63 μM combine to give a K_d_ of 0.45 μM (Schwarz-Linek et al., 2004).

While our measured binding affinities for LTBP fragments, in simple assays, are relatively weak, these affinities are not atypical of other modular protein interactions. Our estimated K_d_ of ∼0.5–1 μM for the FBN1^E2cbEGF1^/LTBP1^TB3E3^ interaction represents a 4- to 25-fold weaker binding than reported in previous SPR studies; the variation in reported K_d_ values may arise from methodological differences (Massam-Wu et al., 2010, Ono et al., 2009). Since modular ECM proteins often multimerize or have multiple binding sites for proteins/proteoglycans/glycosaminoglycans, additional strategies are likely to further increase binding of LTBP to the matrix. Recently multimerization of LTBP1 has been observed, which is enhanced by heparin (Troilo et al., 2016). Higher-order assembly of LTBP1, together with the bipartite interaction reported here, may allow LTBP1 to achieve a strong interaction with FBN1 in the dynamic environment of connective tissues, while maintaining contacts with other ECM protein networks and/or cell/matrix components (Figure 9B).

It has been reported previously that the interaction between FBN1 and LTBP1 is much stronger than that involving FBN2 and LTBP1 (Isogai et al., 2003). Comparison of the sequences of human FBN1 and FBN2 shows complete conservation of residues involved in the stronger FBN Hyb1-cbEGF1/LTBP1 EGF3 interface, whereas three substitutions are observed in the weaker FBN EGF3-Hyb1/LTBP1 TB3 interface (Figure S7). A reduction of binding affinity in the latter site could explain the observed differences in binding for FBN1/FBN2. It has also been suggested that the mode of interaction of FBN1 with the LTBP1 and LTBP4 isoforms differs (Ono et al., 2009). Comparison of the sequences of human LTBP1 and LTBP4 shows substitutions in both binding sites (Figure S7). Some of these substitutions alter the electrostatic properties of the LTBPs. Interestingly, an N164S substitution in FBN1 EGF3, associated with dominant ectopia lentis (Comeglio et al., 2002), has been reported previously to decrease the binding affinity of FBN1 to LTBP4, but not to LTBP1 (Ono et al., 2009). N164 is not directly involved in the LTBP1 binding site in our model of the complex; instead its side chain is located on the opposite face of the FBN1 EGF3 domain. The reduction in binding to LTBP4 suggests that the binding interface must be different in the FBN1/LTBP4 complex.

To conceptualize the significance of the LTBP1-FBN1 interaction, a large-scale model of LTBP1 was generated, with the small latent complex of LAP/TGF-β also included for scale (Figure 9B). It can be seen that the short flexible linker identified previously between cbEGF14 and TB3 in LTBP1 (Robertson et al., 2014) may serve as an omnidirectional pivot that helps LTBP to maintain its contacts with other networks and to orient latent TGF-β for activation by integrins or other factors. It is possible that FBN microfibrils (together with other ECM networks) contribute directly to the efficiency of activation of TGF-β since the unusual bipartite nature of the LTBP interaction may allow the LTBP/FBN complex to resist integrin-mediated traction or other dynamic changes in connective tissue, while TGF-β is released from the LAP complex. Integrin-mediated activation of TGF-β has been shown to be heavily reliant on matrix biomechanics (Buscemi et al., 2011, Klingberg et al., 2014), and at present integrins are the only TGF-β activators that have been clearly demonstrated to be crucial *in vivo* (Robertson and Rifkin, 2014, Yang et al., 2007). In Marfan syndrome, loss-of-function *FBN1* mutations result in a deficiency of 10–12 nm microfibrils in the ECM. While LTBP may still be able to bind to other ECM networks such as fibronectin, despite the reduction in FBN, ECM biomechanics may be altered sufficiently in the absence of microfibrils that TGF-β is inappropriately released from the LAP complex when integrins bind. This might explain why *in vivo* deletion of the Hyb1 domain, which does not impair microfibril assembly and contains the major binding site for LTBP1, did not result in any Marfan-like phenotypes (Charbonneau et al., 2010).

Proteases like BMP1 have been proposed to regulate TGF-β signaling during normal development by cleaving the C terminus of LTBP1 and releasing it from the ECM (Ge and Greenspan, 2006). The presence of a flexible linker between the two FBN1-interacting sites within LTBP1 suggests a mechanism by which proteases can regulate the affinity of this interaction. Protease cleavage of the TB3–EGF3 linker in LTBP1 may release the large latent complex from the 10–12 nm microfibril network, as the affinity of the TB3 binding site alone for FBN1 is very much weaker than the bipartite interaction. Cleavage may also significantly reduce the affinity of the remaining EGF3-cbEGF15 LTBP1 fragment for FBN1, facilitating turnover.

In summary, we have completed the structure of the FBN1 N-terminal region, explaining its role as an interaction hub in the ECM. We have further demonstrated an unusual bipartite interaction of LTBP1 with FBN1, adjacent to the LAP/TGF-β binding site, which facilitates complex formation in dynamic connective tissues. We propose that this contributes to integrin-mediated activation of TGF-β in FBN1-rich tissues.

## STAR★Methods

### Key Resources Table

**Table:** 

REAGENT or RESOURCE	SOURCE	IDENTIFIER
**Antibodies**		

anti-RGS-His antibody conjugated with HRP	Qiagen	34450

**Bacterial and Virus Strains**		

BL21 pREP4	Lab strain	N/A
XL10 Gold	Agilent	200315

**Chemicals, Peptides, and Recombinant Proteins**		

FBN1^NE3^	(Yadin et al., 2013)	N/A
FBN1^E2cbEGF1^ and variants	This work and (Robertson et al., 2013b)	N/A
FBN1^cbEGF22TB4cbEGF23^	(Jovanovic et al., 2007)	N/A
LTBP1^TB3cbEGF15^	This work	N/A
LTBP1^TB3E3^ and variants	This work and (Robertson et al., 2014)	N/A
LTBP1^cbEGF14TB3^	This work and (Robertson et al., 2014)	N/A
LTBP1^E3cbEGF15^	This work and (Robertson et al., 2014)	N/A
^15^N ammonium chloride	Goss Scientific	NLM-467
^13^C Glucose	Goss Scientific	CLM-1396-5
D_2_O	Sigma Aldrich	151882-125 G
Chelating Sepharose	GE Healthcare	17-0575-01

**Deposited Data**		

BMRB NMR assignments	(Robertson et al., 2013a, Robertson et al., 2014, Robertson et al., 2013b)	19078, 18848, 19322
Fibrillin E2cbEGF1 NMR structure	This paper	5MS9

**Oligonucleotides**		

18 Site directed mutagenesis primers listed in Table S2	This work	N/A

**Recombinant DNA**		

FBN1^NE3^ – in pQE30 expression vector	(Yadin et al., 2013)	N/A
FBN1^E2cbEGF1^ and variants - in pQE30 expression vector	This work and (Robertson et al., 2013b)	N/A
FBN1^cbEGF22TB4cbEGF23^ – in pQE30 expression vector	(Jovanovic et al., 2007)	N/A
LTBP1^TB3cbEGF15^ – in pQE30 expression vector	This work	N/A
LTBP1^TB3E3^ and variants – in pQE30 expression vector	This work and (Robertson et al., 2014)	N/A
LTBP1^cbEGF14TB3^ – in pQE30 expression vector	This work and (Robertson et al., 2014)	N/A
LTBP1^E3cbEGF15^ – in pQE30 expression vector	This work and (Robertson et al., 2014)	N/A

**Software and Algorithms**		

ARIA 2.3	(Rieping et al., 2007)	N/A
Xplor-NIH 2.29	(Schwieters et al., 2003)	N/A
MolProbity	(Chen et al., 2010)	N/A
HADDOCK server	(van Zundert et al., 2016)	N/A
TALOS+	(Shen et al., 2009).	N/A
ATSAS software package	(Petoukhov et al., 2012)	N/A
CCPN software	(Vranken et al., 2005)	N/A
NMRPipe	(Delaglio et al., 1995)	N/A

**Other**		

ELISA plates	R&D systems	DY990
Biacore SPR CM5 sensor chips	GE healthcare	BR100530

### Contact for Reagent and Resource Sharing

Further information and requests for resources and reagents should be directed to and will be fulfilled by the Lead Contacts, Penny Handford and Christina Redfield (penny.handford@bioch.ox.ac.uk and christina.redfield@bioch.ox.ac.uk).

### Experimental Model and Subject Details

#### Strains Used in Protein Production

The FBN1 and LTBP1 protein fragments used in this study were expressed in *Escherichia coli* BL21 cells transformed with a pQE-30 (Qiagen) expression vector and pREP4 plasmid for control of expression via the lac repressor. When cloned into the expression vector, an N-terminal His_6_ tag was included for purification, followed by an Ser-Ala spacer and a factor Xa protease recognition site (Ile-Glu-Gly-Arg) for later removal of the His_6_ tag.

### Method Details

#### Protein Production and Purification

Sequences encoding the EGF2-EGF3-Hyb1-cbEGF1 region of human *FBN1* and the cbEGF14-TB3-EGF3-cbEGF15 region of human *LTBP1* were cloned into the pQE30 vector (Qiagen). The additional cysteine in the FBN1 Hyb1 domain (C204) was replaced with a serine as described previously (Jensen et al., 2009). This change was necessary to allow effective in vitro refolding of the FBN1 protein fragment. Site-directed mutagenesis was carried out using the QuikChange protocol (Agilent) (for details of primers used see Table S2).

Protein expression and purification was carried out in a similar fashion to that described previously (Knott et al., 1996) but with modifications for each construct (Robertson et al., 2013a, Robertson et al., 2013b, Robertson et al., 2014, Yadin et al., 2012). Unlabelled proteins were expressed in *E. coli* grown on unlabelled rich medium. Proteins were single or double labelled with ^15^N or ^15^N/^13^C by growing cells in M9 medium containing 0.1% (w/v) ^15^NH_4_Cl and 0.5% (w/v) ^13^C-glucose (Goss Scientific), in the presence of 100 μg/ml ampicillin and 25 μg/ml kanamycin. 50 ml of starter culture, grown in unlabelled M9 medium at 37 °C for ∼18 hours, was used to inoculate 600 ml of labelled M9 medium. Bacteria were grown until OD_600_ reached ∼0.8, at which point expression was induced with isopropyl-β-D-thiogalactopyranoside (IPTG) at a final concentration of 1 mM. Cells were then incubated at 28 °C for ∼20 hours, harvested by centrifugation and frozen at −80 °C prior to protein purification.

Proteins were purified from inclusion bodies (Robertson et al., 2013a, Robertson et al., 2013b, Robertson et al., 2014, Yadin et al., 2012). The solubilised His-tagged proteins were purified using Ni^2+^ affinity chromatography using fast-flow chelating Sepharose (Amersham Pharmacia or GE Healthcare). His-tagged proteins were eluted with buffer containing 50 mM EDTA. The eluted proteins were then reduced with ∼200 mM DTT, buffered with 0.1 M Tris-HCl pH 8.3 and left for at least 1 hr at room temperature to allow for full reduction of the protein. The reduced protein solutions were acidified to pH 2-3 with HCl and dialysed overnight at room temperature against 2 L of 0.1% trifluoroacetic acid (TFA). Protein was then desalted by high performance liquid chromatography (HPLC) using a C8 reverse phase column (Rainin).

Purified, reduced proteins were refolded in an aqueous solution of ∼0.2 mg/ml reduced protein, 100 mM Tris-HCl pH 8.3, 3 mM cysteine and 0.3 mM cystine, and up to 50 mM CaCl_2_; 50% (v/v) glycerol was included for refolding of FBN1^E2cbEGF1^ and FBN1^NE3^ (to enhance protein solubility) but was not necessary for refolding of LTBP1 constructs. The solution was then left for 48-72 hrs at 4°C. After this period the refold mixture was acidified to pH 2-3 with HCl, and dialysed against 0.1% (v/v) TFA overnight. Dialysate was centrifuged and filtered to remove any precipitate, concentrated by ultrafiltration, filtered again, and then purified by HPLC.

The His_6_ tag was cleaved off for all constructs, except LTBP1^TB3E3^ and LTBP1^TB3cbEGF15^, by incubation with factor Xa (Novagen), carried out with a protein concentration of 1.5-5 mg/ml and 1 unit factor Xa per mg protein, and incubated at 37 °C overnight. Proteins were further purified by fast protein liquid chromatography (FPLC) using a MonoS 5/50 GL or a MonoQ 5/50 column (GE Healthcare) depending on protein solubility. After FPLC all proteins were acidified to pH ∼2, filtered to remove any precipitate, and desalted by further HPLC purification, before final lyophilisation. The final products were analysed by SDS-PAGE (Figure S1), electrospray ionisation mass spectrometry, and 2D ^1^H-^1^H NOESY spectra, and were shown to be monomeric and correctly folded.

#### Protein Interaction Experiments

Plate-based interaction experiments were carried out by incubating DY990 plates (R&D systems) with 50 μl FBN1^E2cbEGF1^ in 50 mM carbonate buffer at pH 9.6 for a minimum of 48 hours. Plates were then blocked with 5% BSA carbonate buffer for one hour. Wells were washed with 100 μl interaction buffer (50 mM Tris pH 7.5, 150 mM NaCl, 0.05% (v/v) Tween-20, 2 mM CaCl_2_) and 50 μl LTBP1 protein samples were then added. At this stage wells were aspirated and aliquoted individually to avoid plate drying. Plates were incubated with LTBP1 samples for 12 hours and then washed. A 1:5000 dilution of an anti-RGS-His antibody conjugated with HRP (Qiagen 34450) was then added to each well and incubated for 1 hour to detect LTBP1 binding. Data presented in figures are representative examples of several experiments carried out at different times (for additional information see QUANTIFICATION AND STATISTICAL ANALYSIS section below).

SPR studies were carried out using a Biacore T100 instrument (GE Healthcare) with FBN1 fragments coupled to the surface of a CM5 sensor chip by amine coupling and then washed with 50 mM HCl. The Biacore coupling wizard was used to ensure that an equivalent 1000 RU of protein was coupled to each flow cell. The sensor chip was equilibrated with SPR running buffer consisting of 50mM Tris pH 7.5, 150mM NaCl, 0.05% (v/v) Tween 20 and 2mM CaCl_2_. Freeze-dried LTBP1 proteins were dissolved directly into SPR buffer to generate analyte stock solutions. The protein concentrations in these stock solutions were measured by UV-visible spectroscopy. All SPR experiments were performed at 25°C, with a flow rate of 20 μl per minute, and a 30 second 50mM HCl injection was used for regeneration after each run. Both Multi-cycle and single-cycle programs were used depending on program availability and the volume of data needed. All four flow cells of the chip were used, one as a blank for baseline subtraction, one with the FBN1^E2cbEGF1^ fragment bound, one with FBN1^NE3^ bound, and finally one with a negative control FBN1 fragment spanning the cbEGF22-TB4-cbEGF23 domains bound. In this way all flow cells were simultaneously exposed to the same LTBP1 protein analyte solutions. K_d_ values were estimated, where possible for lower affinity interactions, from linear Scatchard plots. In some cases, despite the clear specificity of binding (shown by lack of binding to a control FBN1 fragment or the blank and control treated flow cells), the Scatchard plots were non-linear; this is not surprising because the multi-site mode of interaction for the higher affinity LTBP1 constructs may give rise to complicated binding kinetics.

#### NMR Spectroscopy

NMR experiments were carried out using spectrometers operating at ^1^H frequencies ranging from 500 to 950 MHz. The spectrometers were equipped with Oxford Instruments magnets and home-built triple-resonance pulsed-field gradient probes (500, 600, 750 and 950 MHz) or with Bruker Avance consoles and TCI CryoProbes (500 and 750 MHz). NMR data were acquired using either GE/Omega software using pulse sequences written in-house, or Topspin software and pulse sequences in the Topspin libraries from Bruker Biospin. NMR data were processed using NMRPipe (Delaglio et al., 1995) and spectra were analysed using the CCPN software (Vranken et al., 2005).

##### Resonance Assignments

Resonance assignments for FBN1^E2cbEGF1^, LTBP1^E3cbEGF15^ and LTBP1^cbEGF14TB3^ have been described previously (Robertson et al., 2013a, Robertson et al., 2013b, Robertson et al., 2014) (BMRB accession numbers 19078, 18848, 19322, respectively). Unless otherwise stated, all NMR experiments were carried out at 25°C at pH 5.4 in 95% H_2_O/5% D_2_O with 5mM CaCl_2_.

##### Protein Interactions Monitored by NMR

Interactions between LTBP1 and FBN1 fragments were monitored using 2D ^1^H-^15^H HSQC spectra collected at 500 MHz. Initially an HSQC spectrum was collected for the ^15^N-labelled protein sample alone, and then successive freeze dried aliquots of the unlabelled interaction partner were added, with the pH of the NMR sample measured before and after each addition, and adjusted prior to running each HSQC experiment. Initial experiments utilising the LTBP1^TB3E3^ and FBN1^E2cbEGF1^ fragments were not informative because uniform broadening of all signal in the HSQC was observed, most likely due to slow exchange effects caused by strong binding of the large LTBP1 fragment.

The interaction of LTBP1^E3cbEGF15^ with FBN1^E2cbEGF1^ was monitored in two titrations using either ^15^N-labelled LTBP1^E3cbEGF15^ or ^15^N-labelled FBN1^E2cbEGF1^. In both titrations 300 μl of a 300 μM ^15^N sample was used at pH 5.5, with 100 mM NaCl and 10 mM CaCl_2_. The concentration of the unlabelled LTBP1^E3cbEGF15^ or FBN1^E2cbEGF1^ ligand ranged from 0 to 250 μM. In these titrations, peaks were observed to shift; the combined chemical shift change, reported in Figure 3, was determined as Δ_COMB_=((Δ_1HN_)^2^+(Δ_15N_/6)^2^)^1/2^ where Δ_1HN_ and Δ_15N_ are the observed chemical shift differences for ^1^H^N^ and ^15^N in HSQC spectra collected with 0 and 250 μM ligand protein.

The interaction of LTBP1^cbEGF14TB3^ with FBN1^E2cbEGF1^ was monitored in two titrations using either ^15^N-labelled LTBP1^cbEGF14TB3^ or ^15^N-labelled FBN1^E2cbEGF1^. In the first titration 300 μl of a 300 μM ^15^N-LTBP1^cbEGF14TB3^ sample was used at pH 5.9 with 20 mM CaCl_2_. In the second titration 290 μl of a 300 μM ^15^N FBN1^E2cbEGF1^ sample was used at pH 5.3 with 15 mM CaCl_2_ (slight differences in conditions were necessary to optimise protein solubility). The concentration of the unlabelled LTBP1^cbEGF14TB3^ or FBN1^E2cbEGF1^ ligand ranged from 0 to 250 μM. In these titrations specific losses in peak intensity were seen with successive protein additions. To quantify peak intensity changes during the titration, peak intensities in the first HSQC experiment without added ligand protein were divided by the peak intensities in the final titration point. Errors bars represent the error introduced by background noise in the spectrum.

##### Heteronuclear NOE

{^1^H}-^15^N heteronuclear NOE experiment was carried out using ^15^N-labelled FBN1^E2cbEGF1^ in order to examine the sub-nanosecond dynamics of specific amides (Kay et al., 1989). Spectra with and without ^1^H saturation were collected as interleaved experiments collected at 750 MHz. ^1^H saturation was applied for 4 s. The data set was acquired with 1K complex points in F_2_ and 128 complex t_1_ increments; 96 scans were collected per increment. The {^1^H}-^15^N NOE was calculated as the ratio of the peak intensities in the spectra recorded with and without ^1^H saturation. Peak heights were determined using in-house peak-picking software. Uncertainties in the NOE ratios were estimated from 500 Monte Carlo simulations using baseline noise as a measure of the error in the peak heights.

##### Residual Dipolar Couplings

Residual dipolar couplings (RDCs) were collected for the FBN1^E2cbEGF1^ construct in two liquid crystalline media. One set of RDCs was collected with a 2.2% C12E6/*n*-hexanol (Sigma-Aldrich) solution in 90% H_2_O/10% D_2_O at pH 5.3 with 5mM calcium chloride (Rückert and Otting, 2000). The second set of RDCs was collected with 4% (w/v) bicelles comprising the ether linked lipids 1,2-O-ditridecyl-*sn*-glycero-3-phosphocholine and 1,2-dihexyl-*sn*-glycero-3-phospho-choline (Avanti Polar Lipids), as well as cetyl trimethyl ammonium bromide (Sigma-Aldrich) in a molar ratio of 35:10:1 in 90% H_2_O/10% D_2_O at pH 5.5 with 5mM calcium chloride (Ottiger and Bax, 1998, Ramirez and Bax, 1998). Interleaved IPAP experiments (Ottiger et al., 1998) were performed at a ^1^H frequency of 600 MHz at 25°C (or 35°C for the bicelles) using 128 and 1024 complex points in F_1_ (^15^N) and F_2_ (^1^H), respectively. Isotropic spectra were also collected under comparable conditions and residual dipolar couplings were measured as the difference between the splitting observed in the isotropic and aligned data sets.

#### SAXS

Small Angle X-ray Scattering data were collected at beamline BM29 at the ESRF in Grenoble, France. Lyophilised protein was dissolved in buffer containing 20 mM MES at pH 5.4, 5 mM CaCl_2_, and 5% glycerol. This solution was then further dialysed against a large volume of the same buffer and a sample of this buffer used as a blank in the beam line to ensure correct matching. Scattering data were collected at four different protein concentrations and the data from these samples were scaled and averaged for further analysis. Guinier analysis was performed using the SCATTER software package and *ab initio* modelling and structure fitting was performed using the DAMMIF and CRYSOL programs in the ATSAS software package (Petoukhov et al., 2012) *Ab initio* modelling was performed using ‘slow’ mode with 20 repetitions and default settings for modelling globular proteins. When NMR structures were fitted to the SAXS data using CRYSOL the 7 flexible N-terminal residues of FBN^E2cbEGF1^ were removed as this random unstructured element could significantly affect the fitting.

#### Structure Determination

NMR experiments for structure determination were carried out using ^15^N or ^13^C/^15^N labelled FBN1^E2cbEGF1^ at a concentration of 1 mM at pH 5.4 and 25°C. Distance restraints for structure calculation were derived from several 2D and 3D NOESY spectra. The 3D ^15^N-edited NOESY-HSQC spectrum (mixing time 150 ms) was acquired at 950 MHz in 95% H_2_O/5% D_2_O. 3D ^13^C-edited NOESY-HSQC spectra (mixing time 75 ms) were acquired at 500 and 950 MHz in 95% H_2_O/5% D_2_O (v/v) and 100% D_2_O, respectively. An aromatic 3D ^13^C-edited NOESY-HSQC spectrum (mixing time 150 ms) was acquired at 750 MHz in 95% H_2_O/5% D_2_O (v/v). A 2D ^1^H-^1^H NOESY spectrum (mixing time 150 ms) was acquired at 950 MHz in 100% D_2_O. NOESY cross peaks were assigned manually in CCPN Analysis using published resonance assignments (Robertson et al., 2013b). In some cases only ambiguous peak assignments were possible initially.

ϕ and ψ torsion angle restraints were obtained using TALOS+ predictions on the basis of assigned chemical shifts (Shen et al., 2009). Hydrogen bond restraints were based on slowly exchanging amides identified in HSQC spectra collected in D_2_O and observed NOEs characteristic of regular secondary structure.

Comparison of the distribution of RDC values for the EGF2-EGF3 pair and the Hyb1-cbEGF1 pair showed that they are not the same indicating that some slower timescale movement of the domain pairs relative to each other may exist in solution (Figure 5B); this was the case for both alignment media used. In order not to bias the definition of the EGF3-Hyb1 interface in structure calculations, the RDC data for the EGF2-EGF3 and Hyb1-cbEGF1 pairs were treated separately in the structure calculations (i.e. different reference alignment tensors used). Values for the axial and rhombic components for the alignment tensors were calculated from the previously determined structure of NE3 and from a homology model for Hyb1-cbEGF1 using in-house software. For the EGF2-EGF3 pair D_a_/R values of -13.9/0.56 were used for the bicelle data and values of -19.0/0.54 were used for the C12E6/n-hexanol data. For the Hyb1-cbEGF1 pair D_a_/R values of 11.2/0.35 were used for the bicelle data and values of 15.7/0.50 were used for the C12E6/n-hexanol data.

Structures were calculated initially using ARIA 2.3 (Rieping et al., 2007); this was useful for obtaining assignments for ambiguous NOEs and for validating the allocation of NOE restraints into distance bins. However, the four-domain topology and the 13 disulphide bonds in FBN1^E2cbEGF1^ resulted in a limited number of converged structures using this protocol. Subsequent structure calculations were performed using Xplor-NIH 2.29 (Schwieters et al., 2003). Initially, ∼800 structures were calculated from an extended starting structure using a simulated annealing protocol (initial T=2000K, 30000 high temperature steps, 40000 and 20000 steps in cooling to 1000K and 100K, respectively) using NOE, hydrogen bond, disulphide bond and torsion angle restraints. The Ca^2+^-binding site was defined using distance restraints between the Ca^2+^ ion and the cbEGF consensus ligands (Downing et al., 1996). A 'Rama' torsion angle database potential was used (Kuszewski et al., 1996). The 20 lowest energy simulated annealing structures were used as the starting point for refinement (initial T=1500K, 20000 cooling steps) generating a total of 400 structures; the 5 lowest energy structures from each of the 20 starting structures were selected for further refinement using the two sets of RDC restraints. Refinement was carried out using 100 starting structures (initial T=1500K, 20000 cooling steps). A square well potential and a force constant of 0.5 were used for the SANI terms with experimental error for the RDCs in the range of 2-4 Hz. 40 structures were selected from the family of 2000 RDC-refined structures for a final round of water refinement. The water-refined structures were used to predict the SAXS data and a final family of 20 structures was chosen on the basis of low restraint and overall energies and a good fit to the SAXS data. Ramachandran validation statistics were calculated using MolProbity (Chen et al., 2010).

#### Modelling the FBN1/LTBP1 Complex Using HADDOCK

Docking was carried out using the ‘guru’ interface of the HADDOCK server (van Zundert et al., 2016). Since the TB3 and EGF3 domains of LTBP1 are connected by a flexible linker and their binding sites were mapped separately using the LTBP1^cbEGF14TB3^ and LTBP1^E3cbEGF15^ constructs, HADDOCK modelling of these two interactions was performed separately. Flexibility was introduced into all protein models, both to account for observed flexible loops and also structural ambiguity. A large number of preliminary calculations was performed using different sets of ambiguous interaction restraints defined using different cut offs applied to the NMR titration data. From these calculations numerous models were generated that were clustered and sorted on the basis of various shared features. The results suggested several possible binding orientations, but one common feature in the models was the presence of salt bridges that were frequently seen between basic residues in FBN1 and acidic residues in LTBP1. In the different binding orientations identified, each employed different charged residues in the formation of inter-molecular salt bridges, allowing the design of targeted substitutions to test these HADDOCK models. Substitutions introduced were K138D, R182E, and R232E in FBN1^E2cbEGF1^, and D1521K, D1573R, E1625R, E1642R, and D1655R in LTBP1^TB3E3^. Binding of the substituted FBN1 and LTBP1 constructs was assessed using the SPR and plate-based assay as described above for the wild-type constructs.

A final round of HADDOCK calculations was carried out using the restraints derived from the NMR data and the salt bridges identified by the mutagenesis experiments. The final docking clusters shown in Figure 8, and included as a supplemental pdb file (Data S1), were based on the docking parameters shown in Table S1. Active and passive residues used to define the ambiguous interaction restraints were derived from the peak shift and intensity change data shown in Figure 3. Random exclusion of AIR restraints was allowed with 10 partitions (10% of restraints). All molecules were modelled as ‘semi-flexible’ and specific segments listed in the table were modelled as fully flexible to reflect heteronuclear NOE data and structural ambiguity. Histidine protonation was determined automatically using MolProbity (Chen et al., 2010).

### Quantification and Statistical Analysis

Plate-based assay data to probe protein-protein interactions presented in figures are from a single plate; three repeats of each protein concentration were carried out to determine experimental error. The data presented are representative examples of several experiments carried out at different times. The curves fitted to the data were generated using GraphPad with the ‘One site – Total binding’ option. This takes into account specific binding for which a K_d_ is fitted, nonspecific binding which is assumed to have a linear dependence on ligand concentration, and background signal. The K_d_ values extracted are apparent K_d_s because the fitting procedure uses total ligand concentration rather than free ligand concentration.

### Data and Software Availability

#### Data Resources

The coordinates of the family of NMR structures of FBN1^E2cbEGF1^ have been deposited in the Protein Data Bank under accession number 5MS9 (see Table 1). Resonance assignments for FBN1^E2cbEGF1^, LTBP1^E3cbEGF15^ and LTBP1^cbEGF14TB3^ have deposited in the BioMagResBank (BMRB) under accession numbers 19078, 18848 and 19322, respectively. A PDB model of the LTBP1-FBN1 interaction, produced by splicing together the two highest scoring HADDOCK result files, is included in the Supplemental Information.

## Author Contributions

P.A.H., S.A.J., C.R., and I.B.R. designed the study. I.B.R., H.D., and I.H.O. produced recombinant protein samples and performed the plate-binding and SPR experiments. I.B.R. and C.R. performed the NMR experiments and the solution structure calculations. E.D.L. and I.B.R. performed the SAXS experiments. I.B.R. performed the docking calculations. P.A.H., C.R., and I.B.R. analyzed the results and wrote the manuscript. All authors discussed the results and implications and commented on the manuscript at all stages.

## Accession Numbers

The coordinates of the family of NMR structures of FBN1^E2cbEGF1^ have been deposited in the PDB under accession number PDB: 5MS9.

## Supplemental Information

Document S1. Figures S1–S7 and Tables S1 and S2

Data S1. PDB Model of the LTBP1-FBN1 Complex Produced by Splicing Together the Two Highest Scoring HADDOCK Models, Related to Figure 8

Document S2. Article plus Supplemental Information
