# Supplementary material for: The N-Terminal Region of Fibrillin-1 Mediates a Bipartite Interaction with LTBP1
Source: Structure. 2017 Aug 1;25(8):1208–1221.e5. doi: 10.1016/j.str.2017.06.003 (PMC5548924; doi:10.1016/j.str.2017.06.003)
Supplement: Document S1. Figures S1–S7 and Tables S1 and S2 [file mmc1.pdf]

Structure, Volume *25*

Supplemental Information

The N-Terminal Region of Fibrillin-1 Mediates a Bipartite Interaction with LTBP1

Ian B. Robertson, Hans F. Dias, Isabelle H. Osuch, Edward D. Lowe, Sacha A. Jensen, Christina Redfield, and Penny A. Handford

Supplemental Figures

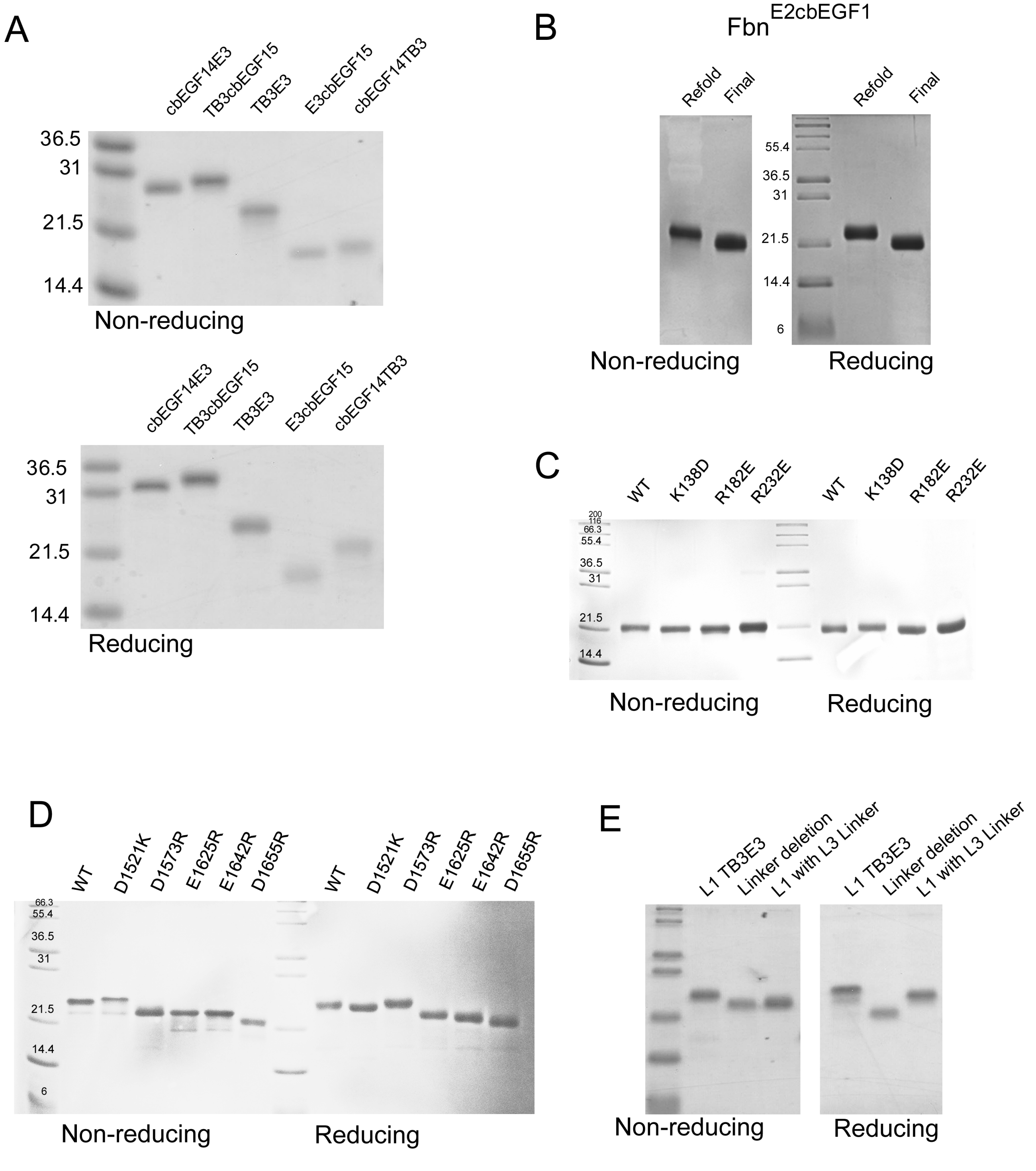

Figure S1. Related to Figure 1, 2, 7 and 8. SDS-PAGE of constructs used in this study. Coomassie stained SDS-PAGE of protein fragments used in this study. Both non-reducing and reducing gels are shown. (A) Wild-type LTBP1 constructs. (B) FBN1 EGF2-EGF3-Hyb1-cbEGF1 construct. (C) FBN1 variants used to test salt bridges. (D) LTBP1 variants used to test salt bridges. (E) LTBP1 linker variants. All constructs show a good degree of purity under both reducing and non-reducing conditions. Mark 12 (Invitrogen) was used as the protein standard.

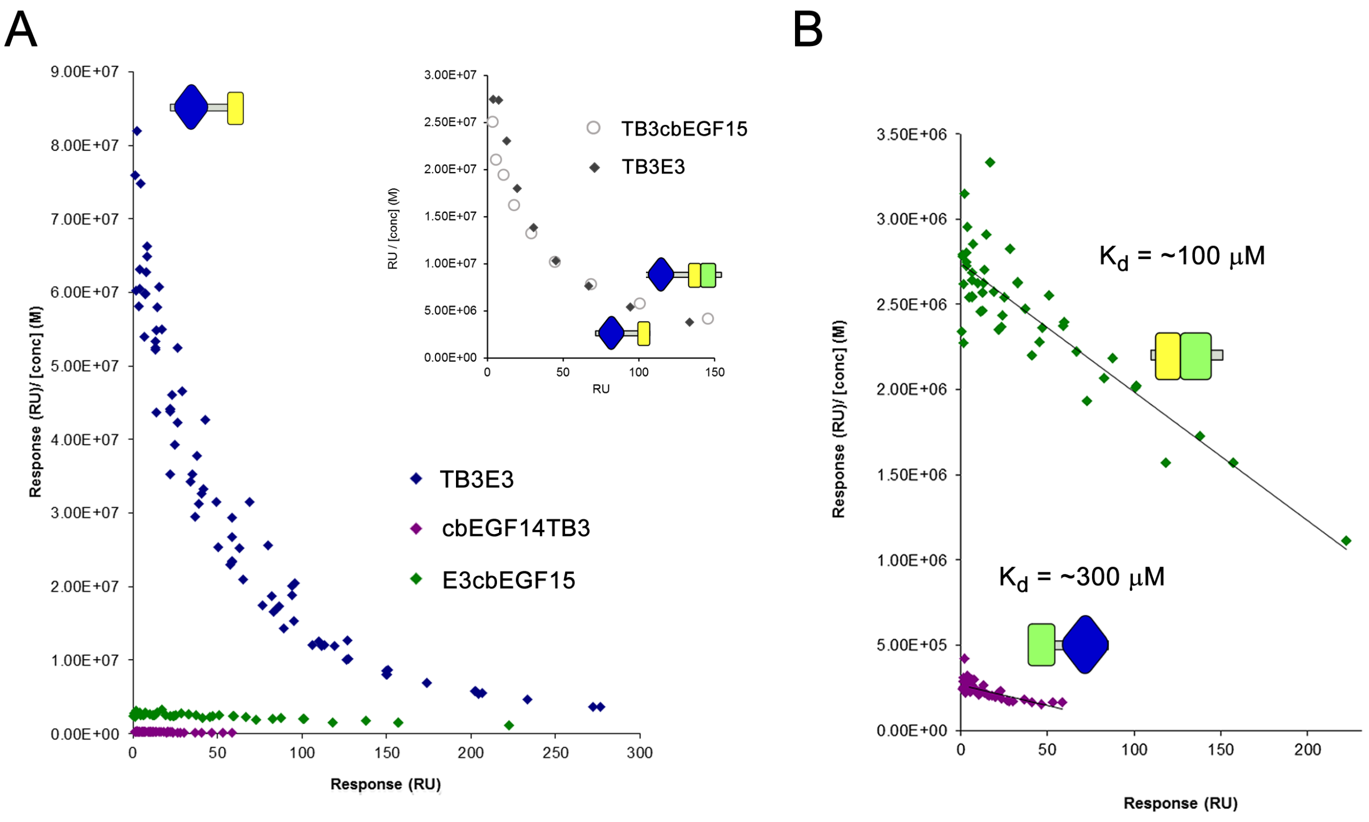

Figure S2. Related to Figure 1 and 2. Scatchard plots

Scatchard plots of single-cycle SPR data. In A) it can be seen that the interaction of $LTBP1^{TB3E3}$ with $FBN1^{E2cbEGF1}$ gives a non-linear Scatchard plot both with the single-cycle SPR method (main) and multi-cycle method (inset). This is consistent with complex kinetics of this interaction and makes determination of a single K_d impossible. Using the lowest concentration data from the single-cycle SPR, a K_d value in the range of 0.5-1μM can be estimated. In the multi-cycle data (shown in the inset) it is clear that $LTBP1^{TB3cbEGF15}$ and $LTBP1^{TB3E3}$ behave in a very similar manner in SPR, as they do in the plate assay (Figure 1C). B) The Scatchard plots of the more weakly binding LTBP1 constructs show more linear behaviour. K_d estimates are shown although saturation was not reached in these experiments.

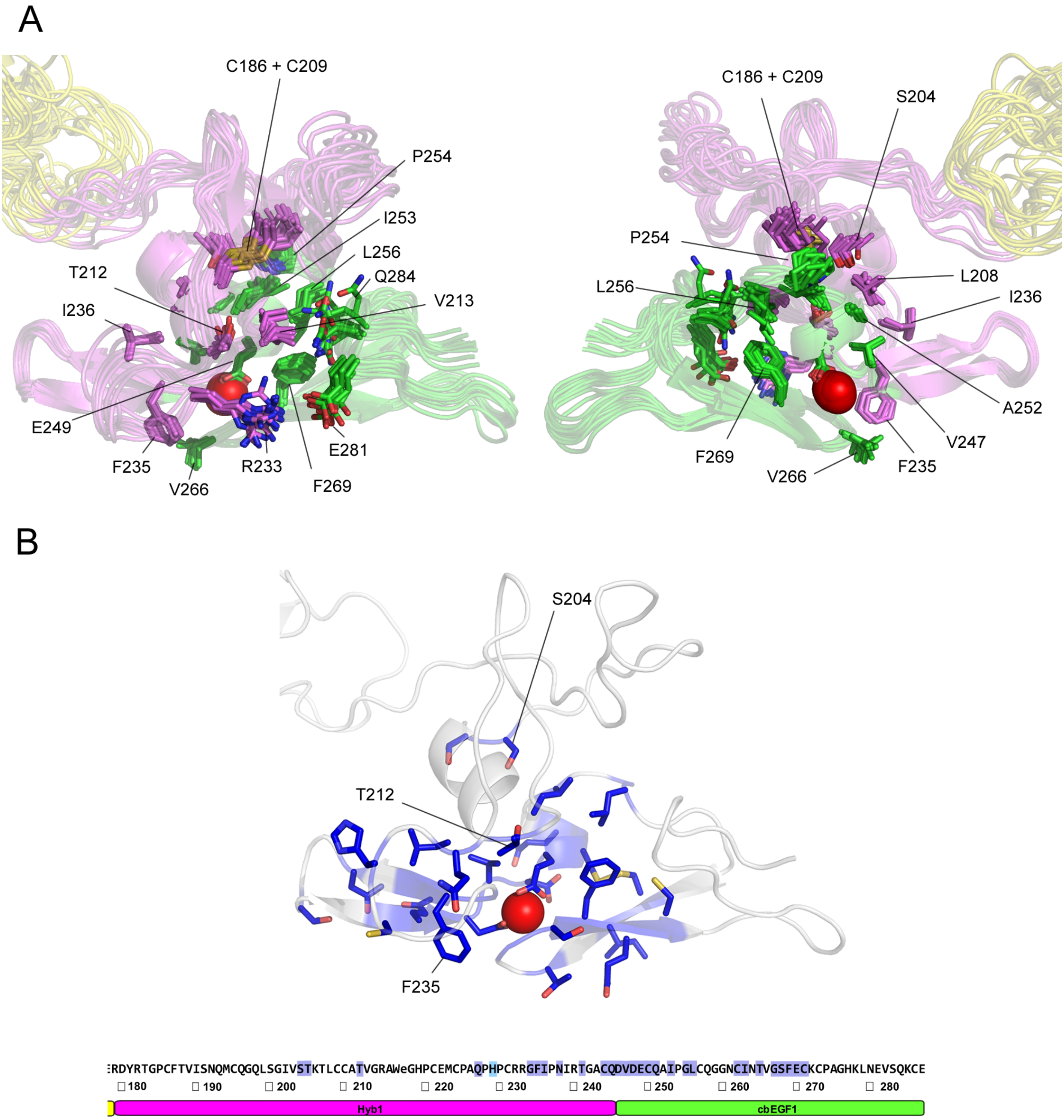

Figure S3. Related to Figure 6 and Table 1. Structure of the Hyb1-cbEGF1 inter-domain interface

(A) Two views of the interface between Hyb1 and cbEGF1 is defined by 74 unambiguous NOE restraints. A number of hydrophobic side chains define an extensive interface between Hyb1 (pink) and cbEGF1 (green), with interface side chains represented here as sticks. This interface surrounds the Ca^{2+}-binding site (red sphere) and may contribute to its high affinity. In many structures there is also a putative salt bridge between R233 in Hyb1 and E281 in cbEGF1. For clarity only 12 of the 20 structures in the ensemble are shown. (B) Residues in FBN1^{E2cbEGF1} with Ca^{2+}-sensitive NMR peaks are highlighted blue in the FBN1^{E2cbEGF1} sequence, and represented as blue sticks in the consensus structure.

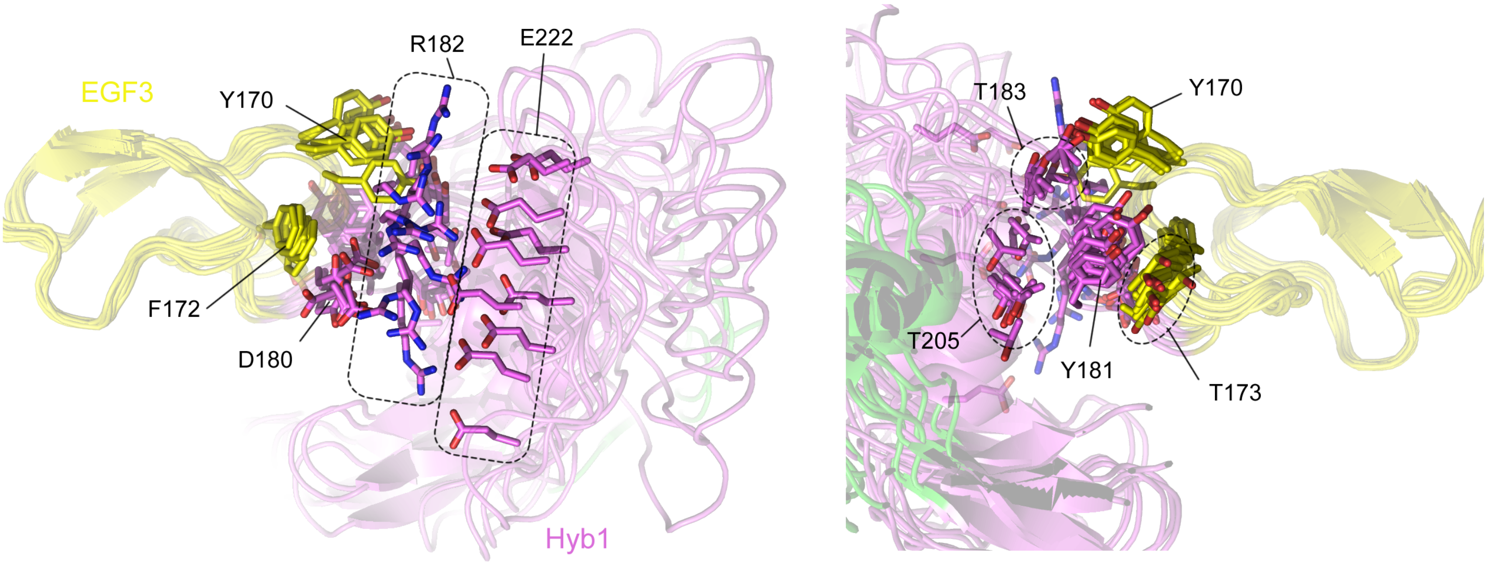

Figure S4. Related to Figure 6 and Table 1. Structure of the EGF3-Hyb1 inter-domain interface
Two views of the EGF3-Hyb1 inter-domain interface. Only a small number of residues define the EGF3-Hyb1 interface and this introduces some ambiguity into the relative orientation of EGF3 and Hyb1. However, the observed NOEs do define hydrophobic interactions between the aromatic side chains of Y170, F172, and Y181, and methyl groups of T173, T183 and T205. Furthermore in many structures R182 and E222 are close enough together that they could potentially form a salt bridge that could stabilise the inter-domain interface, although this salt bridge could still support a number of different domain orientations. The structures have been superimposed on EGF3 and the spread of orientations for Hyb1 can be seen. For clarity only 10 of the 20 structure ensemble are shown here.

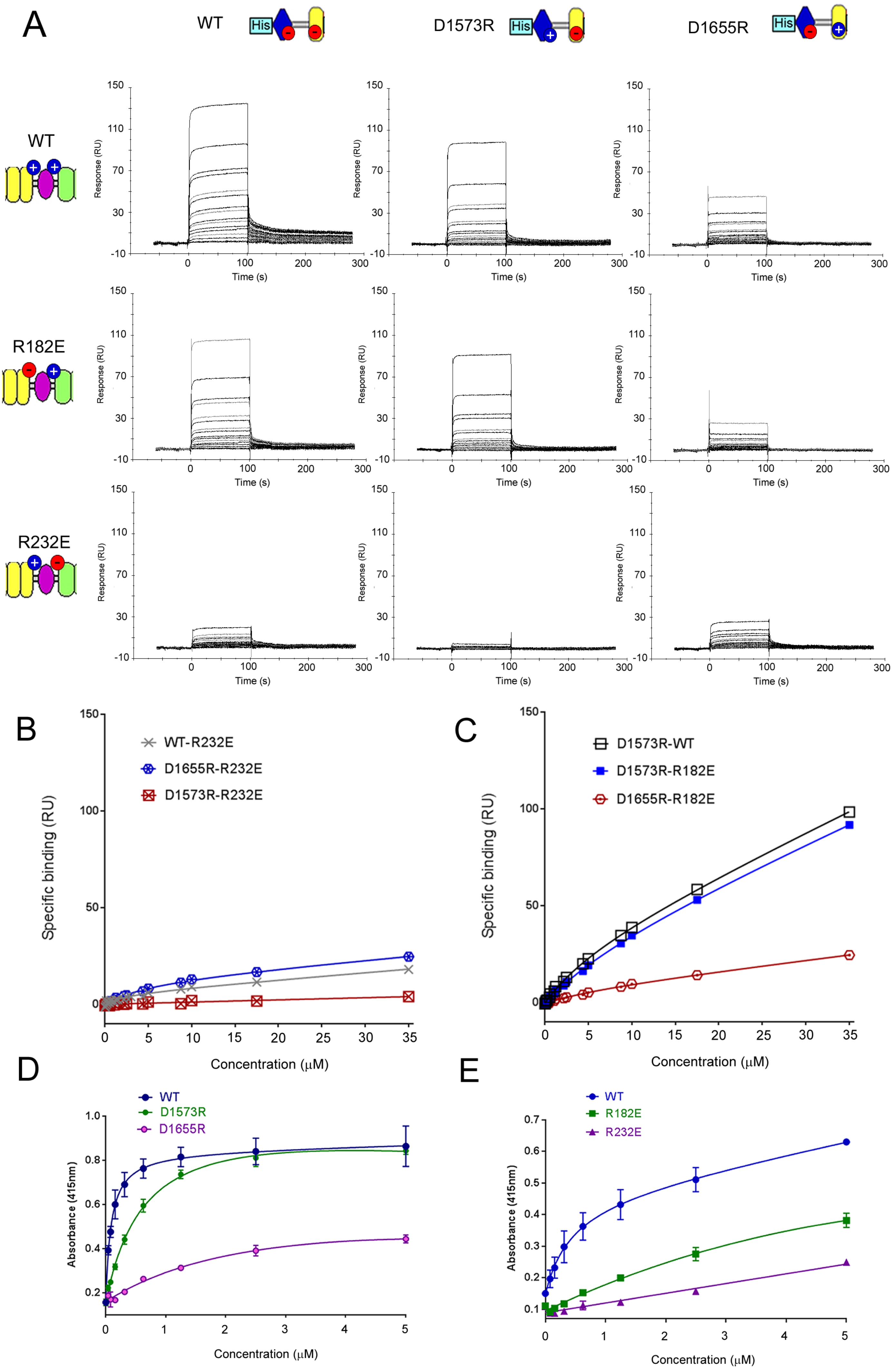

A
WT
D1573R
D1655R
His
WT
R182E
R232E
Response (RU)
Time (s)
B
WT-R232E
D1655R-R232E
D1573R-R232E
Specific binding (RU)
Concentration (μM)
C
D1573R-WT
D1573R-R182E
D1655R-R182E
Specific binding (RU)
Concentration (μM)
D
WT
D1573R
D1655R
Absorbance (415nm)
Concentration (μM)
E
WT
R182E
R232E
Absorbance (415nm)
Concentration (μM)

Figure S5. Related to Figure 7. SPR and Plate-based assays of LTBP1 and FBN1 charge reversal substitutions

(A) Blank subtracted multi-cycle SPR data with WT and charge-reversal $FBN1^{E2cbEGF1}$ fragments amine-coupled to the chip. $LTBP1^{TB3E3}$ fragments were injected as analytes at concentrations of 35, 17.5, 10, 8.75, 5, 4.38, 2.5, 2.18, 1.25, 1.09, 0.625, 0.547, 0.273, 0.137, 0.068, and 0.034 µM. (B) Plot of SPR responses from (A) for WT, D1655R $LTBP1^{TB3E3}$ and D1573R $LTBP1^{TB3E3}$ constructs binding $FBN1^{E2cbEGF1}$ with the R232E substitution. (C) Plot of SPR responses from (A) for D1573R and D1655R $LTBP1^{TB3E3}$ constructs binding WT and R182E $FBN1^{E2cbEGF1}$. The results in (B) show that the combination of D1655R LTBP1 and R232E FBN1 substitutions, which are residues involved in a putative salt bridge, gives no significant additional loss in binding compared to the single R232E substitution. This is consistent with these residues interacting with each other and not other residues in the FBN1/LTBP1 binding site; if these substitutions acted independently then an additive effect further reducing binding would be expected. The same is observed in (C) for the D1573R and R182E substitutions. In contrast to this, in (B) the combination of D1573R LTBP1 and R232E FBN1 substitutions, which are residues involved in two different putative salt bridges, gives an additional loss in binding compared to the single R232E substitution. This is consistent with these residues acting independently in two salt bridges giving an additive effect that further reduces binding. The same is observed in (C) for the D1655R and R182E substitutions. (D) Plate-binding assay data collected from a $FBN1^{E2cbEGF1}$ coated plate with two $LTBP1^{TB3E3}$ substitutions of interest, D1573R (green) and D1655R (magenta), compared to WT (blue). (E) Plate-binding assay data collected from wells coated with either WT $Fbn^{E2cbEGF1}$, R182E $Fbn^{E2cbEGF1}$ or R232E $Fbn^{E2cbEGF1}$. The data presented for each protein in (D) and (E) are from a single plate; three repeats of each protein concentration were carried out to determine experimental error (standard deviation). The plate-binding assay data in (D) and (E) confirm the SPR results shown in Figure 7 D and E.

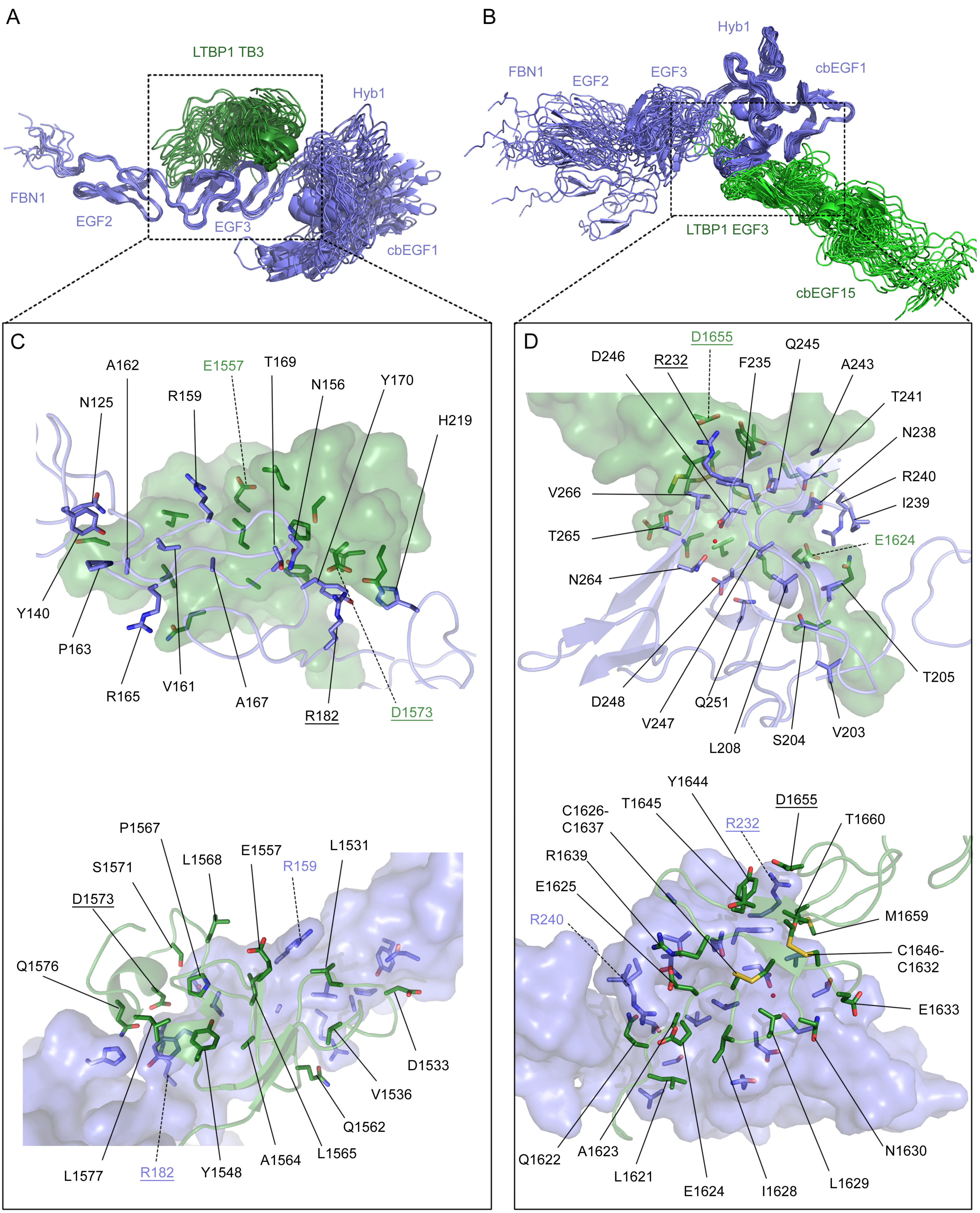

Figure S6. Related to Figure 8. Details of LTBP1-FBN1 interfaces identified by HADDOCK
(A) The top three best scoring HADDOCK clusters for the interaction of LTBP1^{TB3} and FBN1^{E2cbEGF1}

aligned by the EGF2-EGF3 domain pair of FBN1. (B) The top four best scoring HADDOCK clusters for the interaction of LTBP1^{E3cbEGF15} with FBN1^{E2cbEGF1} aligned by the Hyb1-cbEGF1 domain pair of FBN1. LTBP1 structures are coloured green and FBN1 coloured slate blue. (C) Residue side chains that form potential contacts between FBN1 EGF2-EGF3-Hyb1 and LTBP1 TB3. At the top FBN1 residues are highlighted and the surface of LTBP1 TB3 is shown in green, while on the bottom LTBP1 residues are highlighted and the surface of FBN1 is shown in slate blue. (D) Residue side chains that form potential contacts between FBN1 Hyb1-cbEGF1 and LTBP1 EGF3. On the top FBN1 residues are highlighted and the surface of LTBP1 EGF3 is shown in green, while on the bottom LTBP1 residues are highlighted and the surface of FBN1 is shown in slate blue. Residues identified as involved in salt bridges are underlined.

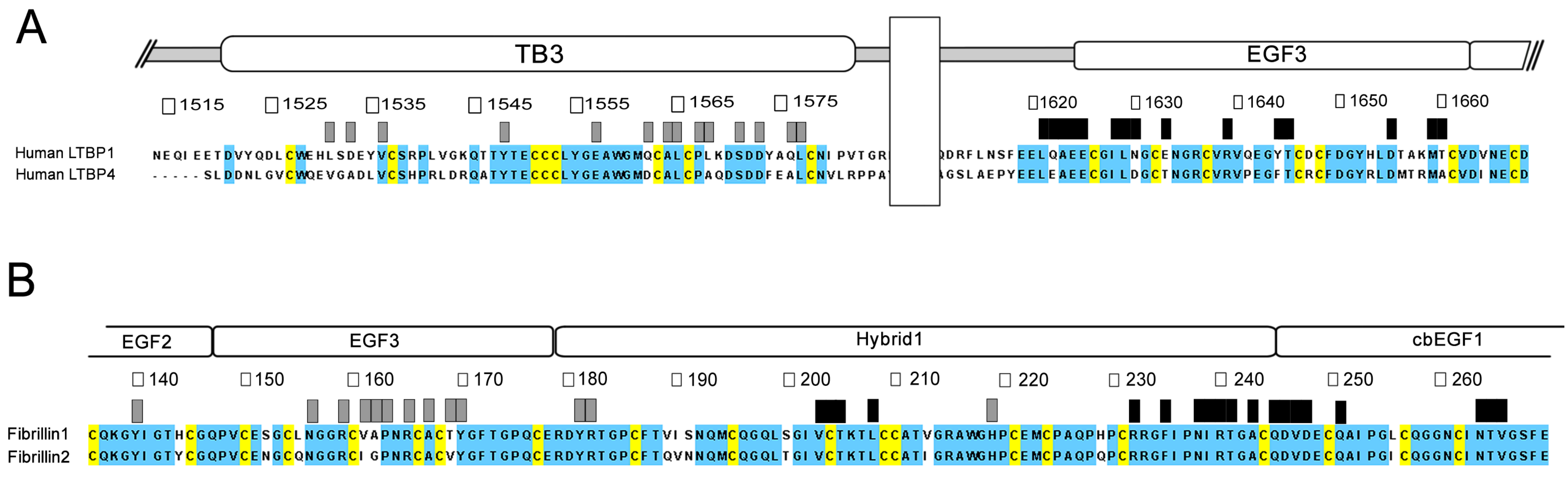

Figure S7. Related to Figure 8– Alignments of FBN and LTBP
(A) C-terminal region of LTBP1 implicated in FBN1 binding aligned with the C-terminal region of LTBP4. Grey boxes above the alignment indicate residues highlighted in Figure S6C as interacting with FBN1 EGF2-3 and Hyb1, while black boxes indicate residues highlighted in Figure S6D as interacting with FBN1 Hyb1-cbEGF1. Residues that are present in both sequences are highlighted blue, with the exception of cysteines which are highlighted yellow. A white box is shown where the inter-domain linker has been truncated. Comparison of the sequences of human LTBP1 and LTBP4 shows amino acid substitutions in both binding sites. The stronger FBN1 Hyb1-cbEGF1/LTBP1 EGF3 interface has substitutions Q1622E, N1630D, E1633T and T1660A while the weaker FBN1 EGF3-Hyb1/LTBP1 TB3 interface was substitutions D1533A, Q1562D, L1568A and Q1576A. These substitutions alter the electrostatic properties of the binding sites in the LTBPs and could substantially change the mode of interaction with FBN1. (B) N-terminal region of FBN1 implicated in LTBP1 binding aligned with the N-terminal region of FBN2. Grey boxes above the alignment indicate residues highlighted in Figure S6C as interacting with LTBP1 TB3, while black boxes indicate residues highlighted in Figure S6D as interacting with LTBP1 EGF3. Residues that are present in both sequences are highlighted blue, with the exception of cysteines which are highlighted yellow. Comparison of the sequences of human FBN1 and FBN2 shows complete conservation of residues involved in the strong FBN Hyb1-cbEGF1/LTBP1 EGF3 interface whereas three substitutions (V161I, A162G and T169V) are observed in the weaker FBN EGF3-Hyb1/LTBP1 TB3 interface. A reduction of binding affinity in the latter site could explain the observed differences in binding for FBN1/FBN2. N164, associated with dominant ectopia lentis via the N164S substitution (Comeglio et al., 2002), is conserved in FBN1/FBN2.

Supplemental Table S1. Related to Figure 8. – Restraints for final round of HADDOCK docking

	$LTBP1^{TB3}$ - $FBN1^{E2cbEGF1}$ docking	$LTBP1^{E3cbEGF15}$ – $FBN1^{E2cbEGF1}$ docking
FBN1 active residues	159,162,167,168,170,177,182	204,227,232,235,239,241,242,243,244,245,246, 249
FBN1 passive residues	125,126,128,138,140,145,147,148,149, 154,156,157,158,160,161,163,164,165, 166,169,171,172,173,174,175,176,178, 179,180,181,183,184,185	203,205,206,207,208,210,211,212,217,224,225, 226,228,229,230,231,233,234,236,237,238,240, 247,248,251,252,253,254,256,264,266,267,269
FBN1 fully flexible regions	111-118, 178-185, 236-243	111-118, 178-185, 236-243
LTBP1 active residues	1523,1562,1563,1569,1571,1574,1577, 1580	1622,1623,1625,1626,1628,1630,1631,1632, 1636,1638,1639,1641,1642,1643,1644,1646, 1647,1648,1656,1655
LTBP1 passive residues	1529,1539,1548,1549,1550,1561,1570, 1524,1525,1526,1528,1531,1532,1533, 1534,1535,1536,1537,1546,1547,1553, 1554,1559,1560,1564,1565,1566,1567, 1568,1572,1573,1575,1576,1578,1579, 1581,1582	1627,1628,1633,1634,1635,1637,1640,1644, 1645,1649,1650,1651,1652,1653,1654,1657, 1658,1659,1660,1661,1662,1620,1621,1622, 1623,1624,1625,1626,1630,1631,1632,1636, 1638,1639,1641,1642,1643,1646,1647,1648, 1656
LTBP1 fully flexible residues	1521- 1527, 1579-1582	1617- 1625,1638-1645, 1653-1660
Other restraints	ASSIGN (RESID 182 and NAME CZ and SEGID A) (RESID 1573 and NAME CG and SEGID C) 0.0 0.0 6.0	ASSIGN (RESID 232 and NAME CZ and SEGID A) (RESID 1655 and NAME CG and SEGID B) 0.0 0.0 6.0
HADDOCK results for highest scoring clusters		
HADDOCK score	-102.1 +/- 5.8	-131.1 +/- 15.5
Cluster size	15	7
RMSD from the overall lowest-energy structure	5.1 +/- 0.3	3.6 +/- 2.2
Van der Waals energy	-56.3 +/- 3.9	-81.6 +/- 4.9
Electrostatic energy	-162.3 +/- 16.6	-220.3 +/- 52.5
Desolvation energy	-28.5 +/- 4.2	-26.9 +/- 3.9
Restraints violation energy	152.5 +/- 22.24	214.6 +/- 12.64
Buried Surface Area	1707.7 +/- 55.4	2221.3 +/- 131.6
Z-Score	-1.3	-1.5

Supplemental Table S2. Related to Figure 7. – Primers for site-directed mutagenesis

Construct	Primer 5’ > 3’
**LTBP1:**	
LTBP1^{TB3E3} D1521K fwd	CAAATAGAAGAAACTAAGGTCTACCAAGATTTGTGCTGGGAAC
LTBP1^{TB3E3} D1521K rev	GTTCCCAGCACAAATCTTGGTAGACCTTAGTTTCTTCTATTTG
LTBP1^{TB3E3} D1573R fwd	CCCTGAAGGATTCAGATCGCTATGCTCAGCTGTGTAAC
LTBP1^{TB3E3} D1573R rev	GTTACACAGCTGAGCATAGCGATCTGAATCCTTCAGGG
LTBP1^{TB3E3} E1625R fwd	GTTACAGGCTGAGCGATGCGGCATCCTCAATGG
LTBP1^{TB3E3} E1625R rev	CCATTGAGGATGCCGCATCGCTCAGCCTGTAAC
LTBP1^{TB3E3} E1642R fwd	CTGTGTGAGGGTCCAGCGAGGTTACACCTGCG
LTBP1^{TB3E3} E1642R rev	CGCAGGTGTAACCTCGCTGGACCCTCACACAG
LTBP1^{TB3E3} D1655R fwd	GGGTATCACTTGCGTACGGCCAAGATGACCTGTG
LTBP1^{TB3E3} D1655R rev	CACAGGTCATCTTGGCCGTACGCAAGTGATACCC
**Fibrillin1**	
FBN1^{E2cbEGF1} K138D fwd	CACTGTCTATGCCAGGATGGATACATAGGGACTCACTG
FBN1^{E2cbEGF1} K138D rev	CAGTGAGTCCCTATGTATCCATCCTGGCATAGACAGTG
FBN1^{E2cbEGF1} R182E fwd	CCAGTGTGAAAGAGATTACGAGACAGGCCCATGTTTTAC
FBN1^{E2cbEGF1} R182E rev	GTAAAACATGGGCCTGTCTCGTAATCTCTTTCACACTGG
FBN1^{E2cbEGF1} R232E fwd	CCAGCCTCACCCCTGCGAACGTGGCTTCATTCC
FBN1^{E2cbEGF1} R232E rev	GGAATGAAGCCACGTTCGCAGGGGTGAGGCTGG
FBN1^{E2cbEGF1} C204S fwd	CAACTCAGCGGGATTGTCTCCACAAAAACGCTCTGCTGTG
FBN1^{E2cbEGF1} C204S rev	CACAGCAGAGCGTTTTTGTGGAGACAATCCCGCTGAGTT
