# Supplementary material for: The N-Terminal Region of Fibrillin-1 Mediates a Bipartite Interaction with LTBP1
Source: Structure. 2017 Aug 1;25(8):1208–1221.e5. doi: 10.1016/j.str.2017.06.003 (PMC5548924; doi:10.1016/j.str.2017.06.003)
Supplement: Document S2. Article plus Supplemental Information [file mmc3.pdf]

Structure

Article

The N-Terminal Region of Fibrillin-1 Mediates a Bipartite Interaction with LTBP1

Graphical Abstract

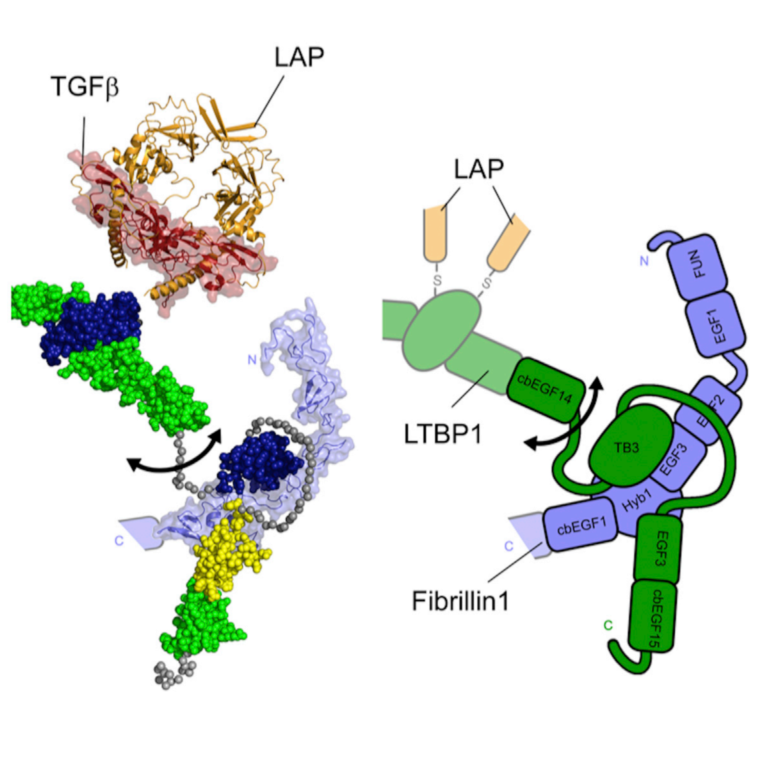

Authors

Ian B. Robertson, Hans F. Dias, Isabelle H. Osuch, Edward D. Lowe, Sacha A. Jensen, Christina Redfield, Penny A. Handford

Correspondence

christina.redfield@bioch.ox.ac.uk (C.R.), penny.handford@bioch.ox.ac.uk (P.A.H.)

In Brief

Improving our knowledge of TGF-β regulation by matrix biomechanics is vital for understanding the biology of this enigmatic growth factor. Robertson et al. present a bipartite model for the structure of the fibrillin-1-LTBP1 interaction that functions as a holdfast for TGF-β in the matrix.

Highlights

- The structure of the FBN1 N-terminal region shows a near-linear domain organization
- LTBP1 binds to FBN1 via a bipartite mode of interaction involving two discreet sites
- This allows LTBP1 to connect 10–12 nm FBN1 microfibrils to other ECM networks
- This may facilitate force-induced/traction-based activation of TGF-β via integrins

Data Resources

5MS9
19078
18848
19322

Robertson et al., 2017, Structure *25*, 1208–1221
August 1, 2017 © 2017 The Authors. Published by Elsevier Ltd.
http://dx.doi.org/10.1016/j.str.2017.06.003

Structure
Article

The N-Terminal Region of Fibrillin-1 Mediates a Bipartite Interaction with LTBP1

Ian B. Robertson,[1] Hans F. Dias,[1] Isabelle H. Osuch,[1] Edward D. Lowe,[1] Sacha A. Jensen,[1] Christina Redfield,[1,*] and Penny A. Handford[1,2,*]
[1]Department of Biochemistry, University of Oxford, South Parks Road, Oxford OX1 3QU, UK
[2]Lead Contact
*Correspondence: christina.redfield@bioch.ox.ac.uk (C.R.), penny.handford@bioch.ox.ac.uk (P.A.H.)
http://dx.doi.org/10.1016/j.str.2017.06.003

SUMMARY

***Fibrillin-1* (*FBN1*) mutations associated with Marfan syndrome lead to an increase in transforming growth factor β (TGF-β) activation in connective tissues resulting in pathogenic changes including aortic dilatation and dissection. Since FBN1 binds latent TGF-β binding proteins (LTBPs), the major reservoir of TGF-β in the extracellular matrix (ECM), we investigated the structural basis for the FBN1/LTBP1 interaction. We present the structure of a four-domain FBN1 fragment, EGF2-EGF3-Hyb1-cbEGF1 ($FBN1^{E2cbEGF1}$), which reveals a near-linear domain organization. Binding studies demonstrate a bipartite interaction between a C-terminal LTBP1 fragment and $FBN1^{E2cbEGF1}$, which lies adjacent to the latency-associated propeptide (LAP)/TGF-β binding site of LTBP1. Modeling of the binding interface suggests that, rather than interacting along the longitudinal axis, LTBP1 anchors itself to FBN1 using two independent epitopes. As part of this mechanism, a flexible pivot adjacent to the FBN1/LTBP1 binding site allows LTBP1 to make contacts with different ECM networks while presumably facilitating a force-induced/traction-based TGF-β activation mechanism.**

INTRODUCTION

The fibrillin/latent transforming growth factor β (TGF-β) binding protein (LTBP) family of extracellular matrix (ECM) proteins are calcium-binding glycoproteins whose domain organization is dominated by multiple tandem repeats of calcium-binding epidermal growth factor-like (cbEGF) and TGF-β binding protein-like (TB) domains (Figure 1A) (Robertson et al., 2011). Fibrillin (FBN) is an evolutionarily ancient protein which plays an important structural role in connective tissues through its higher-order association into 10–12 nm microfibrils (Jensen et al., 2012; Keene et al., 1991). The LTBPs are responsible for sequestering the small latent complex, comprising latency-associated propeptide (LAP) and TGF-β in the matrix (Saharinen and Keski-Oja, 2000). Recent research suggests that FBN also has an important regulatory role in development and homeostasis by transmitting diverse information about the extracellular environment to cells (Sengle and Sakai, 2015). Such signal transduction might occur through direct interactions between FBN and cell-surface integrin receptors (Pfaff et al., 1996; Sakamoto et al., 1996; Zeyer and Reinhardt, 2015) and/or through its direct or indirect sequestration of growth factors including various TGF-β superfamily members (Robertson and Rifkin, 2016).

Studies of genetic diseases and mouse models have demonstrated important physiological connections between FBN1 and TGF-β signaling *in vivo*. Marfan syndrome, caused by loss-of-function *FBN1* mutations, can result in aortic dilatation and dissection, as well as pathological changes to the skeleton and the eye (Doyle et al., 2012). Inhibition of TGF-β, either directly using neutralizing antibodies or indirectly using drugs such as Losartan, has demonstrated reversal of pathological changes in the aorta, lung, and skeleton in *FBN1* mouse models, suggesting that FBN1 levels in the ECM influence TGF-β activity (Cohn et al., 2007; Habashi et al., 2011; Lima et al., 2010; Neptune et al., 2003). Subsequent studies have shown that both integrin levels and changes in mechano-sensation by the angiotensin receptor can further modulate conditional *FBN* knockout mouse phenotypes indicating a complex cell/matrix interplay (Cook et al., 2014, 2015).

Cell biology studies have shown co-localization of FBN microfibrils with LTBPs in skin (Raghunath et al., 1998), bone (Dallas et al., 1995), and tissue culture (Dallas et al., 2000; Taipale et al., 1996). The LTBP isoforms LTBP1, 3, and 4 all covalently bind the small latent complex (comprising LAP and TGF-β) (Robertson et al., 2015; Saharinen and Keski-Oja, 2000). Subsequent biochemical studies have shown that there are interactions between eukaryotically expressed recombinant fragments of the N-terminal region of FBN1 and the C-terminal region of several of the LTBPs (Isogai et al., 2003; Massam-Wu et al., 2010; Ono et al., 2009). These studies identified the EGF2/EGF3 and Hybrid1 (Hyb1) domains of FBN1 and the TB3-EGF3-cbEGF15 region of LTBP1 as being important for binding. Collectively, mouse models, cell biology, and biochemical data place FBN, LTBP, and TGF-β at the center of a complex mechano-sensory network within connective tissue, the structural basis of which is unknown.

Here the structural basis for the interaction between FBN1 and LTBP1 has been investigated utilizing biophysical and biochemical techniques. The solution structure of a four-domain LTBP1-binding FBN1 fragment, EGF2-EGF3-Hyb1-cbEGF1, based on nuclear magnetic resonance (NMR) and small-angle X-ray scattering (SAXS) data, reveals a near-linear arrangement of domains, completing the structure of the N terminus of FBN1. Detailed dissection of the binding interface and subsequent modeling of the LTBP1/FBN1 complex indicates that LTBP1

 © 2017 The Authors. Published by Elsevier Ltd.
This is an open access article under the CC BY license (http://creativecommons.org/licenses/by/4.0/).

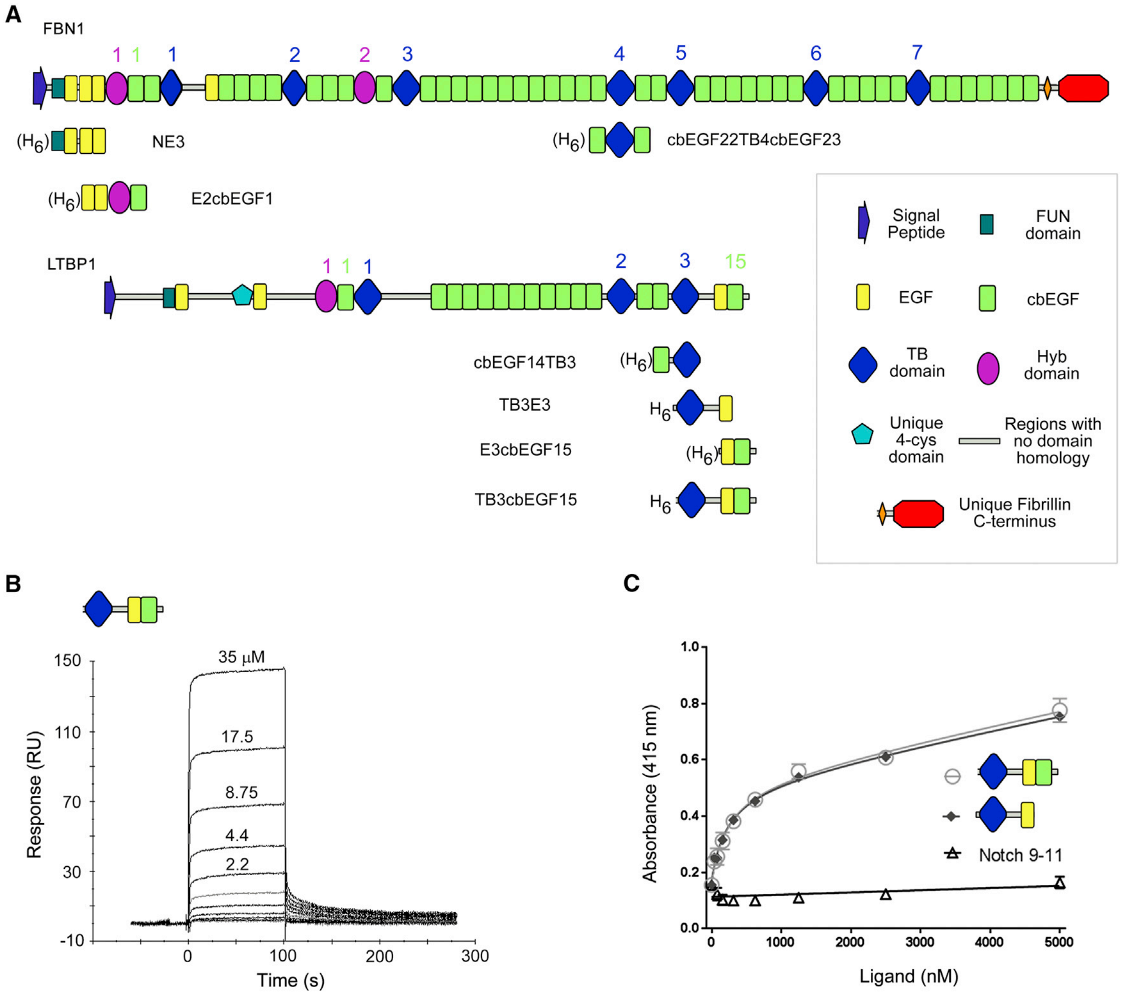

Figure 1. Overlapping Protein Constructs Used to Study the Interaction between FBN1 and LTBP1

(A) The FBN1 and LTBP1 constructs used in this study are shown in the context of the domain organization of the full-length proteins. "H_6" in front of the constructs denotes that the protein is expressed with a hexa-His tag and the brackets indicate that it can be cleaved by factor Xa without degradation of the protein. Domain numbers for the TB and Hyb domains and for selected cbEGF domains are indicated and are color coded according to domain type.

(B) Multi-cycle SPR data showing the concentration-dependent interaction of the LTBP1^{TB3cbEGF15} construct with FBN1^{E2cbEGF1} amine-coupled to the surface of the sensor chip.

(C) Plate-binding assay showing the interaction of the His-tagged three-domain LTBP1^{TB3cbEGF15} (○) or two-domain LTBP1^{TB3E3} (◆) constructs with a four-domain FBN1^{E2cbEGF1} construct immobilized on the plate surface. No binding was seen with a control three-domain His-tagged Notch EGF9-11 construct (△). The data presented are from a single plate; three repeats of each protein concentration were carried out to determine experimental error (SD). See also Figure S1.

binds FBN1 via its C-terminal TB3 and EGF3 domains in a bipartite interaction involving two different faces of the FBN1 molecule. This localized interaction ensures tight binding to FBN1 while allowing the N-terminal region of LTBP1 to engage with other ECM networks. This may facilitate regulated TGF-β activation by traction-based mechanisms involving integrins, and suggests that FBN1 deficiency precludes optimal localization of LTBP in the ECM for regulated TGF-β activation.

RESULTS AND DISCUSSION

Binding Studies Identify a Specific Interaction between LTBP1 and FBN1

Previous data utilizing eukaryotically expressed fragments identified a specific interaction between the C-terminal region of LTBP1 and the N-terminal region of FBN1 (Isogai et al., 2003; Ono et al., 2009). Here, overlapping protein fragments derived from the N terminus of FBN1 and the C terminus of LTBP1 have been bacterially expressed and refolded *in vitro* (Figures 1A and S1), as described previously (Robertson et al., 2013a, 2013b; Yadin et al., 2012), to probe their interaction at the molecular level and to determine a model of the interaction complex.

We observed a specific interaction between a three-domain C-terminal LTBP1^{TB3cbEGF15} construct and a four-domain FBN1^{E2cbEGF1} construct using both surface plasmon resonance (SPR) and a plate-based binding assay (Figures 1B and 1C). This confirms the interaction reported previously using eukaryotically expressed material (Isogai et al., 2003; Ono et al., 2009), and demonstrates that the core recognition elements are contained in the amino acid sequence of the proteins.

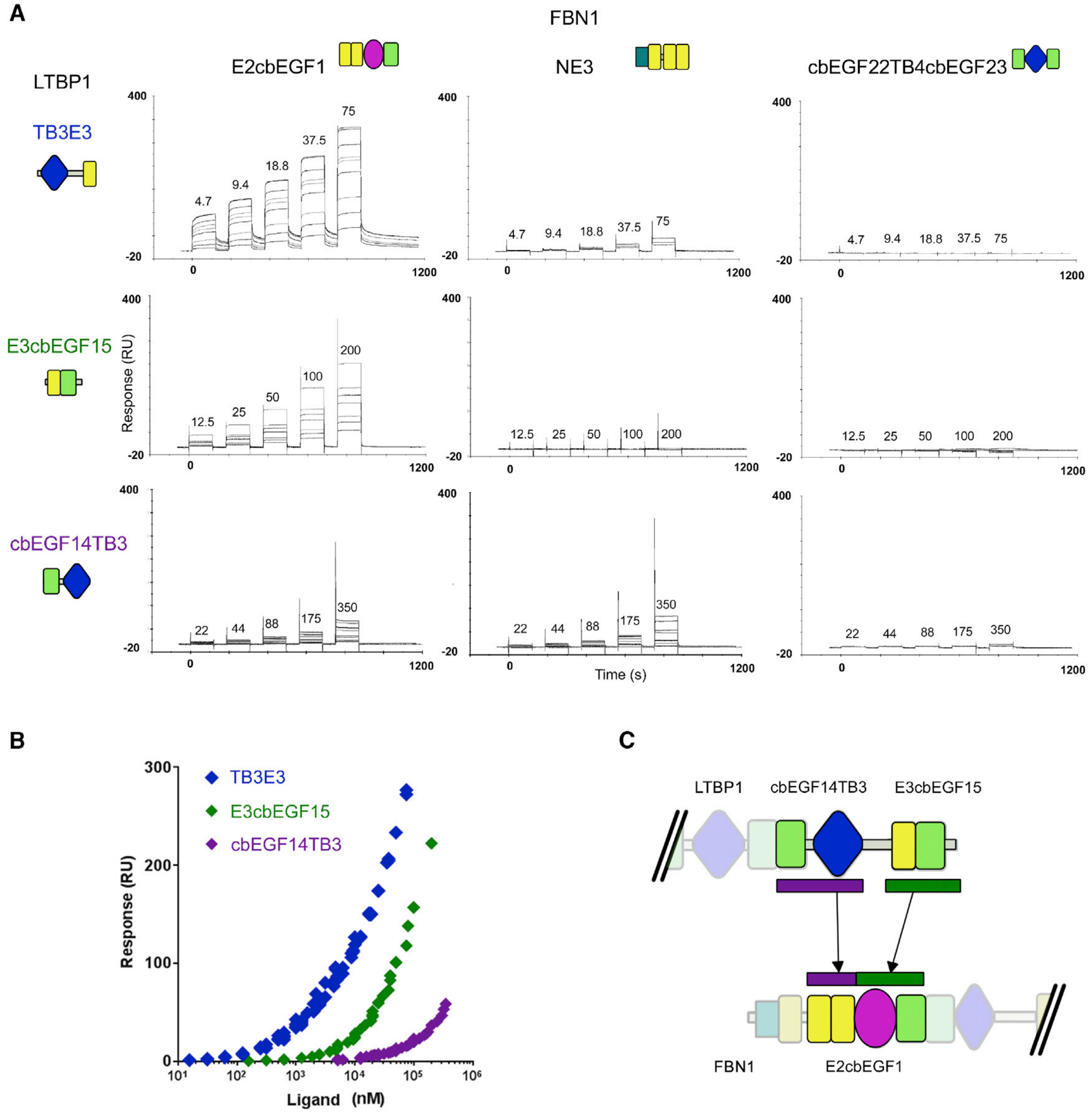

Figure 2. Interactions of FBN1 and LTBP1 Domain Constructs Measured by SPR

(A) Single-cycle SPR data showing the interaction of three overlapping two-domain LTBP1 constructs (analytes) with three SPR flow cells coated with different FBN1 constructs. LTBP1 concentrations are shown in μM above the relevant part of the sensorgram for the highest concentration single-cycle experiment. The FBN1 construct containing the cbEGF22-TB4-cbEGF23 domains was used as a control.

(B) Plot of SPR responses from three different LTBP1 constructs binding to FBN1^{E2cbEGF1}; this demonstrates that for the interaction with FBN1^{E2cbEGF1} K_d(TB3E3) < K_d(E3cbEGF15) < K_d(cbEGF14TB3). Concentrations are shown on a logarithmic scale to accommodate the weak binding of LTBP1^{cbEGF14TB3}.

(C) Schematic representation of the interaction model suggested by the SPR data. See also Figures S1 and S2.

We compared the binding of the three-domain LTBP1^{TB3cbEGF15} and two-domain LTBP1^{TB3E3} constructs to the four-domain FBN1^{E2cbEGF1} construct using the plate-based assay (Figure 1C). The binding responses of the two LTBP1 fragments to FBN1 are the same, suggesting that the cbEGF15 domain of LTBP1 does not contribute to the interaction with FBN1.

To dissect the interaction further using SPR, analytes LTBP1^{TB3E3}, LTBP1^{E3cbEGF15}, and LTBP1^{cbEGF14TB3}, each containing a pair of domains, were flowed over immobilized FBN1^{E2cbEGF1} (Figure 2A). The largest response was observed for LTBP1^{TB3E3} with moderate binding for LTBP1^{E3cbEGF15} and even weaker binding for LTBP1^{cbEGF14TB3} (Figure 2B). The high-affinity binding observed for LTBP1^{TB3E3} suggests that the TB3 and EGF3 domains, rather than the flanking cbEGF14 and cbEGF15 domains, are important for maximal binding. The observation of weak binding for both the LTBP1^{cbEGF14TB3} and LTBP1^{E3cbEGF15} constructs, which do not have any domains in common, suggests that more than one domain from LTBP1 interacts with FBN1. The relative binding strengths of the LTBP1 fragments also suggest that the EGF3 domain of LTBP1 makes a more significant contribution to the interaction with FBN1 than the TB3 domain of LTBP1.

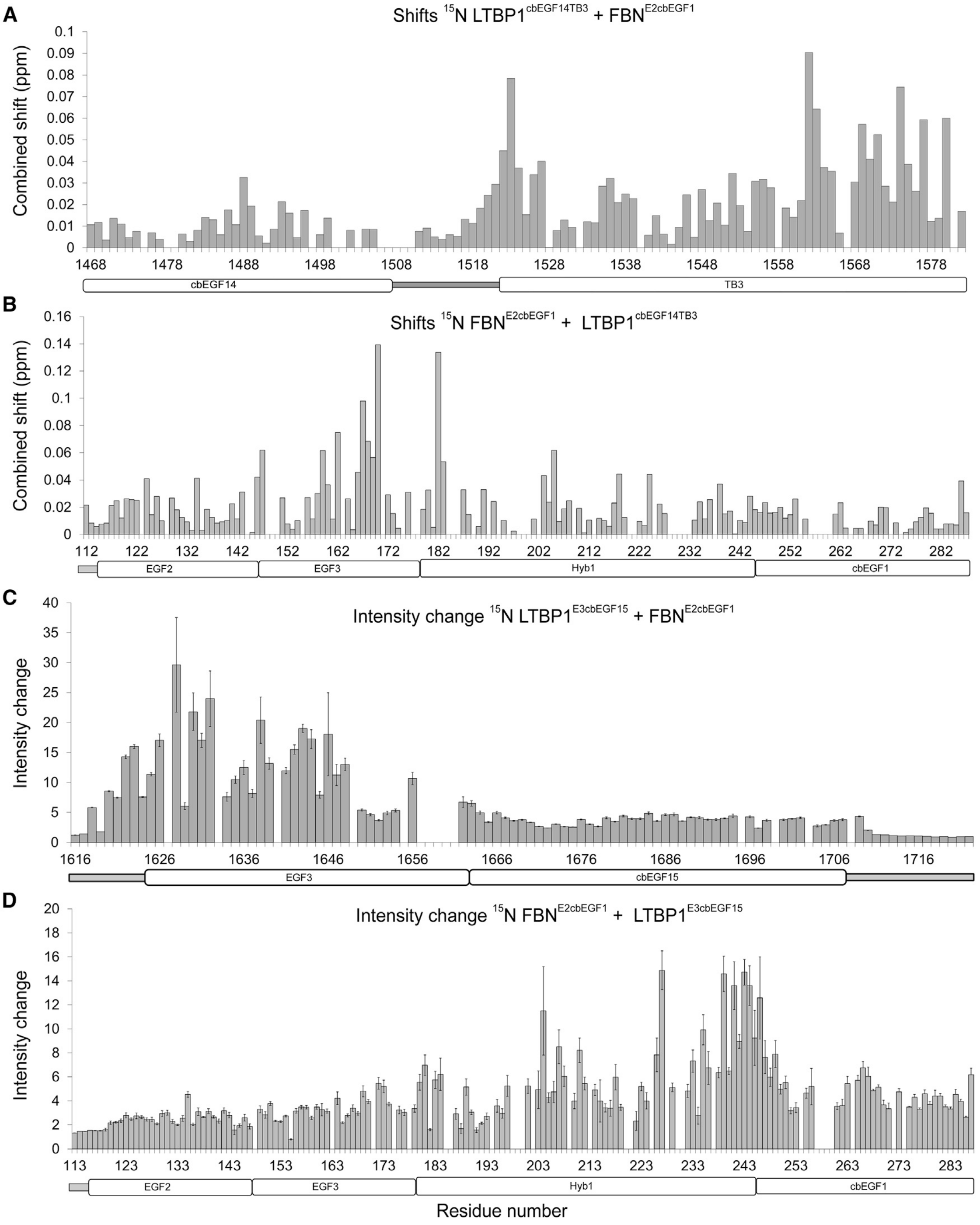

Figure 3. HSQC Titration Data Highlights Multiple Binding Sites in Both FBN1 and LTBP1

Titrations were carried out by sequential addition of lyophilized unlabeled protein to ^{15}N-labeled protein samples and monitored using ^{1}H-^{15}N HSQC spectra.

(A and B) Peaks were observed to shift in titrations of (A) ^{15}N-LTBP1^{cbEGF14TB3} with FBN1^{E2cbEGF1} added, or of (B) ^{15}N-FBN1^{E2cbEGF1} with LTBP1^{cbEGF14TB3} added. The combined ^{1}H^{N} and ^{15}N chemical shift change is plotted as a function of protein sequence.

(C and D) Peak broadening was observed in titrations of (C) ^{15}N-LTBP1^{E3cbEGF15} with FBN1^{E2cbEGF1} added or of (D) ^{15}N-FBN1^{E2cbEGF1} with LTBP1^{E3cbEGF15} added. Peak intensity changes, measured as the ratio of peak intensity in the absence of ligand to that in the presence of ligand, are plotted as a function of protein sequence. Error bars are determined from the effect of background noise on peak height (SD). Gaps in the plots occur for residues with unassigned or very weak peaks in the HSQC or for prolines.

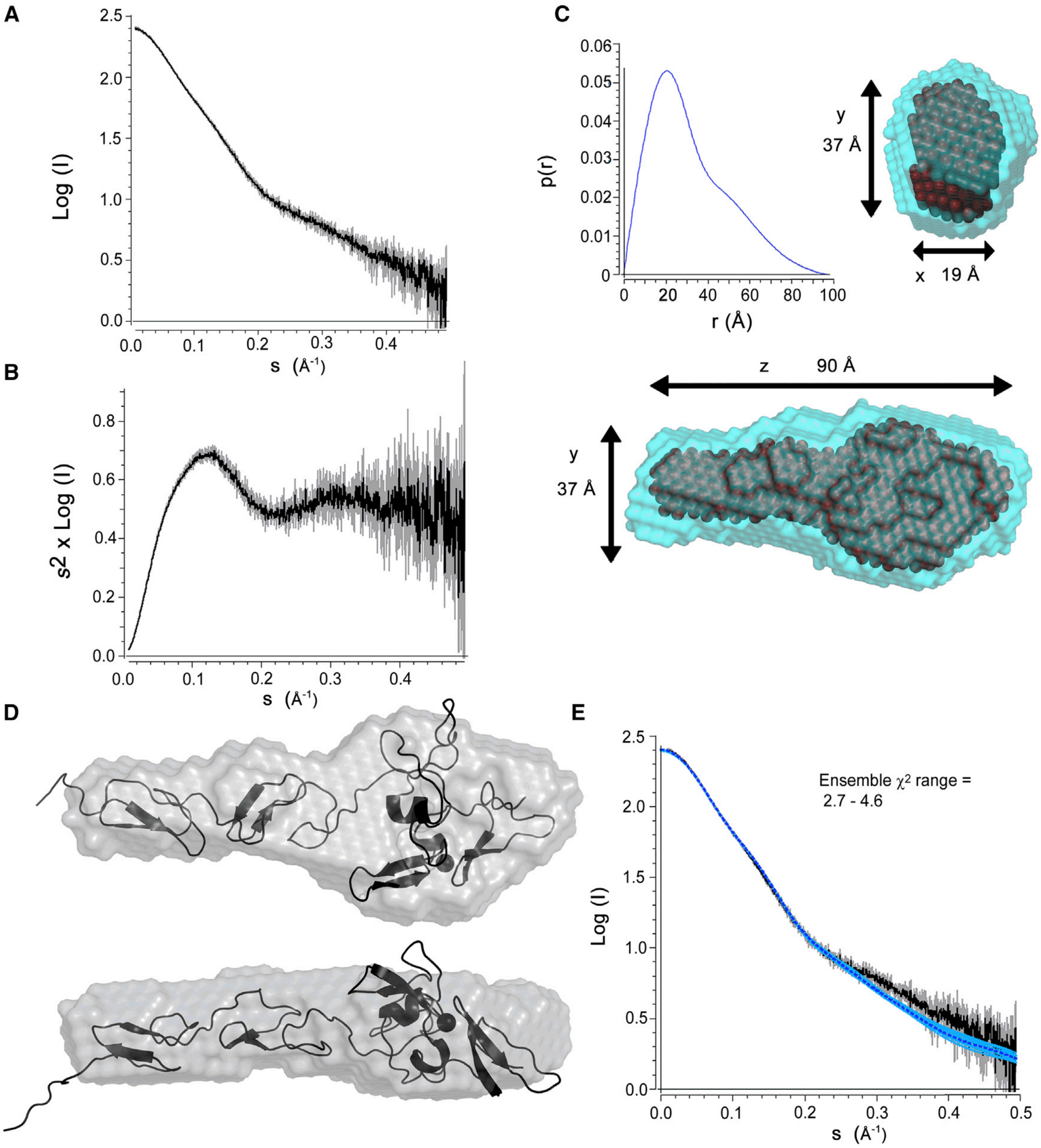

Figure 4. SAXS Data for FBN1^{E2cbEGF1} Reveals a Linear Shape

(A) Scaled, merged, and averaged X-ray scattering curves collected with purified FBN1^{E2cbEGF1} at concentrations of 11.55, 5.78, 2.89, and 1.44 mg/mL. Analysis of these data confirm that the protein behaves as a monomer in solution.

(B) Kratky plot of scaled, merged, and averaged SAXS data showing a peak falling to a plateau; this behavior is characteristic of a folded and relatively rigid protein.

(C) P(r) distribution and *ab initio* modeling of particle shape using DAMMIF. The blue transparent surface represents the shape produced from averaging the 20 independently generated *ab initio* structures with DAMAVER, and the darker spheres within this envelope represent the core shared particle shape calculated by the DAMFILT algorithm. Fitting this DAMFILT particle shape to the scattering data gave a χ^2 value of 0.7240.

(D) Comparison of a selected model (black cartoon) from the NMR structural ensemble with the envelope produced by DAMFILT *ab initio* modeling (gray surface).

(E) Fitting of the 20 structures in the NMR ensemble to the SAXS data. The SAXS data and error bars are shown in black and gray, respectively. The fits of the NMR structures are shown in blue with a dark blue dashed line showing the fit of model 1 of the NMR ensemble.

The error bars in (A), (B), and (E) are derived from the SCATTER software package using the data collected at four proteins concentrations.

The two-domain LTBP1 constructs were also flowed over a smaller immobilized FBN1 fragment, FBN1^{NE3}, which shares the EGF2-EGF3 domains with FBN1^{E2cbEGF1} (Figures 1A and 2A). The substantially weaker interaction observed for LTBP1^{TB3E3} with FBN1^{NE3} than with FBN1^{E2cbEGF1} suggests that the Hyb1 and cbEGF1 domains of FBN1 contain the major

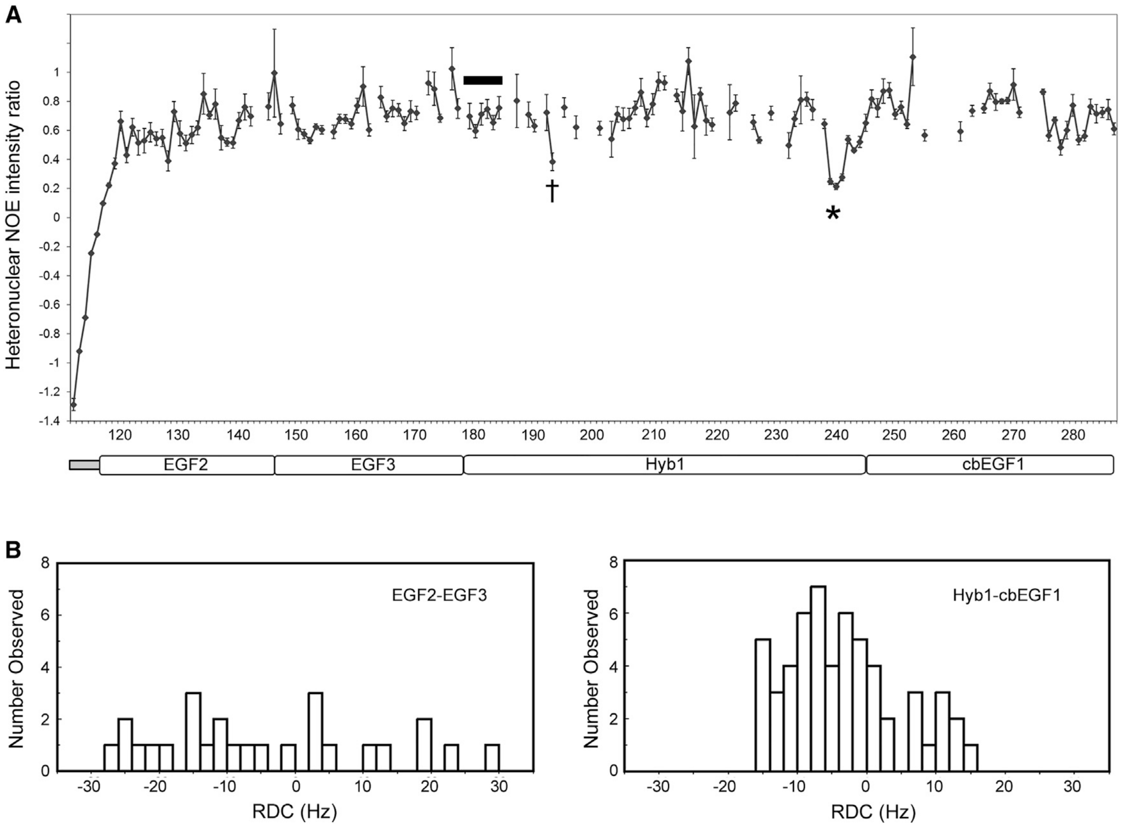

Figure 5. Heteronuclear NOE and RDC Data for FBN1^{E2cbEGF1}

(A) {^{1}H}-^{15}N heteronuclear NOE ratios are plotted as a function of sequence for FBN1^{E2cbEGF1}. Low heteronuclear NOE ratios (<0.5) at the N terminus of EGF2 and for two loops in Hyb1 (†, *) indicate significant flexibility on a picosecond to nanocsecond timescale in these regions. The residues linking EGF3 to Hyb1, indicated by a thick black line, show NOE ratios characteristic of a rigid protein backbone demonstrating that the EGF3-Hyb1 linker is not flexible on a fast timescale. Error bars for the NOE ratio were estimated from 500 Monte Carlo simulations using baseline noise as a measure of the error in the peak heights.

(B) Distribution of RDC values measured in 4% bicelles for the EGF2-EGF3 and Hyb1-cbEGF1 domain pairs of FBN1^{E2cbEGF1}. The different distribution of RDC values for the two pairs suggests that there may be some slower timescale flexibility at the EGF3-Hyb1 interface. Similar differences in RDC distribution are observed for RDCs measured in 2.2% C12E6/n-hexanol (data not shown).

LTBP1 binding site. The similar strength of interaction of LTBP1^{cbEGF14TB3} with FBN1^{NE3} and FBN1^{E2cbEGF1} indicates that the TB3 domain of LTBP1 must interact with the EGF2-EGF3 region of FBN1. The lack of an interaction between LTBP1^{E3cbEGF15} and FBN1^{NE3} suggests that the EGF3 domain of LTBP1 must interact with the Hyb1-cbEGF1 region of FBN1.

Dissociation constants (K_d) of ~100 ± 20 and ~300 ± 100 μM for the interaction of FBN1^{E2cbEGF1} with LTBP1^{E3cbEGF15}, and LTBP1^{cbEGF14TB3}, respectively, can be estimated from the SPR data (Figure S2). In contrast, the binding of LTBP1^{TB3E3} to FBN1^{E2cbEGF1} gives a non-linear Scatchard plot (Figure S2); this is not surprising as the multi-site mode of interaction for this LTBP1 construct may give rise to complicated binding kinetics. Nevertheless, a K_d of ~0.5–1 μM can be estimated from the SPR data at the lowest analyte concentrations (which are similar to concentrations used in previous studies [Massam-Wu et al., 2010; Ono et al., 2009]). Thus, the pair of interaction sites between FBN1 and LTBP1 results in a substantial enhancement in overall binding affinity. A cartoon summarizing our proposed binding model based on our domain dissection data is shown in Figure 2C.

HSQC Titrations of FBN1 and LTBP1 Reveal Distinct Binding Sites

The SPR studies described above indicated that two sites are responsible for the interaction of the C-terminal region of LTBP1 and the N-terminal region of FBN1. To provide residue-specific information about each binding site, NMR titrations were carried out with a number of ^{15}N-labeled protein constructs with assigned heteronuclear single quantum coherence (HSQC) spectra (Robertson et al., 2013b, 2014). Titrations of the four-domain FBN1^{E2cbEGF1} construct with the two-domain LTBP1 construct, LTBP1^{cbEGF14TB3}, exhibit specific chemical shift changes which identify residues that are involved in the interaction (Figures 3A and 3B); this fast exchange behavior on the NMR timescale is consistent with the relatively weak interaction between the two protein constructs seen by SPR. These titrations show that residues throughout the TB3 domain of LTBP1 interact with residues located in EGF3 and the N-terminal region of Hyb1 of FBN1 (Figures 3A and 3B). Titration of the four-domain FBN1^{E2cbEGF1} construct with the two-domain LTBP1 construct, LTBP1^{E3cbEGF15}, shows specific broadening effects rather than chemical shift changes; this indicates intermediate/slow

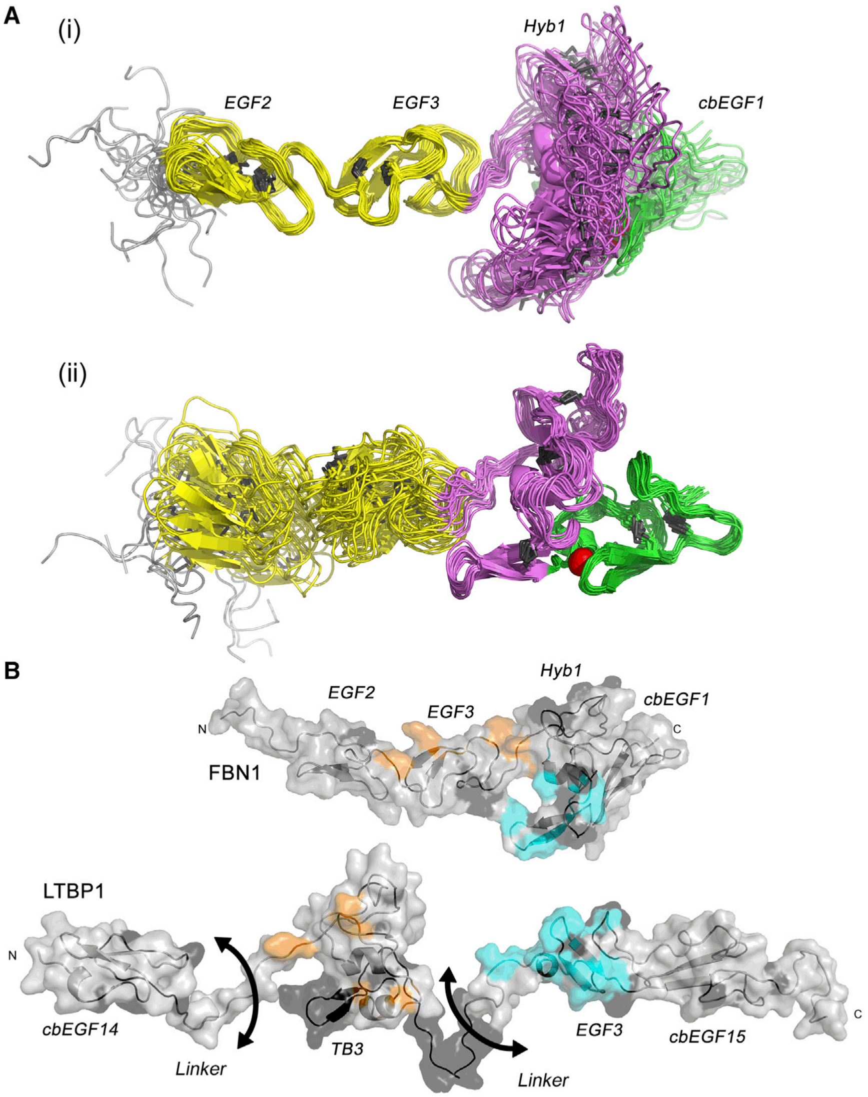

Figure 6. Solution Structural Ensemble of FBN1^{E2cbEGF1} and Interaction Sites Mapped onto the FBN1 and LTBP1 Structures

(A) Cartoon representation of the 20-structure ensemble of FBN1^{E2cbEGF1} with structures aligned to (i) the EGF2-EGF3 domain pair or (ii) the Hyb1-cbEGF1 domain pair. The EGF, Hyb, and cbEGF domains are colored in yellow, purple, and green, respectively, the calcium ion is shown as a red sphere, and disulfide bonds are shown as dark gray lines.

(B) Residues identified by peak shifts or peak broadening in HSQC spectra from FBN1 and LTBP1 titrations are highlighted on the solution structure of the EGF2-EGF3-Hyb1-cbEGF1 region of FBN1 and on a validated homology model of the cbEGF14-TB3-EGF3-cbEGF15 region of LTBP1 (Robertson et al., 2011, 2014). Residues in FBN1 and LTBP1 that experience combined peak shifts of more than 0.06 and 0.05 ppm, respectively, in titrations of FBN1^{E2cbEGF1} with LTBP1^{cb14TB3} are shown in orange. Residues in FBN1 and LTBP1 that experience at least a 7- or 10-fold loss of intensity, respectively, in titrations of FBN1^{E2cbEGF1} with LTBP1^{E3cbEGF15} are shown in cyan. In both proteins, residues that have shifts or intensity changes below the relevant thresholds are shown in light gray, whereas residues that were not assigned are shown in dark gray. See also Figures S3 and S4.

exchange behavior consistent with the stronger interaction between this pair of protein constructs seen by SPR (Figures 3C and 3D). These titrations show that residues located in the EGF3 domain of LTBP1, and some of the unstructured region that immediately precedes EGF3, interact with residues located in the Hyb1 and cbEGF1 domains of FBN1 (Figures 3C and 3D).

SAXS and NMR Support an Extended Near-Linear Conformation for FBN1^{E2cbEGF1} in Solution

To investigate the overall shape of the FBN1^{E2cbEGF1} fragment and to assess its flexibility in solution, SAXS measurements were collected. *Ab initio* structural modeling and analysis of P(r) distributions support the idea of the EGF2-EGF3-Hyb1-cbEGF1 fragment adopting a linear conformation in solution. The Kratky plot is also consistent with a folded protein and does not show signs of significant flexibility (Figures 4A–4C).

In our previous studies of the C-terminal domains of LTBP1, $\{^1H\}$-^{15}N heteronuclear nuclear Overhauser effect (NOE) data identified the presence of flexible linkers between the cbEGF14, TB3, and EGF3 domains (Robertson et al., 2014). Heteronuclear NOE data collected here for FBN1^{E2cbEGF1} show fast timescale flexibility at the N terminus of EGF2 and for loop regions in Hyb1 (Figure 5A). However, no evidence of fast timescale dynamics is observed for the residues linking EGF3 to Hyb1. Furthermore inter-domain ^1H-^1H NOEs are observed between residues at the C terminus of EGF3 and the N terminus of Hyb1, consistent with the presence of a specific interface (data not shown).

Residual dipolar couplings (RDCs) were collected for FBN1^{E2cbEGF1}. RDCs are a useful NMR parameter for assessing the relative orientations of protein domains in solution (Braddock et al., 2001; Weisshuhn et al., 2016) and were used previously to confirm the linear orientation of LTBP1 EGF3-cbEGF15 (Robertson et al., 2014). The distribution of RDCs observed for the EGF2-EGF3 and Hyb1-cbEGF1 pairs are not the same, indicating that some slower timescale movement of the domain pairs relative to each other may exist in solution (Figure 5B). Overall, these NMR data suggest that EGF3 and Hyb1 form an interface, but this may not be quite as rigid in solution as the EGF2-EGF3 and Hyb1-cbEGF1 interfaces.

Solution Structure of FBN1^{E2cbEGF1}

The solution structure of the N-terminal region of FBN1, comprising the unique N-terminal domain (FUN) and EGF1-3 (Figure 1A), has been determined previously (Yadin et al., 2013). This showed a rigid orientation of the FUN-EGF1 pair, a

Table 1. NMR Structure Calculation Statistics

	$FBN1^{E2cbEGF1}$
NOE-derived distance restraints	
Total	3,222
Total unambiguous	3,072
Intra-residue	1,261
Inter-residue	1,811
Sequential ($\|i-j\| = 1$)	726
Short-range ($\|i-j\| < 5$)	351
Long-range ($\|i-j\| \geq 5$)	734
Ambiguous	150
Hydrogen bond restraints	78
Dihedral angle restraints	161
RDCs	169
$^{1}D_{NH}$ 2.2% C12E6/n-hexanol	85
$^{1}D_{NH}$ 4% bicelles	84
Calcium-binding restraints	7
Restraint violations (average of full ensemble)	
Distance restraint violations >0.5 Å	4.35
Dihedral angle violations >5°	2.85
RMSD from experimental restraints	
Distance restraints (Å)	0.057 ± 0.003
Dihedral angle restraints (°)	1.323 ± 0.402
RDC restraints (Hz)	2.155 ± 0.141
RMSD from idealized geometry	
Bonds (Å)	0.007 ± 0.000
Angles (°)	0.822 ± 0.021
Impropers (°)	2.50 ± 0.12
Ramachandran plot (%)	
Residues in most favored regions	80 ± 2
Residues in additional allowed regions	15 ± 2
Outlier residues	5 ± 1
Coordinate precision (RMSD; Å)	
Backbone	
EGF2-EGF3 residues 119–178	0.80
Hyb1-cbEGF1 residues 204–287	0.71
Heavy atom	
EGF2-EGF3 residues 119–178	1.17
Hyb1-cbEGF1 residues 204–287	1.15

NOE, nuclear Overhauser effect; RDCs, Residual dipolar couplings; RMSD, root-mean-square deviation.

flexible linker between EGF1 and EGF2, and a rigid orientation of the EGF2-EGF3 pair. Structural information for the N-terminal region of FBN1 beyond EGF3, which contains the main LTBP1 binding site, has not been available. Attempts to crystallize the four-domain $FBN1^{E2cbEGF1}$ construct were unsuccessful.

Here, the solution structure of $FBN1^{E2cbEGF1}$ was determined by a simulated annealing approach using distance restraints derived from 2D and 3D nuclear Overhauser effect spectroscopy (NOESY) spectra, torsion angle restraints derived from TALOS+ analysis of chemical shifts (Shen et al., 2009), hydrogen bond restraints derived from hydrogen-deuterium exchange experiments, RDC restraints collected in two alignment media, and a linearity restraint consistent with the SAXS data (Figure 6A; Table 1). Each domain within $FBN1^{E2cbEGF1}$ adopts its expected characteristic fold. The EGF2-EGF3 and Hyb1-cbEGF1 pairs each contain a rigid interface defined by extensive NOEs and RDCs, and superposition of these domain pairs results in relatively low root-mean-square deviation values (Table 1). The structure and inter-domain interactions of the EGF2-EGF3 domain pair are consistent with the previously published solution structure of $FBN1^{NE3}$ (Yadin et al., 2013). However, the C-terminal loop of EGF3 is better defined in the $FBN1^{E2cbEGF1}$ structure presented here since residues in this region are involved in specific packing interactions with the Hyb1 domain that are absent in $FBN1^{NE3}$ (Robertson et al., 2013b). The interface formed between Hyb1 and cbEGF1 is characterized by a number of specific side-chain interactions (Figure S3). The side chains of F235 (Hyb1) and V266 (cbEGF1) form a packing interaction that is likely to stabilize the Ca^{2+}-binding site, as similar packing interactions are observed in other high-affinity Hyb-cbEGF and TB-cbEGF pairs (Jensen et al., 2009).

The EGF3-Hyb1 interface is less well defined in the solution structures (Figure S4). NOEs are observed between residues 170–173 in EGF3 and residues 179–183 in Hyb1. The latter residues are restricted to the very N terminus of Hyb1, which is well defined with respect to the EGF2-EGF3 domain pair. The ambiguity in domain orientation hinges around residues 183 and 184, with only limited NOEs between these "linking" Hyb1 residues preceding 184 and residues 203, 204, 205, and 217 in the rest of the Hyb1 domain.

The ensemble of 20 solution structures has been used to back calculate the SAXS data. All structures give good agreement with the data with χ^2 values ranging from 2.7 to 4.6. The overall shape of the solution structure fits well with the envelope determined from the SAXS data, supporting an extended arrangement of the domains (Figures 4D and 4E).

Structural Modeling and Mutagenesis of the LTBP1-FBN1 Complex

The NMR titration data (Figure 3) can be mapped onto the structure of $FBN1^{E2cbEGF1}$ and our previous model of the cbEGF14-TB3-EGF3-cbEGF15 domains of LTBP1 (Robertson et al., 2014). Two distinct binding sites are apparent that suggest two different faces of the FBN1 N terminus are involved in interacting with the two separate FBN1-binding domains in the LTBP1 C terminus (Figure 6B). Since attempts to crystallize the complex were unsuccessful, HADDOCK, a data-driven protein-protein docking approach, was used to generate models of the complex (van Zundert et al., 2016).

Initial calculations, based only on the NMR titration data, identified a number of possible binding orientations. One common feature in the models was the presence of salt bridges that were frequently seen between basic residues in FBN1 and acidic residues in LTBP1 (Figures 7A and 7B); different pairs of these residues were involved in the formation of inter-molecular salt bridges in different models. K138 and R182 of FBN1 and D1521 and D1573 in LTBP1 are involved in salt bridges in HADDOCK models of the $FBN1/LTBP1^{TB3}$ complex, while R232 of FBN1 and E1625, E1642 and D1655 of LTBP1 are involved in salt bridges in models of the $FBN1/LTBP1^{E3cbEGF15}$ complex. To test the importance of these potential salt bridges, a number of

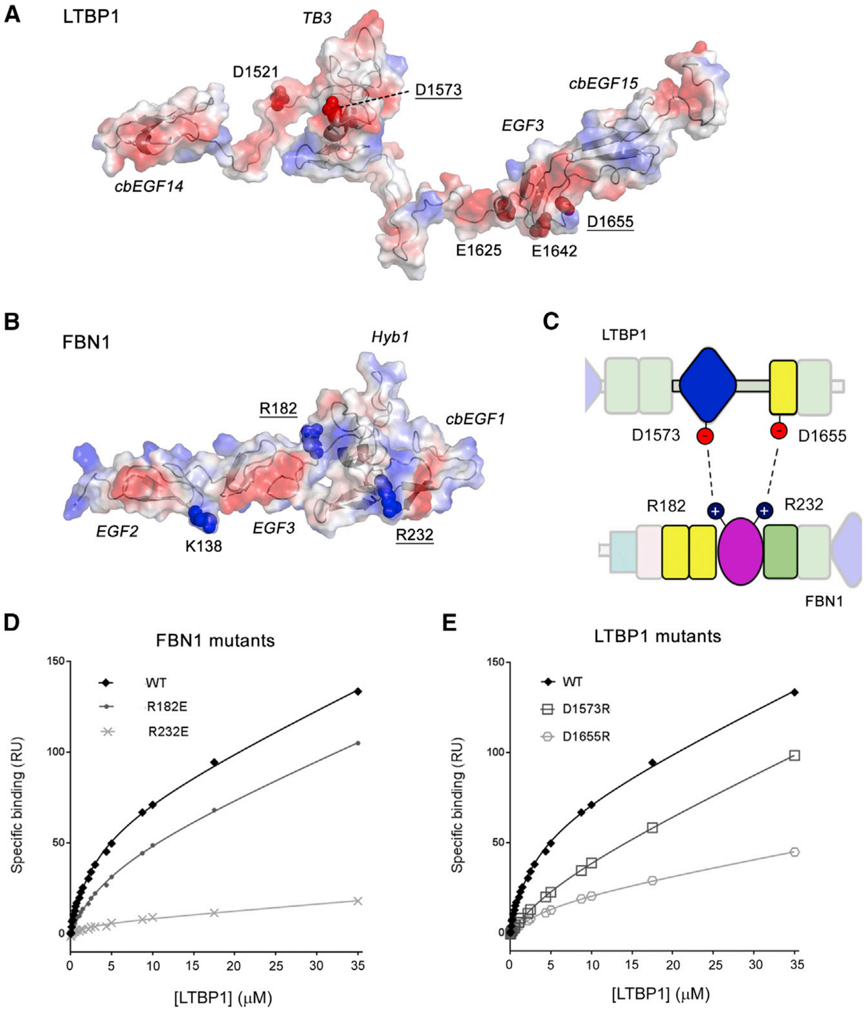

Figure 7. Effects of Salt Bridge Substitutions on FBN1-LTBP1 Interactions

(A) Electrostatic surface representation for LTBP1 (cbEGF14-TB3-EGF3-cbEGF15); negatively charged surface is shown in red and positively charged surface in blue. Acidic residues identified in LTBP1 as potential salt bridge partners by preliminary HADDOCK modeling are shown as dark red spheres. D1521 and D1573 are involved in salt bridges in HADDOCK models of the FBN1-LTBP1^{TB3} complex, while E1625, E1642, and D1655 are involved in salt bridges in models of the FBN1-LTBP1^{E3cbEGF15} complex. Substitution of underlined residues showed reductions in binding.

(B) Electrostatic surface representation for FBN1 (EGF2-EGF3-Hyb1-cbEGF1). Basic residues identified in FBN1 as potential salt bridge partners in initial HADDOCK modeling are shown as dark blue spheres. K138 and R182 are involved in salt bridges in HADDOCK models of the FBN1-LTBP1^{TB3} complex, while R232 is involved in salt bridges in models of the FBN1-LTBP1^{E3cbEGF15} complex.

(C) Simplified model representing charge-charge interactions between the Hyb1 domain of FBN1 and the TB3 and EGF3 domains of LTBP1 with domains colored as in Figure 1.

(D) Plot of SPR responses from binding of WT LTBP1^{TB3E3} analyte to three different FBN1^{E2cbEGF1} constructs immobilized to different flow cells of the same chip.

(E) Plot of SPR responses from three different LTBP1^{TB3E3} constructs binding immobilized WT FBN1^{E2cbEGF1}. See also Figures S1 and S5; Table S2.

charge-reversal substitutions were introduced in FBN1 and LTBP1. Variant proteins were produced (Figure S1) and their interactions assessed using SPR and plate-based assays.

Variants of FBN1 containing the R182E and R232E substitutions, and variants of LTBP1 containing the D1573R and D1655R substitutions, all showed reduced binding to wild-type (WT) LTBP1 and FBN1, respectively (Figures 7D, 7E, and S5), while substitutions K138D, D1521K, E1625R, and E1642R did not (data not shown). D1655R and R232E had the larger effect on binding consistent with a role for these residues in the stronger LTBP1-EGF3/FBN1-Hyb1 interaction (Figure 7C). No further reduction in binding was observed for the interaction of FBN1-R232E and LTBP1-D1655R constructs (Figure S5); if these residues acted independently, as part of two separate salt bridges, then an additive effect and a further reduction in binding would be expected (Hwang and Warshel, 1988; Venkatachalan and Czajkowski, 2008). A similar observation is made for the FBN1-R182E/LTBP1-D1573R interaction. These results are consistent with specific salt bridges forming between R232 and D1655 and between R182 and D1573 in the interaction of FBN1 with LTBP1 (Figure 7C).

The identification of residues in salt bridge interactions stabilizing the complex allowed refinement of the HADDOCK models by including specific distance restraints in each calculation. Figure 8A shows an overall model of the complex created by splicing together the best clusters obtained from docking of LTBP1 TB3 and EGF3-cbEGF15 with FBN1^{E2cbEGF1}. In the HADDOCK model of the FBN1/LTBP1 complex, the distance separating the last residue of LTBP1-TB3 and the first residue of LTBP1-EGF3 is small enough to be easily accommodated by the 36-residue flexible linker joining the two domains (Figure 8A). An LTBP1^{TB3E3} variant in which this linker was deleted showed lower binding to FBN1^{E2cbEGF1} in a plate-based assay (Figures 8B and 8C), consistent with only one of the two LTBP1 domains being able to interact at any given time in this construct. Replacement of the LTBP1 linker with the shorter 22-residue linker from LTBP3, which has a very different amino acid sequence, restored binding to levels comparable with the WT interaction indicating the linker plays a passive role as a connector (Figures 8B and 8C).

Biological Significance of FBN/LTBP Interaction

Our solution structure of a four-domain FBN1 fragment, EGF2-EGF3-Hyb1-cbEGF1, identifies a near-linear domain organization, which, together with the previously determined NE3 structure, reveals the complete structural organization of the N-terminal region of FBN1 (Figure 9A). The extended shape of FBN1, but with a flexible linker between EGF1 and EGF2, may

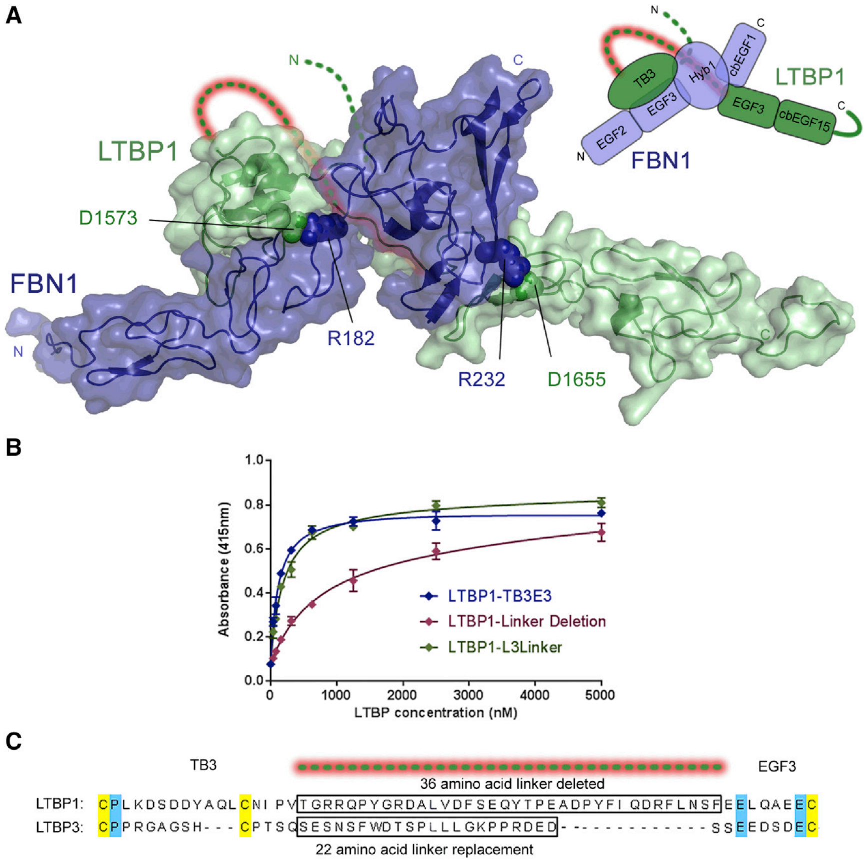

Figure 8. Model of LTBP1-FBN1 Interaction Derived from HADDOCK and Role of LTBP1 TB3-EGF3 Linker in the Interaction

(A) Combined interaction model for FBN1 and LTBP1 created by merging the best models calculated for the two interaction sites. The EGF2-EGF3-Hyb1-cbEGF1 domains of FBN1 are shown in blue and the TB3-EGF3-cbEGF15 domains of LTBP1 are shown in green. The side-chain atoms of residues that form salt bridges are shown as spheres. The dotted lines represent one of many possible positions for the flexible linkers that in LTBP1 join the C terminus of cbEGF14 to the N terminus of TB3 (green dotted line) and the C terminus of TB3 to the N terminus of EGF3 (green dotted line highlighted in red). Inset: cartoon summarizing the layout of protein domains shown.

(B) Plate-binding assay, with the $FBN1^{E2cbEGF1}$ construct immobilized on the plate surface, shows the effect of modifying the TB3-EGF3 linker in LTBP1. Deletion of the linker in $LTBP1^{TB3E3}$ leads to a weakening of the interaction in comparison with WT LTBP1. Replacement of the LTBP1 linker with the LTBP3 linker shows binding similar to the WT construct. The data shown here are representative; they are taken from a single plate with three repeats of each protein concentration carried out to determine experimental error (SD).

(C) Sequence alignment of the TB3-EGF3 region of LTBP1 and LTBP3 shows little or no sequence homology between the two linker regions. Regions of the linker deleted or replaced are highlighted by boxes. See also Figures S1 and S6; Table S1; Data S1.

help expose multiple binding sites that allow FBN1 to act as an interaction hub, with numerous protein-protein interactions reported for this region including LTBP1 and 4 (Ono et al., 2009), fibulin-2, -4, and -5 (Choudhury et al., 2009; El-Hallous et al., 2007; Ono et al., 2009), and ADAMTS10 (Kutz et al., 2011).

Our detailed dissection of LTBP1 binding to FBN1 EGF2-EGF3-Hyb1-cbEGF1 has identified a bipartite mode of interaction, with the C-terminal region of LTBP1 utilizing two discrete sites to anchor itself to FBN1. The two orders of magnitude change in K_d seen when the binding of $LTBP1^{TB3E3}$ (~0.5–1 μM), which contains two binding sites, is compared with the binding of the $LTBP1^{E3cbEGF15}$ and $LTBP1^{cbEGF14TB3}$ fragments (~100 and ~300 μM, respectively), which contain single binding sites, is similar to other bipartite interactions mediated by modular proteins. For example, in a collagen/fibronectin interaction, two individual sites with K_d values of 2 mM and 248 μM, combine to give a K_d of 26 μM (Erat et al., 2013). For fibronectin F1 modules which interact with streptococcal SfbI peptides, two sites with K_d values of 159 and 63 μM combine to give a K_d of 0.45 μM (Schwarz-Linek et al., 2004).

While our measured binding affinities for LTBP fragments, in simple assays, are relatively weak, these affinities are not atypical of other modular protein interactions. Our estimated K_d of ~0.5–1 μM for the $FBN1^{E2cbEGF1}$/$LTBP1^{TB3E3}$ interaction represents a 4- to 25-fold weaker binding than reported in previous SPR studies; the variation in reported K_d values may arise from methodological differences (Massam-Wu et al., 2010; Ono et al., 2009). Since modular ECM proteins often multimerize or have multiple binding sites for proteins/proteoglycans/glycosaminoglycans, additional strategies are likely to further increase binding of LTBP to the matrix. Recently multimerization of LTBP1 has been observed, which is enhanced by heparin (Troilo et al., 2016). Higher-order assembly of LTBP1, together with the bipartite interaction reported here, may allow LTBP1 to achieve a strong interaction with FBN1 in the dynamic environment of connective tissues, while maintaining contacts with other ECM protein networks and/or cell/matrix components (Figure 9B).

It has been reported previously that the interaction between FBN1 and LTBP1 is much stronger than that involving FBN2 and LTBP1 (Isogai et al., 2003). Comparison of the sequences of human FBN1 and FBN2 shows complete conservation of residues involved in the stronger FBN Hyb1-cbEGF1/LTBP1 EGF3 interface, whereas three substitutions are observed in the weaker FBN EGF3-Hyb1/LTBP1 TB3 interface (Figure S7). A reduction of binding affinity in the latter site could explain the observed differences in binding for FBN1/FBN2. It has also been suggested that the mode of interaction of FBN1 with the LTBP1 and LTBP4 isoforms differs (Ono et al., 2009). Comparison of the sequences of human LTBP1 and LTBP4 shows substitutions in both binding sites (Figure S7). Some of these substitutions alter the electrostatic properties of the LTBPs. Interestingly, an N164S substitution in FBN1 EGF3, associated with dominant ectopia lentis (Comeglio et al., 2002), has been reported previously to

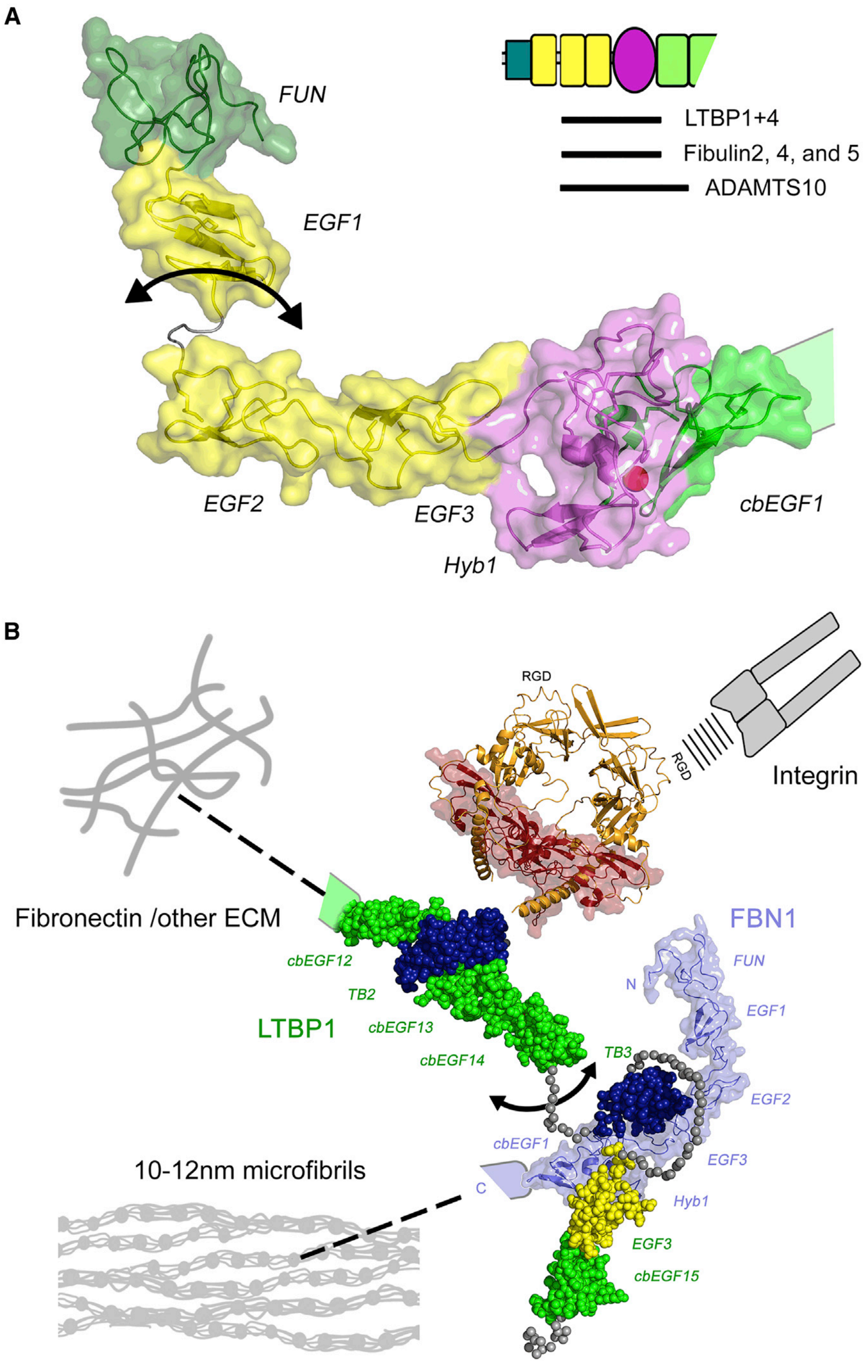

Figure 9. Molecular Model for Presentation of TGF-β by LTBP1 in the ECM

(A) Structure of the FBN1 N terminus constructed from the structure presented here (5MS9) and the previously published structure of NE3 (2M74). The linker between EGF1 and EGF2 is flexible on a fast timescale as shown by an arrow. Domains are colored as in Figure 1 and the top right inset shows the minimal regions required for specific protein interactions.

(B) Model of LAP and fibrillin binding of LTBP1S constructed using the program Modeller and coordinate files PDB: 1UZJ, 1EMN, and 1KSQ. LTBP1 atoms are represented as spheres colored by domain as in Figure 1, while FBN1 is colored slate blue and shown as a ribbon with transparent surface rendering. For the known flexible linkers in LTBP1 only the Cα atoms are shown (gray spheres). For scale, the structure of TGF-β (dark red ribbon and surface) bound to LAP (orange ribbon) from PDB: 3RJR (Shi et al., 2011) is included next to the second TB domain of LTBP1, which binds the small latent complex covalently via a disulfide linkage. LTBP1 can also bind to other ECM fibers via its N terminus (Robertson et al., 2015), and to cell-surface integrins via the RGD motif in LAP. Integrins are thought to bind LAP in order to exert traction and release TGF-β.

decrease the binding affinity of FBN1 to LTBP4, but not to LTBP1 (Ono et al., 2009). N164 is not directly involved in the LTBP1 binding site in our model of the complex; instead its side chain is located on the opposite face of the FBN1 EGF3 domain. The reduction in binding to LTBP4 suggests that the binding interface must be different in the FBN1/LTBP4 complex.

To conceptualize the significance of the LTBP1-FBN1 interaction, a large-scale model of LTBP1 was generated, with the small latent complex of LAP/TGF-β also included for scale (Figure 9B). It can be seen that the short flexible linker identified previously between cbEGF14 and TB3 in LTBP1 (Robertson et al., 2014) may serve as an omnidirectional pivot that helps LTBP to maintain its contacts with other networks and to orient latent TGF-β for activation by integrins or other factors. It is possible that FBN microfibrils (together with other ECM networks) contribute directly to the efficiency of activation of TGF-β since the unusual bipartite nature of the LTBP interaction may allow the LTBP/FBN complex to resist integrin-mediated traction or other dynamic changes in connective tissue, while TGF-β is released from the LAP complex. Integrin-mediated activation of TGF-β has been shown to be heavily reliant on matrix biomechanics (Buscemi et al., 2011; Klingberg et al., 2014), and at present integrins are the only TGF-β activators that have been clearly demonstrated to be crucial *in vivo* (Robertson and Rifkin, 2014; Yang et al., 2007). In Marfan syndrome, loss-of-function *FBN1* mutations result in a deficiency of 10–12 nm microfibrils in the ECM. While LTBP may still be able to bind to other ECM networks such as fibronectin, despite the reduction in FBN, ECM biomechanics may be altered sufficiently in the absence of microfibrils that TGF-β is inappropriately released from the LAP complex when integrins bind. This might explain why *in vivo* deletion of the Hyb1 domain, which does not impair microfibril assembly and contains the major binding site for LTBP1, did not result in any Marfan-like phenotypes (Charbonneau et al., 2010).

Proteases like BMP1 have been proposed to regulate TGF-β signaling during normal development by cleaving the C terminus of LTBP1 and releasing it from the ECM (Ge and Greenspan, 2006). The presence of a flexible linker between the two FBN1-interacting sites within LTBP1 suggests a mechanism by which proteases can regulate the affinity of this interaction. Protease cleavage of the TB3–EGF3 linker in LTBP1 may release the large latent complex from the 10–12 nm microfibril network, as the affinity of the TB3 binding site alone for FBN1 is very much weaker than the bipartite interaction. Cleavage may also significantly reduce the affinity of the remaining EGF3-cbEGF15 LTBP1 fragment for FBN1, facilitating turnover.

In summary, we have completed the structure of the FBN1 N-terminal region, explaining its role as an interaction hub in the ECM. We have further demonstrated an unusual bipartite interaction of LTBP1 with FBN1, adjacent to the LAP/TGF-β binding site, which facilitates complex formation in dynamic connective tissues. We propose that this contributes to integrin-mediated activation of TGF-β in FBN1-rich tissues.

STAR★METHODS

Detailed methods are provided in the online version of this paper and include the following:

- KEY RESOURCES TABLE
- CONTACT FOR REAGENT AND RESOURCE SHARING
- EXPERIMENTAL MODEL AND SUBJECT DETAILS
  - Strains Used in Protein Production
- METHOD DETAILS
  - Protein Production and Purification
  - Protein Interaction Experiments
  - NMR Spectroscopy
  - SAXS
  - Structure Determination
  - Modelling the FBN1/LTBP1 Complex Using HADDOCK
- QUANTIFICATION AND STATISTICAL ANALYSIS
- DATA AND SOFTWARE AVAILABILITY
  - Data Resources

ACCESSION NUMBERS

The coordinates of the family of NMR structures of FBN1^{E2cbEGF1} have been deposited in the PDB under accession number PDB: 5MS9.

SUPPLEMENTAL INFORMATION

Supplemental Information includes seven figures, two tables, and one data file and can be found with this article online at http://dx.doi.org/10.1016/j.str.2017.06.003.

AUTHOR CONTRIBUTIONS

P.A.H., S.A.J., C.R., and I.B.R. designed the study. I.B.R., H.D., and I.H.O. produced recombinant protein samples and performed the plate-binding and SPR experiments. I.B.R. and C.R. performed the NMR experiments and the solution structure calculations. E.D.L. and I.B.R. performed the SAXS experiments. I.B.R. performed the docking calculations. P.A.H., C.R., and I.B.R analyzed the results and wrote the manuscript. All authors discussed the results and implications and commented on the manuscript at all stages.

ACKNOWLEDGMENTS

This work was supported by a Medical Research Council grant (MR/M009831/1) to P.A.H. and C.R. I.B.R. was supported by a Biotechnology and Biological Sciences Research Council studentship. S.A.J. was supported by an Arthritis Research UK grant (20785). SAXS experiments were performed on beamline 29 at the European Synchrotron Radiation Facility (ESRF), Grenoble, France. We are grateful to Petra Pernot at the ESRF for providing assistance. We would also like to thank David Yadin and Karthik Rajasekar for their assistance with NMR structure calculations.

Received: February 3, 2017
Revised: May 5, 2017
Accepted: June 1, 2017
Published: June 29, 2017

REFERENCES

Braddock, D.T., Cai, M., Baber, J.L., Huang, Y., and Clore, G.M. (2001). Rapid identification of medium- to large-scale interdomain motion in modular proteins using dipolar couplings. J. Am. Chem. Soc. *123*, 8634–8635.

Buscemi, L., Ramonet, D., Klingberg, F., Formey, A., Smith-Clerc, J., Meister, J.J., and Hinz, B. (2011). The single-molecule mechanics of the latent TGF-beta1 complex. Curr. Biol. *21*, 2046–2054.

Charbonneau, N.L., Carlson, E.J., Tufa, S., Sengle, G., Manalo, E.C., Carlberg, V.M., Ramirez, F., Keene, D.R., and Sakai, L.Y. (2010). In vivo studies of mutant fibrillin-1 microfibrils. J. Biol. Chem. *285*, 24943–24955.

Chen, V.B., Arendall, W.B., 3rd, Headd, J.J., Keedy, D.A., Immormino, R.M., Kapral, G.J., Murray, L.W., Richardson, J.S., and Richardson, D.C. (2010). MolProbity: all-atom structure validation for macromolecular crystallography. Acta Crystallogr. D Biol. Crystallogr. *66*, 12–21.

Choudhury, R., McGovern, A., Ridley, C., Cain, S.A., Baldwin, A., Wang, M.C., Guo, C., Mironov, A., Jr., Drymoussi, Z., Trump, D., et al. (2009). Differential regulation of elastic fiber formation by fibulin-4 and -5. J. Biol. Chem. *284*, 24553–24567.

Cohn, R.D., van Erp, C., Habashi, J.P., Soleimani, A.A., Klein, E.C., Lisi, M.T., Gamradt, M., ap Rhys, C.M., Holm, T.M., Loeys, B.L., et al. (2007). Angiotensin II type 1 receptor blockade attenuates TGF-beta-induced failure of muscle regeneration in multiple myopathic states. Nat. Med. *13*, 204–210.

Comeglio, P., Evans, A.L., Brice, G., Cooling, R.J., and Child, A.H. (2002). Identification of FBN1 gene mutations in patients with ectopia lentis and marfanoid habitus. Br. J. Ophthalmol. *86*, 1359–1362.

Cook, J.R., Carta, L., Benard, L., Chemaly, E.R., Chiu, E., Rao, S.K., Hampton, T.G., Yurchenco, P., Costa, K.D., Hajjar, R.J., et al. (2014). Abnormal muscle mechanosignaling triggers cardiomyopathy in mice with Marfan syndrome. J. Clin. Invest. *124*, 1329–1339.

Cook, J.R., Clayton, N.P., Carta, L., Galatioto, J., Chiu, E., Smaldone, S., Nelson, C.A., Cheng, S.H., Wentworth, B.M., and Ramirez, F. (2015). Dimorphic effects of transforming growth factor-beta signaling during aortic aneurysm progression in mice suggest a combinatorial therapy for Marfan syndrome. Arterioscler. Thromb. Vasc. Biol. *35*, 911–917.

Dallas, S.L., Miyazono, K., Skerry, T.M., Mundy, G.R., and Bonewald, L.F. (1995). Dual role for the latent transforming growth factor-beta binding protein in storage of latent TGF-beta in the extracellular matrix and as a structural matrix protein. J. Cell Biol. *131*, 539–549.

Dallas, S.L., Keene, D.R., Bruder, S.P., Saharinen, J., Sakai, L.Y., Mundy, G.R., and Bonewald, L.F. (2000). Role of the latent transforming growth factor beta binding protein 1 in fibrillin-containing microfibrils in bone cells in vitro and in vivo. J. Bone Miner. Res. *15*, 68–81.

Delaglio, F., Grzesiek, S., Vuister, G.W., Zhu, G., Pfeifer, J., and Bax, A. (1995). Nmrpipe – a multidimensional spectral processing system based on unix pipes. J. Biomol. NMR *6*, 277–293.

Downing, A.K., Knott, V., Werner, J.M., Cardy, C.M., Campbell, I.D., and Handford, P.A. (1996). Solution structure of a pair of calcium-binding

epidermal growth factor-like domains: implications for the Marfan syndrome and other genetic disorders. Cell *85*, 597–605.

Doyle, J.J., Gerber, E.E., and Dietz, H.C. (2012). Matrix-dependent perturbation of TGFbeta signaling and disease. FEBS Lett. *586*, 2003–2015.

El-Hallous, E., Sasaki, T., Hubmacher, D., Getie, M., Tiedemann, K., Brinckmann, J., Batge, B., Davis, E.C., and Reinhardt, D.P. (2007). Fibrillin-1 interactions with fibulins depend on the first hybrid domain and provide an adaptor function to tropoelastin. J. Biol. Chem. *282*, 8935–8946.

Erat, M.C., Sladek, B., Campbell, I.D., and Vakonakis, I. (2013). Structural analysis of collagen type I interactions with human fibronectin reveals a cooperative binding mode. J. Biol. Chem. *288*, 17441–17450.

Ge, G., and Greenspan, D.S. (2006). BMP1 controls TGFbeta1 activation via cleavage of latent TGFbeta-binding protein. J. Cell Biol. *175*, 111–120.

Habashi, J.P., Doyle, J.J., Holm, T.M., Aziz, H., Schoenhoff, F., Bedja, D., Chen, Y., Modiri, A.N., Judge, D.P., and Dietz, H.C. (2011). Angiotensin II type 2 receptor signaling attenuates aortic aneurysm in mice through ERK antagonism. Science *332*, 361–365.

Hwang, J.K., and Warshel, A. (1988). Why ion pair reversal by protein engineering is unlikely to succeed. Nature *334*, 270–272.

Isogai, Z., Ono, R.N., Ushiro, S., Keene, D.R., Chen, Y., Mazzieri, R., Charbonneau, N.L., Reinhardt, D.P., Rifkin, D.B., and Sakai, L.Y. (2003). Latent transforming growth factor beta-binding protein 1 interacts with fibrillin and is a microfibril-associated protein. J. Biol. Chem. *278*, 2750–2757.

Jensen, S.A., Iqbal, S., Lowe, E.D., Redfield, C., and Handford, P.A. (2009). Structure and interdomain interactions of a hybrid domain: a disulphide-rich module of the fibrillin/LTBP superfamily of matrix proteins. Structure *17*, 759–768.

Jensen, S.A., Robertson, I.B., and Handford, P.A. (2012). Dissecting the fibrillin microfibril: structural insights into organization and function. Structure *20*, 215–225.

Jovanovic, J., Takagi, J., Choulier, L., Abrescia, N.G.A., Stuart, D.I., van der Merwe, P.A., Mardon, H.J., and Handford, P.A. (2007). alpha V beta 6 is a novel receptor for human fibrillin-1. Comparative studies of molecular determinants underlying integrin-RGD affinity and specificity. J. Biol. Chem. *282*, 6743–6751.

Kay, L.E., Torchia, D.A., and Bax, A. (1989). Backbone dynamics of proteins as studied by N-15 inverse detected heteronuclear NMR-spectroscopy – application to staphylococcal nuclease. Biochemistry *28*, 8972–8979.

Keene, D.R., Maddox, B.K., Kuo, H.J., Sakai, L.Y., and Glanville, R.W. (1991). Extraction of extendable beaded structures and their identification as fibrillin-containing extracellular matrix microfibrils. J. Histochem. Cytochem. *39*, 441–449.

Klingberg, F., Chow, M.L., Koehler, A., Boo, S., Buscemi, L., Quinn, T.M., Costell, M., Alman, B.A., Genot, E., and Hinz, B. (2014). Prestress in the extracellular matrix sensitizes latent TGF-beta1 for activation. J. Cell Biol. *207*, 283–297.

Knott, V., Downing, A.K., Cardy, C.M., and Handford, P. (1996). Calcium binding properties of an epidermal growth factor-like domain pair from human fibrillin-1. J. Mol. Biol. *255*, 22–27.

Kuszewski, J., Gronenborn, A.M., and Clore, G.M. (1996). Improving the quality of NMR and crystallographic protein structures by means of a conformational database potential derived from structure databases. Protein Sci. *5*, 1067–1080.

Kutz, W.E., Wang, L.W., Bader, H.L., Majors, A.K., Iwata, K., Traboulsi, E.I., Sakai, L.Y., Keene, D.R., and Apte, S.S. (2011). ADAMTS10 protein interacts with fibrillin-1 and promotes its deposition in extracellular matrix of cultured fibroblasts. J. Biol. Chem. *286*, 17156–17167.

Lima, B.L., Santos, E.J., Fernandes, G.R., Merkel, C., Mello, M.R., Gomes, J.P., Soukoyan, M., Kerkis, A., Massironi, S.M., Visintin, J.A., et al. (2010). A new mouse model for Marfan syndrome presents phenotypic variability associated with the genetic background and overall levels of FBN1 expression. PLoS One *5*, e14136.

Massam-Wu, T., Chiu, M., Choudhury, R., Chaudhry, S.S., Baldwin, A.K., McGovern, A., Baldock, C., Shuttleworth, C.A., and Kielty, C.M. (2010). Assembly of fibrillin microfibrils governs extracellular deposition of latent TGF beta. J. Cell Sci. *123*, 3006–3018.

Neptune, E.R., Frischmeyer, P.A., Arking, D.E., Myers, L., Bunton, T.E., Gayraud, B., Ramirez, F., Sakai, L.Y., and Dietz, H.C. (2003). Dysregulation of TGF-beta activation contributes to pathogenesis in Marfan syndrome. Nat. Genet. *33*, 407–411.

Ono, R.N., Sengle, G., Charbonneau, N.L., Carlberg, V., Bachinger, H.P., Sasaki, T., Lee-Arteaga, S., Zilberberg, L., Rifkin, D.B., Ramirez, F., et al. (2009). Latent transforming growth factor beta-binding proteins and fibulins compete for fibrillin-1 and exhibit exquisite specificities in binding sites. J. Biol. Chem. *284*, 16872–16881.

Ottiger, M., and Bax, A. (1998). Characterization of magnetically oriented phospholipid micelles for measurement of dipolar couplings in macromolecules. J. Biomol. NMR *12*, 361–372.

Ottiger, M., Delaglio, F., and Bax, A. (1998). Measurement of J and dipolar couplings from simplified two-dimensional NMR spectra. J. Magn. Reson. *131*, 373–378.

Petoukhov, M.V., Franke, D., Shkumatov, A.V., Tria, G., Kikhney, A.G., Gajda, M., Gorba, C., Mertens, H.D., Konarev, P.V., and Svergun, D.I. (2012). New developments in the ATSAS program package for small-angle scattering data analysis. J. Appl. Crystallogr. *45*, 342–350.

Pfaff, M., Reinhardt, D.P., Sakai, L.Y., and Timpl, R. (1996). Cell adhesion and integrin binding to recombinant human fibrillin-1. FEBS Lett. *384*, 247–250.

Raghunath, M., Unsold, C., Kubitscheck, U., Bruckner-Tuderman, L., Peters, R., and Meuli, M. (1998). The cutaneous microfibrillar apparatus contains latent transforming growth factor-beta binding protein-1 (LTBP-1) and is a repository for latent TGF-beta1. J. Invest. Dermatol. *111*, 559–564.

Ramirez, B.E., and Bax, A. (1998). Modulation of the alignment tensor of macromolecules dissolved in a dilute liquid crystalline medium. J. Am. Chem. Soc. *120*, 9106–9107.

Rieping, W., Habeck, M., Bardiaux, B., Bernard, A., Malliavin, T.E., and Nilges, M. (2007). ARIA2: automated NOE assignment and data integration in NMR structure calculation. Bioinformatics *23*, 381–382.

Robertson, I.B., and Rifkin, D.B. (2014). Unchaining the beast; insights from structural and evolutionary studies on TGFbeta secretion, sequestration, and activation. Cytokine Growth Factor Rev. *24*, 355–372.

Robertson, I.B., and Rifkin, D.B. (2016). Regulation of the bioavailability of TGF-beta and TGF-beta-related proteins. Cold Spring Harb. Perspect. Biol. *8*, http://dx.doi.org/10.1101/cshperspect.a021907.

Robertson, I., Jensen, S., and Handford, P. (2011). TB domain proteins: evolutionary insights into the multifaceted roles of fibrillins and LTBPs. Biochem. J. *433*, 263–276.

Robertson, I.B., Handford, P.A., and Redfield, C. (2013a). Backbone (1)H, (13)C and (15)N resonance assignment of the C-terminal EGF-cbEGF pair of LTBP1 and flanking residues. Biomol. NMR Assign. *8*, 159–163.

Robertson, I.B., Osuch, I., Yadin, D.A., Handford, P.A., Jensen, S.A., and Redfield, C. (2013b). (1)H, (13)C and (15)N resonance assignments for the fibrillin-1 EGF2-EGF3-hybrid1-cbEGF1 four-domain fragment. Biomol. NMR Assign. *8*, 189–194.

Robertson, I.B., Handford, P.A., and Redfield, C. (2014). NMR spectroscopic and bioinformatic analyses of the LTBP1 C-terminus reveal a highly dynamic domain organisation. PLoS One *9*, e87125.

Robertson, I.B., Horiguchi, M., Zilberberg, L., Dabovic, B., Hadjiolova, K., and Rifkin, D.B. (2015). Latent TGF-beta-binding proteins. Matrix Biol. *47*, 44–53.

Rückert, M., and Otting, G. (2000). Alignment of biological macromolecules in novel nonionic liquid crystalline media for NMR experiments. J. Am. Chem. Soc. *122*, 7793–7797.

Saharinen, J., and Keski-Oja, J. (2000). Specific sequence motif of 8-Cys repeats of TGF-beta binding proteins, LTBPs, creates a hydrophobic interaction surface for binding of small latent TGF-beta. Mol. Biol. Cell *11*, 2691–2704.

Sakamoto, H., Broekelmann, T., Cheresh, D.A., Ramirez, F., Rosenbloom, J., and Mecham, R.P. (1996). Cell-type specific recognition of RGD- and non-RGD-containing cell binding domains in fibrillin-1. J. Biol. Chem. *271*, 4916–4922.

Schwarz-Linek, U., Pilka, E.S., Pickford, A.R., Kim, J.H., Hook, M., Campbell, I.D., and Potts, J.R. (2004). High affinity streptococcal binding to human fibronectin requires specific recognition of sequential F1 modules. J. Biol. Chem. *279*, 39017–39025.

Schwieters, C.D., Kuszewski, J.J., Tjandra, N., and Clore, G.M. (2003). The Xplor-NIH NMR molecular structure determination package. J. Magn. Reson. *160*, 65–73.

Sengle, G., and Sakai, L.Y. (2015). The fibrillin microfibril scaffold: a niche for growth factors and mechanosensation? Matrix Biol. *47*, 3–12.

Shen, Y., Delaglio, F., Cornilescu, G., and Bax, A. (2009). TALOS+: a hybrid method for predicting protein backbone torsion angles from NMR chemical shifts. J. Biomol. NMR *44*, 213–223.

Shi, M., Zhu, J., Wang, R., Chen, X., Mi, L., Walz, T., and Springer, T.A. (2011). Latent TGF-beta structure and activation. Nature *474*, 343–349.

Taipale, J., Saharinen, J., Hedman, K., and Keski-Oja, J. (1996). Latent transforming growth factor-beta 1 and its binding protein are components of extracellular matrix microfibrils. J. Histochem. Cytochem. *44*, 875–889.

Troilo, H., Steer, R., Collins, R.F., Kielty, C.M., and Baldock, C. (2016). Independent multimerization of latent TGFβ binding protein-1 stabilized by cross-linking and enhanced by heparan sulfate. Scientific Rep. *6*, http://dx.doi.org/10.1038/srep34347.

van Zundert, G.C., Rodrigues, J.P., Trellet, M., Schmitz, C., Kastritis, P.L., Karaca, E., Melquiond, A.S., van Dijk, M., de Vries, S.J., and Bonvin, A.M. (2016). The HADDOCK2.2 web server: user-friendly integrative modeling of biomolecular complexes. J. Mol. Biol. *428*, 720–725.

Venkatachalan, S.P., and Czajkowski, C. (2008). A conserved salt bridge critical for GABA(A) receptor function and loop C dynamics. Proc. Natl. Acad. Sci. USA *105*, 13604–13609.

Vranken, W.F., Boucher, W., Stevens, T.J., Fogh, R.H., Pajon, A., Llinas, P., Ulrich, E.L., Markley, J.L., Ionides, J., and Laue, E.D. (2005). The CCPN data model for NMR spectroscopy: development of a software pipeline. Proteins *59*, 687–696.

Weisshuhn, P.C., Sheppard, D., Taylor, P., Whiteman, P., Lea, S.M., Handford, P.A., and Redfield, C. (2016). Non-linear and flexible regions of the human notch1 extracellular domain revealed by high-resolution structural studies. Structure *24*, 555–566.

Yadin, D.A., Robertson, I.B., Jensen, S.A., Handford, P.A., and Redfield, C. (2012). (1)H, (13)C and (15)N assignments of the four N-terminal domains of human fibrillin-1. Biomol. NMR Assign. *8*, 75–80.

Yadin, D.A., Robertson, I.B., McNaught-Davis, J., Evans, P., Stoddart, D., Handford, P.A., Jensen, S.A., and Redfield, C. (2013). Structure of the fibrillin-1 N-terminal domains suggests that heparan sulfate regulates the early stages of microfibril assembly. Structure *21*, 1743–1756.

Yang, Z., Mu, Z., Dabovic, B., Jurukovski, V., Yu, D., Sung, J., Xiong, X., and Munger, J.S. (2007). Absence of integrin-mediated TGFbeta1 activation in vivo recapitulates the phenotype of TGFbeta1-null mice. J. Cell Biol. *176*, 787–793.

Zeyer, K.A., and Reinhardt, D.P. (2015). Fibrillin-containing microfibrils are key signal relay stations for cell function. J. Cell Commun. Signal. *9*, 309–325.

STAR★METHODS

KEY RESOURCES TABLE

REAGENT or RESOURCE	SOURCE	IDENTIFIER
Antibodies		
anti-RGS-His antibody conjugated with HRP	Qiagen	34450
Bacterial and Virus Strains		
BL21 pREP4	Lab strain	N/A
XL10 Gold	Agilent	200315
Chemicals, Peptides, and Recombinant Proteins		
$FBN1^{NE3}$	(Yadin et al., 2013)	N/A
$FBN1^{E2cbEGF1}$ and variants	This work and (Robertson et al., 2013b)	N/A
$FBN1^{cbEGF22TB4cbEGF23}$	(Jovanovic et al., 2007)	N/A
$LTBP1^{TB3cbEGF15}$	This work	N/A
$LTBP1^{TB3E3}$ and variants	This work and (Robertson et al., 2014)	N/A
$LTBP1^{cbEGF14TB3}$	This work and (Robertson et al., 2014)	N/A
$LTBP1^{E3cbEGF15}$	This work and (Robertson et al., 2014)	N/A
^{15}N ammonium chloride	Goss Scientific	NLM-467
^{13}C Glucose	Goss Scientific	CLM-1396-5
D_2O	Sigma Aldrich	151882-125 G
Chelating Sepharose	GE Healthcare	17-0575-01
Deposited Data		
BMRB NMR assignments	(Robertson et al., 2013a, 2014; Robertson et al., 2013b)	19078, 18848, 19322
Fibrillin E2cbEGF1 NMR structure	This paper	5MS9
Oligonucleotides		
18 Site directed mutagenesis primers listed in Table S2	This work	N/A
Recombinant DNA		
$FBN1^{NE3}$ – in pQE30 expression vector	(Yadin et al., 2013)	N/A
$FBN1^{E2cbEGF1}$ and variants - in pQE30 expression vector	This work and (Robertson et al., 2013b)	N/A
$FBN1^{cbEGF22TB4cbEGF23}$ – in pQE30 expression vector	(Jovanovic et al., 2007)	N/A
$LTBP1^{TB3cbEGF15}$ – in pQE30 expression vector	This work	N/A
$LTBP1^{TB3E3}$ and variants – in pQE30 expression vector	This work and (Robertson et al., 2014)	N/A
$LTBP1^{cbEGF14TB3}$ – in pQE30 expression vector	This work and (Robertson et al., 2014)	N/A
$LTBP1^{E3cbEGF15}$ – in pQE30 expression vector	This work and (Robertson et al., 2014)	N/A
Software and Algorithms		
ARIA 2.3	(Rieping et al., 2007)	N/A
Xplor-NIH 2.29	(Schwieters et al., 2003)	N/A
MolProbity	(Chen et al., 2010)	N/A
HADDOCK server	(van Zundert et al., 2016)	N/A
TALOS+	(Shen et al., 2009).	N/A
ATSAS software package	(Petoukhov et al., 2012)	N/A
CCPN software	(Vranken et al., 2005)	N/A
NMRPipe	(Delaglio et al., 1995)	N/A
Other		
ELISA plates	R&D systems	DY990
Biacore SPR CM5 sensor chips	GE healthcare	BR100530

CONTACT FOR REAGENT AND RESOURCE SHARING

Further information and requests for resources and reagents should be directed to and will be fulfilled by the Lead Contacts, Penny Handford and Christina Redfield (penny.handford@bioch.ox.ac.uk and christina.redfield@bioch.ox.ac.uk).

EXPERIMENTAL MODEL AND SUBJECT DETAILS

Strains Used in Protein Production

The FBN1 and LTBP1 protein fragments used in this study were expressed in *Escherichia coli* BL21 cells transformed with a pQE-30 (Qiagen) expression vector and pREP4 plasmid for control of expression via the lac repressor. When cloned into the expression vector, an N-terminal His_6 tag was included for purification, followed by an Ser-Ala spacer and a factor Xa protease recognition site (Ile-Glu-Gly-Arg) for later removal of the His_6 tag.

METHOD DETAILS

Protein Production and Purification

Sequences encoding the EGF2-EGF3-Hyb1-cbEGF1 region of human *FBN1* and the cbEGF14-TB3-EGF3-cbEGF15 region of human *LTBP1* were cloned into the pQE30 vector (Qiagen). The additional cysteine in the FBN1 Hyb1 domain (C204) was replaced with a serine as described previously (Jensen et al., 2009). This change was necessary to allow effective in vitro refolding of the FBN1 protein fragment. Site-directed mutagenesis was carried out using the QuikChange protocol (Agilent) (for details of primers used see Table S2).

Protein expression and purification was carried out in a similar fashion to that described previously (Knott et al., 1996) but with modifications for each construct (Robertson et al., 2013a, 2013b, 2014; Yadin et al., 2012). Unlabelled proteins were expressed in *E. coli* grown on unlabelled rich medium. Proteins were single or double labelled with ^{15}N or $^{15}N/^{13}C$ by growing cells in M9 medium containing 0.1% (w/v) $^{15}NH_4Cl$ and 0.5% (w/v) ^{13}C-glucose (Goss Scientific), in the presence of 100 μg/ml ampicillin and 25 μg/ml kanamycin. 50 ml of starter culture, grown in unlabelled M9 medium at 37 °C for ~18 hours, was used to inoculate 600 ml of labelled M9 medium. Bacteria were grown until OD_{600} reached ~0.8, at which point expression was induced with isopropyl-β-D-thiogalactopyranoside (IPTG) at a final concentration of 1 mM. Cells were then incubated at 28 °C for ~20 hours, harvested by centrifugation and frozen at −80 °C prior to protein purification.

Proteins were purified from inclusion bodies (Robertson et al., 2013a, 2013b, 2014; Yadin et al., 2012). The solubilised His-tagged proteins were purified using Ni^{2+} affinity chromatography using fast-flow chelating Sepharose (Amersham Pharmacia or GE Healthcare). His-tagged proteins were eluted with buffer containing 50 mM EDTA. The eluted proteins were then reduced with ~200 mM DTT, buffered with 0.1 M Tris-HCl pH 8.3 and left for at least 1 hr at room temperature to allow for full reduction of the protein. The reduced protein solutions were acidified to pH 2-3 with HCl and dialysed overnight at room temperature against 2 L of 0.1% trifluoroacetic acid (TFA). Protein was then desalted by high performance liquid chromatography (HPLC) using a C8 reverse phase column (Rainin).

Purified, reduced proteins were refolded in an aqueous solution of ~0.2 mg/ml reduced protein, 100 mM Tris-HCl pH 8.3, 3 mM cysteine and 0.3 mM cystine, and up to 50 mM $CaCl_2$; 50% (v/v) glycerol was included for refolding of $FBN1^{E2cbEGF1}$ and $FBN1^{NE3}$ (to enhance protein solubility) but was not necessary for refolding of LTBP1 constructs. The solution was then left for 48-72 hrs at 4°C. After this period the refold mixture was acidified to pH 2-3 with HCl, and dialysed against 0.1% (v/v) TFA overnight. Dialysate was centrifuged and filtered to remove any precipitate, concentrated by ultrafiltration, filtered again, and then purified by HPLC.

The His_6 tag was cleaved off for all constructs, except $LTBP1^{TB3E3}$ and $LTBP1^{TB3cbEGF15}$, by incubation with factor Xa (Novagen), carried out with a protein concentration of 1.5-5 mg/ml and 1 unit factor Xa per mg protein, and incubated at 37 °C overnight. Proteins were further purified by fast protein liquid chromatography (FPLC) using a MonoS 5/50 GL or a MonoQ 5/50 column (GE Healthcare) depending on protein solubility. After FPLC all proteins were acidified to pH ~2, filtered to remove any precipitate, and desalted by further HPLC purification, before final lyophilisation. The final products were analysed by SDS-PAGE (Figure S1), electrospray ionisation mass spectrometry, and 2D ^1H-^1H NOESY spectra, and were shown to be monomeric and correctly folded.

Protein Interaction Experiments

Plate-based interaction experiments were carried out by incubating DY990 plates (R&D systems) with 50 μl $FBN1^{E2cbEGF1}$ in 50 mM carbonate buffer at pH 9.6 for a minimum of 48 hours. Plates were then blocked with 5% BSA carbonate buffer for one hour. Wells were washed with 100 μl interaction buffer (50 mM Tris pH 7.5, 150 mM NaCl, 0.05% (v/v) Tween-20, 2 mM $CaCl_2$) and 50 μl LTBP1 protein samples were then added. At this stage wells were aspirated and aliquoted individually to avoid plate drying. Plates were incubated with LTBP1 samples for 12 hours and then washed. A 1:5000 dilution of an anti-RGS-His antibody conjugated with HRP (Qiagen 34450) was then added to each well and incubated for 1 hour to detect LTBP1 binding. Data presented in figures are representative examples of several experiments carried out at different times (for additional information see QUANTIFICATION AND STATISTICAL ANALYSIS section below).

SPR studies were carried out using a Biacore T100 instrument (GE Healthcare) with FBN1 fragments coupled to the surface of a CM5 sensor chip by amine coupling and then washed with 50 mM HCl. The Biacore coupling wizard was used to ensure that an

equivalent 1000 RU of protein was coupled to each flow cell. The sensor chip was equilibrated with SPR running buffer consisting of 50mM Tris pH 7.5, 150mM NaCl, 0.05% (v/v) Tween 20 and 2mM $CaCl_2$. Freeze-dried LTBP1 proteins were dissolved directly into SPR buffer to generate analyte stock solutions. The protein concentrations in these stock solutions were measured by UV-visible spectroscopy. All SPR experiments were performed at 25°C, with a flow rate of 20 µl per minute, and a 30 second 50mM HCl injection was used for regeneration after each run. Both Multi-cycle and single-cycle programs were used depending on program availability and the volume of data needed. All four flow cells of the chip were used, one as a blank for baseline subtraction, one with the $FBN1^{E2cbEGF1}$ fragment bound, one with $FBN1^{NE3}$ bound, and finally one with a negative control FBN1 fragment spanning the cbEGF22-TB4-cbEGF23 domains bound. In this way all flow cells were simultaneously exposed to the same LTBP1 protein analyte solutions. K_d values were estimated, where possible for lower affinity interactions, from linear Scatchard plots. In some cases, despite the clear specificity of binding (shown by lack of binding to a control FBN1 fragment or the blank and control treated flow cells), the Scatchard plots were non-linear; this is not surprising because the multi-site mode of interaction for the higher affinity LTBP1 constructs may give rise to complicated binding kinetics.

NMR Spectroscopy

NMR experiments were carried out using spectrometers operating at ^1H frequencies ranging from 500 to 950 MHz. The spectrometers were equipped with Oxford Instruments magnets and home-built triple-resonance pulsed-field gradient probes (500, 600, 750 and 950 MHz) or with Bruker Avance consoles and TCI CryoProbes (500 and 750 MHz). NMR data were acquired using either GE/Omega software using pulse sequences written in-house, or Topspin software and pulse sequences in the Topspin libraries from Bruker Biospin. NMR data were processed using NMRPipe (Delaglio et al., 1995) and spectra were analysed using the CCPN software (Vranken et al., 2005).

Resonance Assignments

Resonance assignments for $FBN1^{E2cbEGF1}$, $LTBP1^{E3cbEGF15}$ and $LTBP1^{cbEGF14TB3}$ have been described previously (Robertson et al., 2013a, 2013b, 2014) (BMRB accession numbers 19078, 18848, 19322, respectively). Unless otherwise stated, all NMR experiments were carried out at 25°C at pH 5.4 in 95% H_2O/5% D_2O with 5mM $CaCl_2$.

Protein Interactions Monitored by NMR

Interactions between LTBP1 and FBN1 fragments were monitored using 2D ^1H-^{15}H HSQC spectra collected at 500 MHz. Initially an HSQC spectrum was collected for the ^{15}N-labelled protein sample alone, and then successive freeze dried aliquots of the unlabelled interaction partner were added, with the pH of the NMR sample measured before and after each addition, and adjusted prior to running each HSQC experiment. Initial experiments utilising the $LTBP1^{TB3E3}$ and $FBN1^{E2cbEGF1}$ fragments were not informative because uniform broadening of all signal in the HSQC was observed, most likely due to slow exchange effects caused by strong binding of the large LTBP1 fragment.

The interaction of $LTBP1^{E3cbEGF15}$ with $FBN1^{E2cbEGF1}$ was monitored in two titrations using either ^{15}N-labelled $LTBP1^{E3cbEGF15}$ or ^{15}N-labelled $FBN1^{E2cbEGF1}$. In both titrations 300 µl of a 300 µM ^{15}N sample was used at pH 5.5, with 100 mM NaCl and 10 mM $CaCl_2$. The concentration of the unlabelled $LTBP1^{E3cbEGF15}$ or $FBN1^{E2cbEGF1}$ ligand ranged from 0 to 250 µM. In these titrations, peaks were observed to shift; the combined chemical shift change, reported in Figure 3, was determined as $\Delta_{COMB}=((\Delta_{1HN})^2+(\Delta_{15N}/6)^2)^{1/2}$ where Δ_{1HN} and Δ_{15N} are the observed chemical shift differences for $^1H^N$ and ^{15}N in HSQC spectra collected with 0 and 250 µM ligand protein.

The interaction of $LTBP1^{cbEGF14TB3}$ with $FBN1^{E2cbEGF1}$ was monitored in two titrations using either ^{15}N-labelled $LTBP1^{cbEGF14TB3}$ or ^{15}N-labelled $FBN1^{E2cbEGF1}$. In the first titration 300 µl of a 300 µM ^{15}N-$LTBP1^{cbEGF14TB3}$ sample was used at pH 5.9 with 20 mM $CaCl_2$. In the second titration 290 µl of a 300 µM ^{15}N $FBN1^{E2cbEGF1}$ sample was used at pH 5.3 with 15 mM $CaCl_2$ (slight differences in conditions were necessary to optimise protein solubility). The concentration of the unlabelled $LTBP1^{cbEGF14TB3}$ or $FBN1^{E2cbEGF1}$ ligand ranged from 0 to 250 µM. In these titrations specific losses in peak intensity were seen with successive protein additions. To quantify peak intensity changes during the titration, peak intensities in the first HSQC experiment without added ligand protein were divided by the peak intensities in the final titration point. Errors bars represent the error introduced by background noise in the spectrum.

Heteronuclear NOE

$\{^1H\}$-^{15}N heteronuclear NOE experiment was carried out using ^{15}N-labelled $FBN1^{E2cbEGF1}$ in order to examine the sub-nanosecond dynamics of specific amides (Kay et al., 1989). Spectra with and without ^1H saturation were collected as interleaved experiments collected at 750 MHz. ^1H saturation was applied for 4 s. The data set was acquired with 1K complex points in F_2 and 128 complex t_1 increments; 96 scans were collected per increment. The $\{^1H\}$-^{15}N NOE was calculated as the ratio of the peak intensities in the spectra recorded with and without ^1H saturation. Peak heights were determined using in-house peak-picking software. Uncertainties in the NOE ratios were estimated from 500 Monte Carlo simulations using baseline noise as a measure of the error in the peak heights.

Residual Dipolar Couplings

Residual dipolar couplings (RDCs) were collected for the $FBN1^{E2cbEGF1}$ construct in two liquid crystalline media. One set of RDCs was collected with a 2.2% C12E6/*n*-hexanol (Sigma-Aldrich) solution in 90% H_2O/10% D_2O at pH 5.3 with 5mM calcium chloride (Rückert and Otting, 2000). The second set of RDCs was collected with 4% (w/v) bicelles comprising the ether linked lipids 1,2-O-ditridecyl-*sn*-glycero-3-phosphocholine and 1,2-dihexyl-*sn*-glycero-3-phospho-choline (Avanti Polar Lipids), as well as cetyl trimethyl ammonium bromide (Sigma-Aldrich) in a molar ratio of 35:10:1 in 90% H_2O/10% D_2O at pH 5.5 with 5mM calcium chloride (Ottiger and Bax, 1998; Ramirez and Bax, 1998). Interleaved IPAP experiments (Ottiger et al., 1998) were performed at a ^1H frequency of 600 MHz at 25°C (or 35°C for the bicelles) using 128 and 1024 complex points in F_1 (^{15}N) and F_2 (^1H), respectively. Isotropic spectra were also collected

under comparable conditions and residual dipolar couplings were measured as the difference between the splitting observed in the isotropic and aligned data sets.

SAXS

Small Angle X-ray Scattering data were collected at beamline BM29 at the ESRF in Grenoble, France. Lyophilised protein was dissolved in buffer containing 20 mM MES at pH 5.4, 5 mM $CaCl_2$, and 5% glycerol. This solution was then further dialysed against a large volume of the same buffer and a sample of this buffer used as a blank in the beam line to ensure correct matching. Scattering data were collected at four different protein concentrations and the data from these samples were scaled and averaged for further analysis. Guinier analysis was performed using the SCATTER software package and *ab initio* modelling and structure fitting was performed using the DAMMIF and CRYSOL programs in the ATSAS software package (Petoukhov et al., 2012) *Ab initio* modelling was performed using 'slow' mode with 20 repetitions and default settings for modelling globular proteins. When NMR structures were fitted to the SAXS data using CRYSOL the 7 flexible N-terminal residues of $FBN^{E2cbEGF1}$ were removed as this random unstructured element could significantly affect the fitting.

Structure Determination

NMR experiments for structure determination were carried out using ^{15}N or $^{13}C/^{15}N$ labelled $FBN1^{E2cbEGF1}$ at a concentration of 1 mM at pH 5.4 and 25°C. Distance restraints for structure calculation were derived from several 2D and 3D NOESY spectra. The 3D ^{15}N-edited NOESY-HSQC spectrum (mixing time 150 ms) was acquired at 950 MHz in 95% H_2O/5% D_2O. 3D ^{13}C-edited NOESY-HSQC spectra (mixing time 75 ms) were acquired at 500 and 950 MHz in 95% H_2O/5% D_2O (v/v) and 100% D_2O, respectively. An aromatic 3D ^{13}C-edited NOESY-HSQC spectrum (mixing time 150 ms) was acquired at 750 MHz in 95% H_2O/5% D_2O (v/v). A 2D ^1H-^1H NOESY spectrum (mixing time 150 ms) was acquired at 950 MHz in 100% D_2O. NOESY cross peaks were assigned manually in CCPN Analysis using published resonance assignments (Robertson et al., 2013b). In some cases only ambiguous peak assignments were possible initially.

ϕ and ψ torsion angle restraints were obtained using TALOS+ predictions on the basis of assigned chemical shifts (Shen et al., 2009). Hydrogen bond restraints were based on slowly exchanging amides identified in HSQC spectra collected in D_2O and observed NOEs characteristic of regular secondary structure.

Comparison of the distribution of RDC values for the EGF2-EGF3 pair and the Hyb1-cbEGF1 pair showed that they are not the same indicating that some slower timescale movement of the domain pairs relative to each other may exist in solution (Figure 5B); this was the case for both alignment media used. In order not to bias the definition of the EGF3-Hyb1 interface in structure calculations, the RDC data for the EGF2-EGF3 and Hyb1-cbEGF1 pairs were treated separately in the structure calculations (i.e. different reference alignment tensors used). Values for the axial and rhombic components for the alignment tensors were calculated from the previously determined structure of NE3 and from a homology model for Hyb1-cbEGF1 using in-house software. For the EGF2-EGF3 pair D_a/R values of -13.9/0.56 were used for the bicelle data and values of -19.0/0.54 were used for the C12E6/n-hexanol data. For the Hyb1-cbEGF1 pair D_a/R values of 11.2/0.35 were used for the bicelle data and values of 15.7/0.50 were used for the C12E6/n-hexanol data.

Structures were calculated initially using ARIA 2.3 (Rieping et al., 2007); this was useful for obtaining assignments for ambiguous NOEs and for validating the allocation of NOE restraints into distance bins. However, the four-domain topology and the 13 disulphide bonds in $FBN1^{E2cbEGF1}$ resulted in a limited number of converged structures using this protocol. Subsequent structure calculations were performed using Xplor-NIH 2.29 (Schwieters et al., 2003). Initially, ~800 structures were calculated from an extended starting structure using a simulated annealing protocol (initial T=2000K, 30000 high temperature steps, 40000 and 20000 steps in cooling to 1000K and 100K, respectively) using NOE, hydrogen bond, disulphide bond and torsion angle restraints. The Ca^{2+}-binding site was defined using distance restraints between the Ca^{2+} ion and the cbEGF consensus ligands (Downing et al., 1996). A 'Rama' torsion angle database potential was used (Kuszewski et al., 1996). The 20 lowest energy simulated annealing structures were used as the starting point for refinement (initial T=1500K, 20000 cooling steps) generating a total of 400 structures; the 5 lowest energy structures from each of the 20 starting structures were selected for further refinement using the two sets of RDC restraints. Refinement was carried out using 100 starting structures (initial T=1500K, 20000 cooling steps). A square well potential and a force constant of 0.5 were used for the SANI terms with experimental error for the RDCs in the range of 2-4 Hz. 40 structures were selected from the family of 2000 RDC-refined structures for a final round of water refinement. The water-refined structures were used to predict the SAXS data and a final family of 20 structures was chosen on the basis of low restraint and overall energies and a good fit to the SAXS data. Ramachandran validation statistics were calculated using MolProbity (Chen et al., 2010).

Modelling the FBN1/LTBP1 Complex Using HADDOCK

Docking was carried out using the 'guru' interface of the HADDOCK server (van Zundert et al., 2016). Since the TB3 and EGF3 domains of LTBP1 are connected by a flexible linker and their binding sites were mapped separately using the $LTBP1^{cbEGF14TB3}$ and $LTBP1^{E3cbEGF15}$ constructs, HADDOCK modelling of these two interactions was performed separately. Flexibility was introduced into all protein models, both to account for observed flexible loops and also structural ambiguity. A large number of preliminary calculations was performed using different sets of ambiguous interaction restraints defined using different cut offs applied to the NMR titration data. From these calculations numerous models were generated that were clustered and sorted on the basis of various shared features. The results suggested several possible binding orientations, but one common feature in the models was the

presence of salt bridges that were frequently seen between basic residues in FBN1 and acidic residues in LTBP1. In the different binding orientations identified, each employed different charged residues in the formation of inter-molecular salt bridges, allowing the design of targeted substitutions to test these HADDOCK models. Substitutions introduced were K138D, R182E, and R232E in $FBN1^{E2cbEGF1}$, and D1521K, D1573R, E1625R, E1642R, and D1655R in $LTBP1^{TB3E3}$. Binding of the substituted FBN1 and LTBP1 constructs was assessed using the SPR and plate-based assay as described above for the wild-type constructs.

A final round of HADDOCK calculations was carried out using the restraints derived from the NMR data and the salt bridges identified by the mutagenesis experiments. The final docking clusters shown in Figure 8, and included as a supplemental pdb file (Data S1), were based on the docking parameters shown in Table S1. Active and passive residues used to define the ambiguous interaction restraints were derived from the peak shift and intensity change data shown in Figure 3. Random exclusion of AIR restraints was allowed with 10 partitions (10% of restraints). All molecules were modelled as 'semi-flexible' and specific segments listed in the table were modelled as fully flexible to reflect heteronuclear NOE data and structural ambiguity. Histidine protonation was determined automatically using MolProbity (Chen et al., 2010).

QUANTIFICATION AND STATISTICAL ANALYSIS

Plate-based assay data to probe protein-protein interactions presented in figures are from a single plate; three repeats of each protein concentration were carried out to determine experimental error. The data presented are representative examples of several experiments carried out at different times. The curves fitted to the data were generated using GraphPad with the 'One site – Total binding' option. This takes into account specific binding for which a K_d is fitted, nonspecific binding which is assumed to have a linear dependence on ligand concentration, and background signal. The K_d values extracted are apparent K_ds because the fitting procedure uses total ligand concentration rather than free ligand concentration.

DATA AND SOFTWARE AVAILABILITY

Data Resources

The coordinates of the family of NMR structures of $FBN1^{E2cbEGF1}$ have been deposited in the Protein Data Bank under accession number 5MS9 (see Table 1). Resonance assignments for $FBN1^{E2cbEGF1}$, $LTBP1^{E3cbEGF15}$ and $LTBP1^{cbEGF14TB3}$ have deposited in the BioMagResBank (BMRB) under accession numbers 19078, 18848 and 19322, respectively. A PDB model of the LTBP1-FBN1 interaction, produced by splicing together the two highest scoring HADDOCK result files, is included in the Supplemental Information.

Structure, Volume *25*

Supplemental Information

The N-Terminal Region of Fibrillin-1 Mediates a Bipartite Interaction with LTBP1

Ian B. Robertson, Hans F. Dias, Isabelle H. Osuch, Edward D. Lowe, Sacha A. Jensen, Christina Redfield, and Penny A. Handford

Supplemental Figures

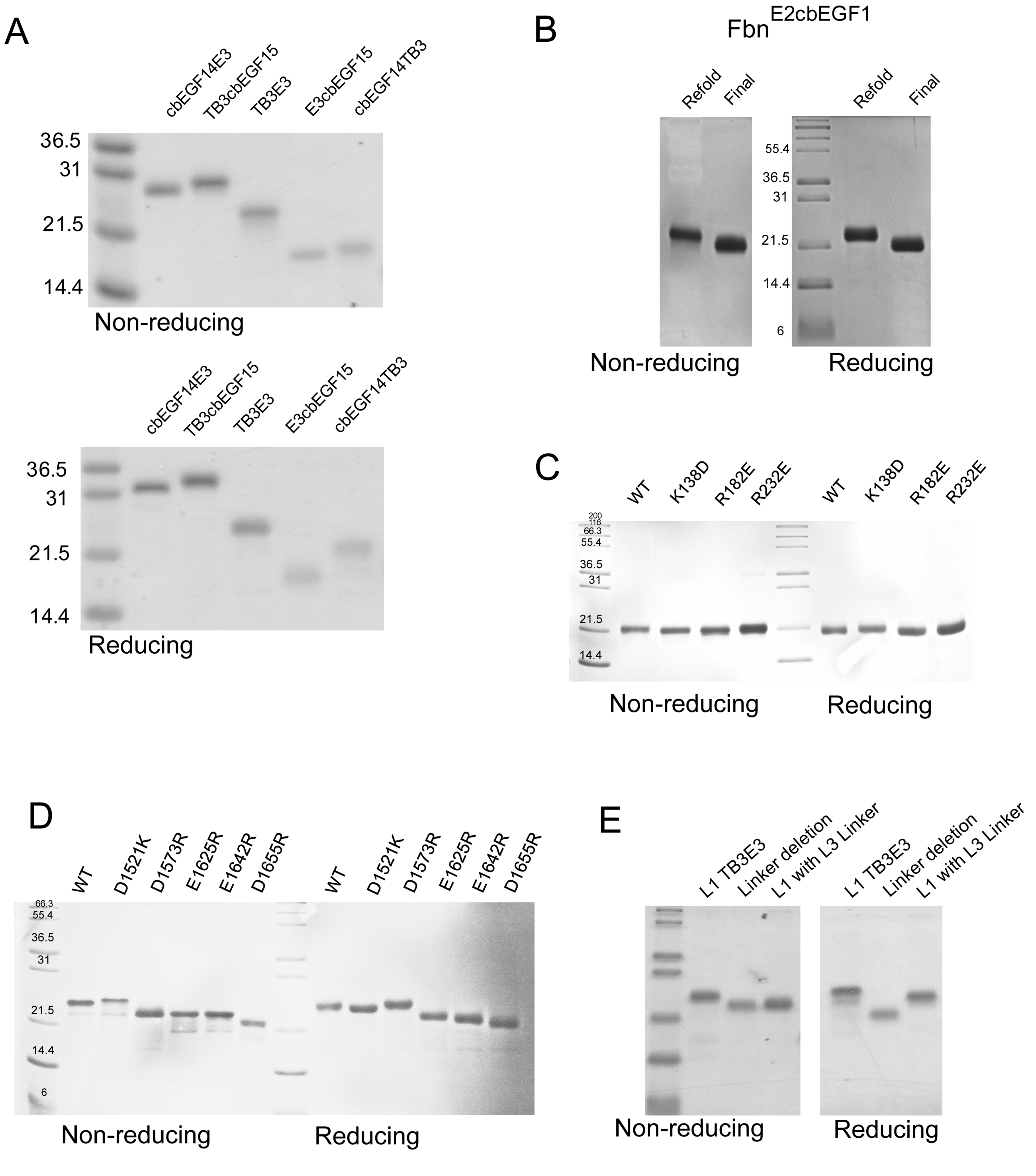

Figure S1. Related to Figure 1, 2, 7 and 8. SDS-PAGE of constructs used in this study.
Coomassie stained SDS-PAGE of protein fragments used in this study. Both non-reducing and reducing gels are shown. (A) Wild-type LTBP1 constructs. (B) FBN1 EGF2-EGF3-Hyb1-cbEGF1 construct. (C) FBN1 variants used to test salt bridges. (D) LTBP1 variants used to test salt bridges. (E) LTBP1 linker variants. All constructs show a good degree of purity under both reducing and non-reducing conditions. Mark 12 (Invitrogen) was used as the protein standard.

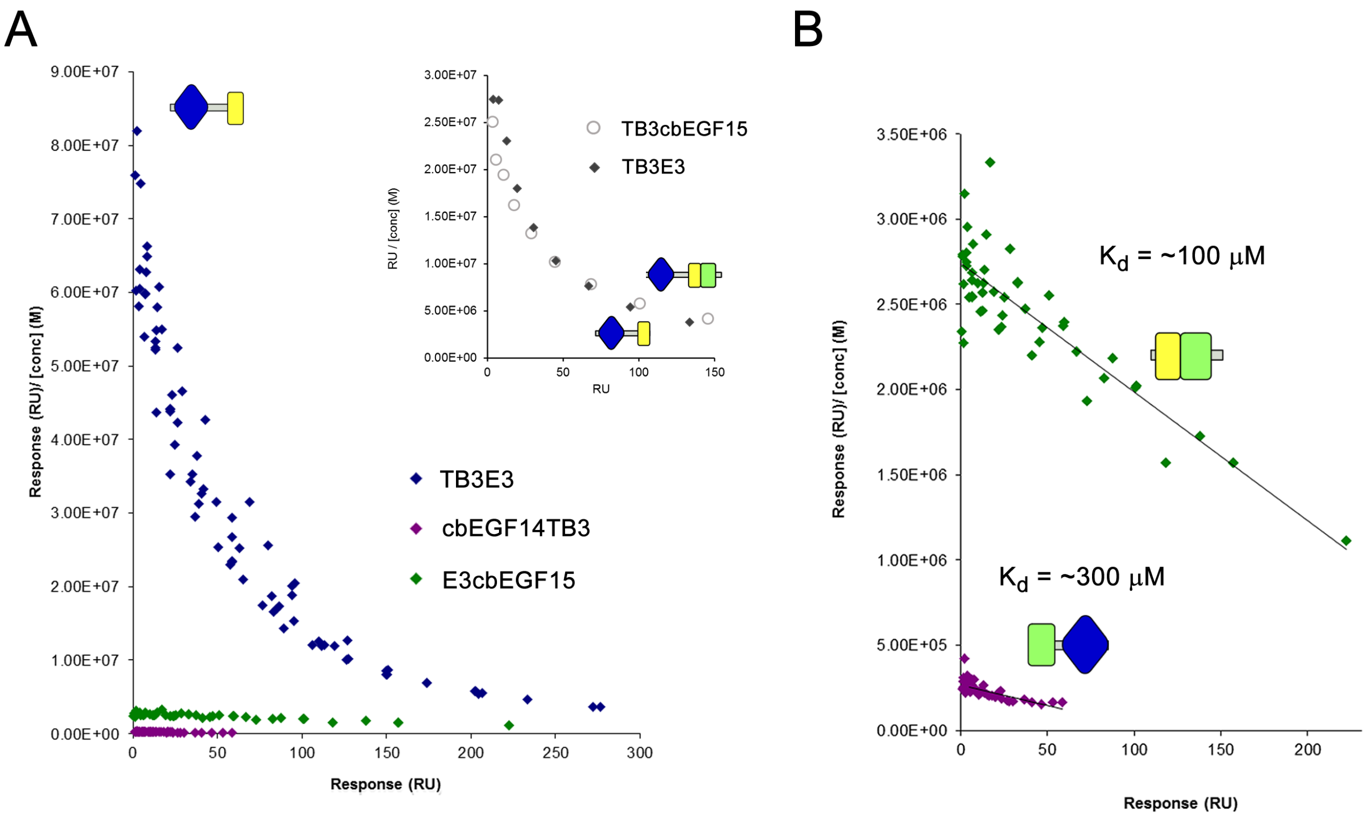

Figure S2. Related to Figure 1 and 2. Scatchard plots

Scatchard plots of single-cycle SPR data. In A) it can be seen that the interaction of LTBP1^{TB3E3} with FBN1^{E2cbEGF1} gives a non-linear Scatchard plot both with the single-cycle SPR method (main) and multi-cycle method (inset). This is consistent with complex kinetics of this interaction and makes determination of a single K_d impossible. Using the lowest concentration data from the single-cycle SPR, a K_d value in the range of 0.5-1μM can be estimated. In the multi-cycle data (shown in the inset) it is clear that LTBP1^{TB3cbEGF15} and LTBP1^{TB3E3} behave in a very similar manner in SPR, as they do in the plate assay (Figure 1C). B) The Scatchard plots of the more weakly binding LTBP1 constructs show more linear behaviour. K_d estimates are shown although saturation was not reached in these experiments.

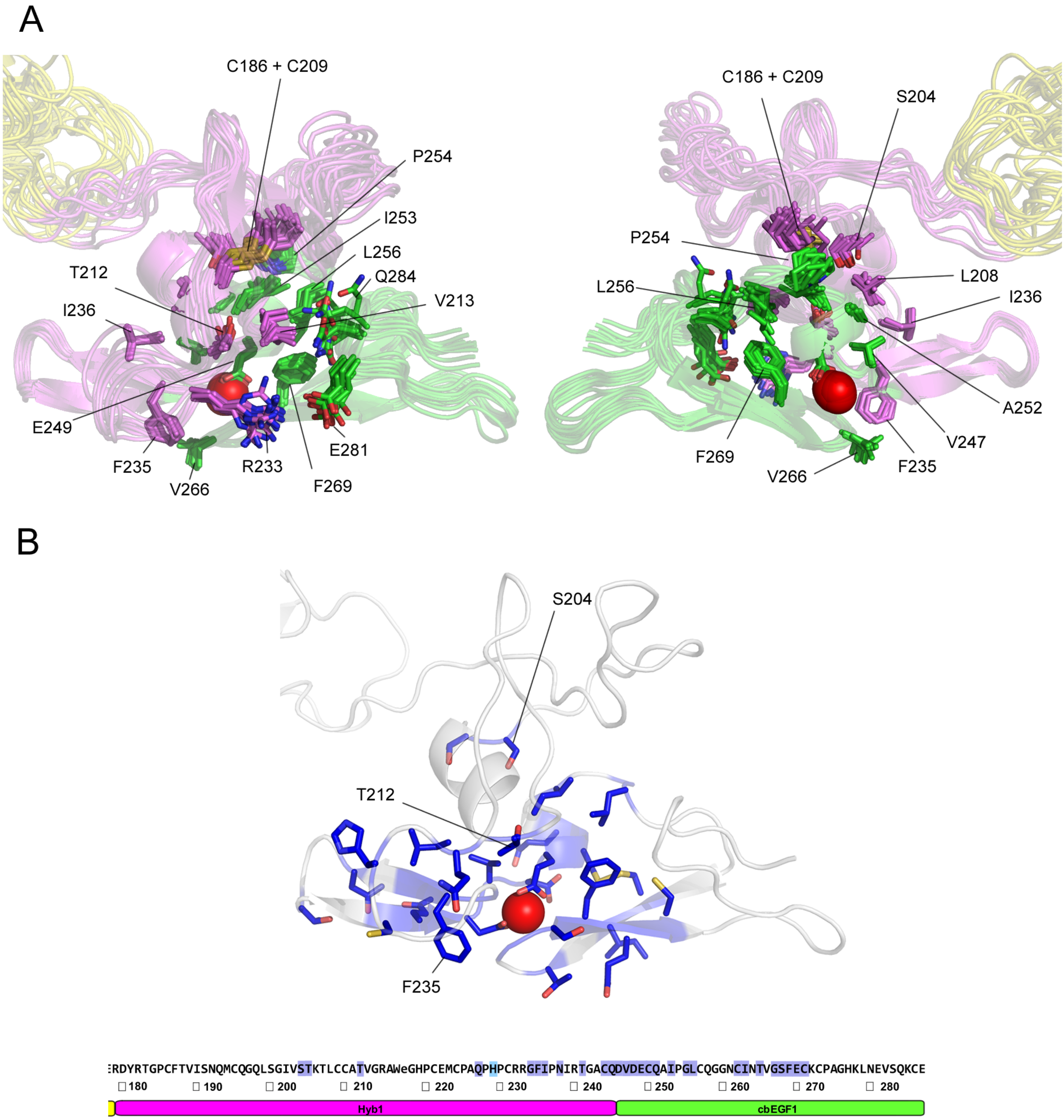

Figure S3. Related to Figure 6 and Table 1. Structure of the Hyb1-cbEGF1 inter-domain interface

(A) Two views of the interface between Hyb1 and cbEGF1 is defined by 74 unambiguous NOE restraints. A number of hydrophobic side chains define an extensive interface between Hyb1 (pink) and cbEGF1 (green), with interface side chains represented here as sticks. This interface surrounds the Ca^{2+}-binding site (red sphere) and may contribute to its high affinity. In many structures there is also a putative salt bridge between R233 in Hyb1 and E281 in cbEGF1. For clarity only 12 of the 20 structures in the ensemble are shown. (B) Residues in FBN1^{E2cbEGF1} with Ca^{2+}-sensitive NMR peaks are highlighted blue in the FBN1^{E2cbEGF1} sequence, and represented as blue sticks in the consensus structure.

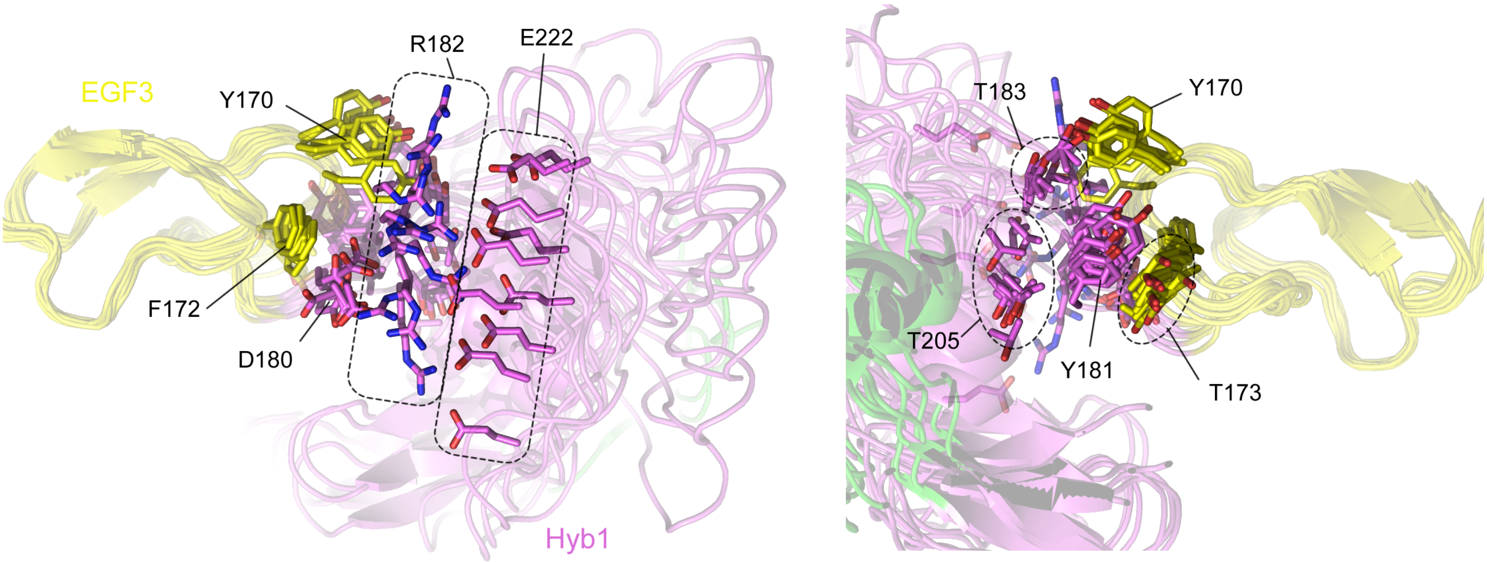

Figure S4. Related to Figure 6 and Table 1. Structure of the EGF3-Hyb1 inter-domain interface
Two views of the EGF3-Hyb1 inter-domain interface. Only a small number of residues define the EGF3-Hyb1 interface and this introduces some ambiguity into the relative orientation of EGF3 and Hyb1. However, the observed NOEs do define hydrophobic interactions between the aromatic side chains of Y170, F172, and Y181, and methyl groups of T173, T183 and T205. Furthermore in many structures R182 and E222 are close enough together that they could potentially form a salt bridge that could stabilise the inter-domain interface, although this salt bridge could still support a number of different domain orientations. The structures have been superimposed on EGF3 and the spread of orientations for Hyb1 can be seen. For clarity only 10 of the 20 structure ensemble are shown here.

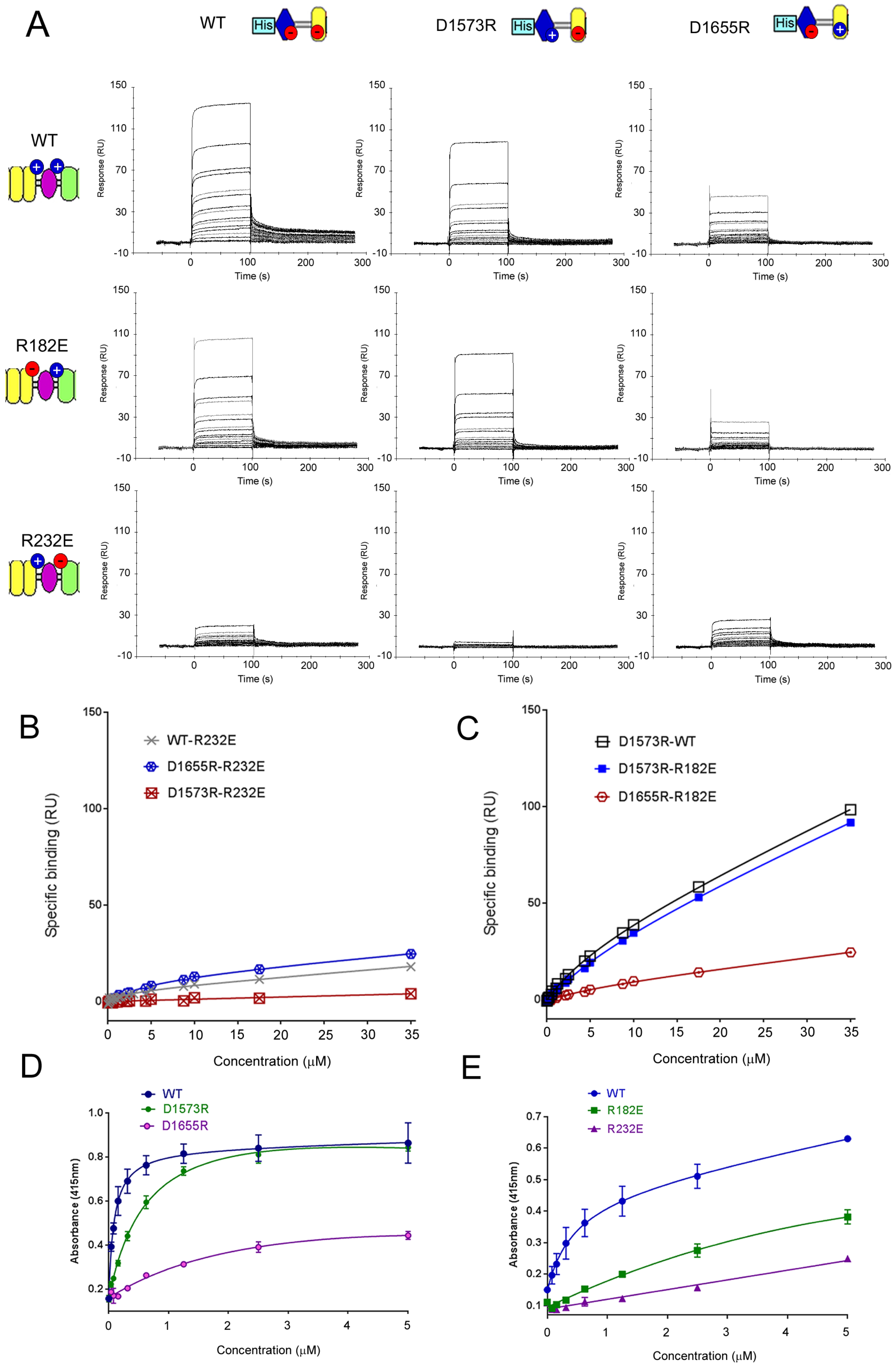

A
WT
D1573R
D1655R
His
WT
R182E
R232E
Response (RU)
Time (s)
B
WT-R232E
D1655R-R232E
D1573R-R232E
Specific binding (RU)
Concentration (μM)
C
D1573R-WT
D1573R-R182E
D1655R-R182E
Specific binding (RU)
Concentration (μM)
D
WT
D1573R
D1655R
Absorbance (415nm)
Concentration (μM)
E
WT
R182E
R232E
Absorbance (415nm)
Concentration (μM)

Figure S5. Related to Figure 7. SPR and Plate-based assays of LTBP1 and FBN1 charge reversal substitutions

(A) Blank subtracted multi-cycle SPR data with WT and charge-reversal FBN1^{E2cbEGF1} fragments amine-coupled to the chip. LTBP1^{TB3E3} fragments were injected as analytes at concentrations of 35, 17.5, 10, 8.75, 5, 4.38, 2.5, 2.18, 1.25, 1.09, 0.625, 0.547, 0.273, 0.137, 0.068, and 0.034 µM. (B) Plot of SPR responses from (A) for WT, D1655R LTBP1^{TB3E3} and D1573R LTBP1^{TB3E3} constructs binding FBN1^{E2cbEGF1} with the R232E substitution. (C) Plot of SPR responses from (A) for D1573R and D1655R LTBP1^{TB3E3} constructs binding WT and R182E FBN1^{E2cbEGF1}. The results in (B) show that the combination of D1655R LTBP1 and R232E FBN1 substitutions, which are residues involved in a putative salt bridge, gives no significant additional loss in binding compared to the single R232E substitution. This is consistent with these residues interacting with each other and not other residues in the FBN1/LTBP1 binding site; if these substitutions acted independently then an additive effect further reducing binding would be expected. The same is observed in (C) for the D1573R and R182E substitutions. In contrast to this, in (B) the combination of D1573R LTBP1 and R232E FBN1 substitutions, which are residues involved in two different putative salt bridges, gives an additional loss in binding compared to the single R232E substitution. This is consistent with these residues acting independently in two salt bridges giving an additive effect that further reduces binding. The same is observed in (C) for the D1655R and R182E substitutions. (D) Plate-binding assay data collected from a FBN1^{E2cbEGF1} coated plate with two LTBP1^{TB3E3} substitutions of interest, D1573R (green) and D1655R (magenta), compared to WT (blue). (E) Plate-binding assay data collected from wells coated with either WT Fbn^{E2cbEGF1}, R182E Fbn^{E2cbEGF1} or R232E Fbn^{E2cbEGF1}. The data presented for each protein in (D) and (E) are from a single plate; three repeats of each protein concentration were carried out to determine experimental error (standard deviation). The plate-binding assay data in (D) and (E) confirm the SPR results shown in Figure 7 D and E.

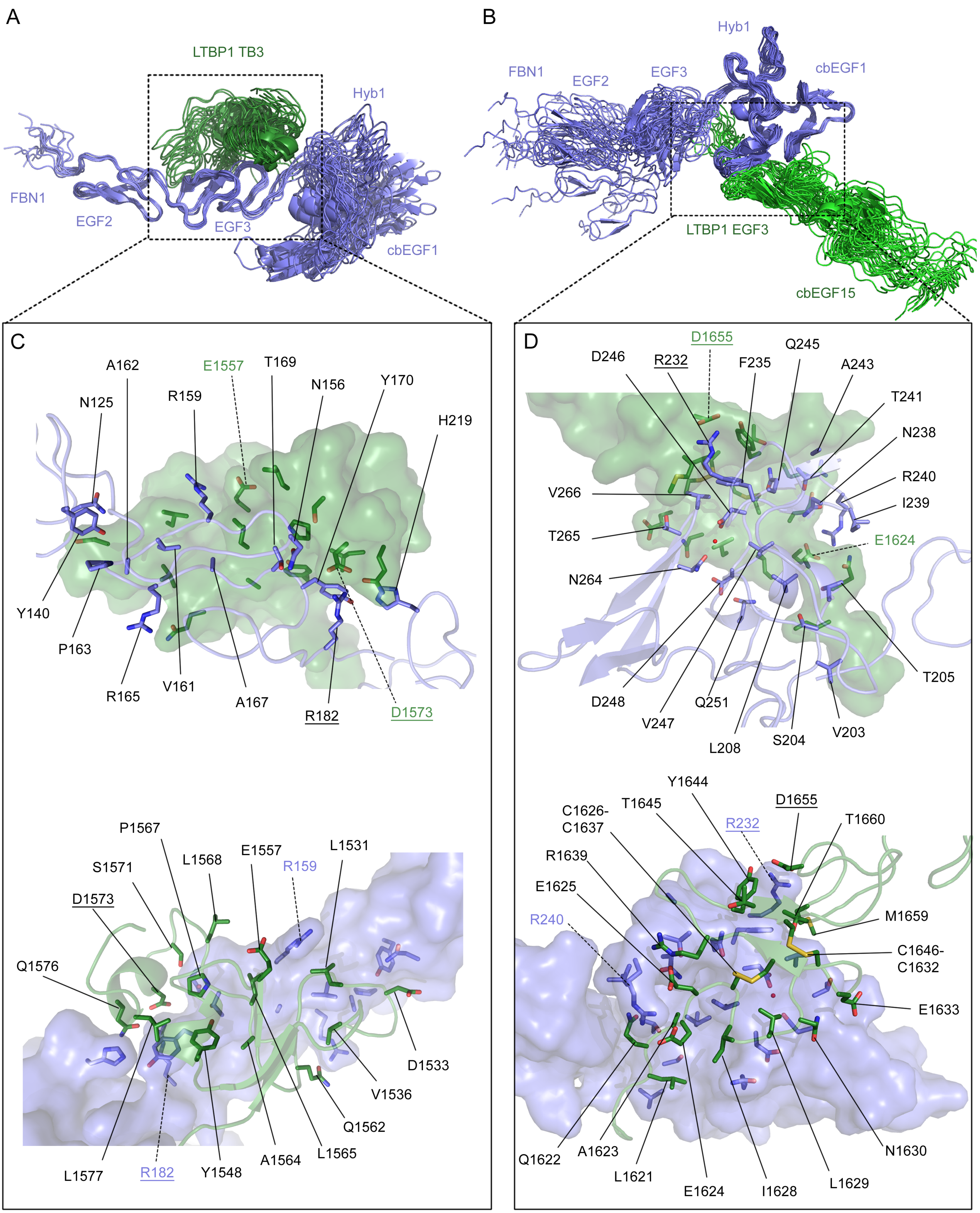

Figure S6. Related to Figure 8. Details of LTBP1-FBN1 interfaces identified by HADDOCK
(A) The top three best scoring HADDOCK clusters for the interaction of LTBP1^{TB3} and FBN1^{E2cbEGF1}

aligned by the EGF2-EGF3 domain pair of FBN1. (B) The top four best scoring HADDOCK clusters for the interaction of $LTBP1^{E3cbEGF15}$ with $FBN1^{E2cbEGF1}$ aligned by the Hyb1-cbEGF1 domain pair of FBN1. LTBP1 structures are coloured green and FBN1 coloured slate blue. (C) Residue side chains that form potential contacts between FBN1 EGF2-EGF3-Hyb1 and LTBP1 TB3. At the top FBN1 residues are highlighted and the surface of LTBP1 TB3 is shown in green, while on the bottom LTBP1 residues are highlighted and the surface of FBN1 is shown in slate blue. (D) Residue side chains that form potential contacts between FBN1 Hyb1-cbEGF1 and LTBP1 EGF3. On the top FBN1 residues are highlighted and the surface of LTBP1 EGF3 is shown in green, while on the bottom LTBP1 residues are highlighted and the surface of FBN1 is shown in slate blue. Residues identified as involved in salt bridges are underlined.

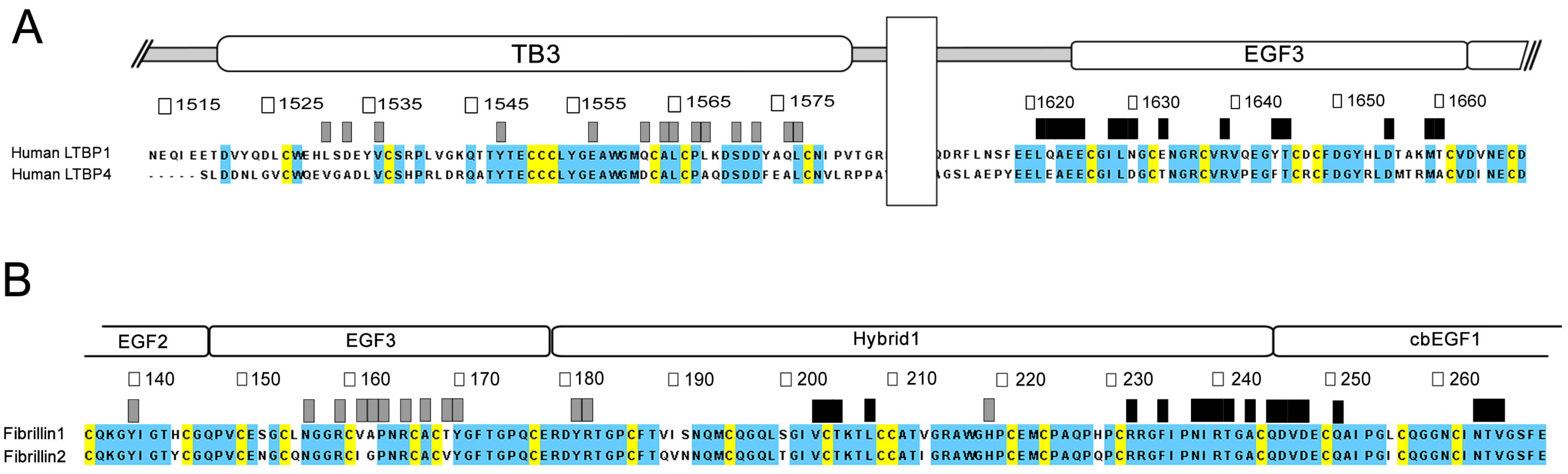

Figure S7. Related to Figure 8– Alignments of FBN and LTBP

(A) C-terminal region of LTBP1 implicated in FBN1 binding aligned with the C-terminal region of LTBP4. Grey boxes above the alignment indicate residues highlighted in Figure S6C as interacting with FBN1 EGF2-3 and Hyb1, while black boxes indicate residues highlighted in Figure S6D as interacting with FBN1 Hyb1-cbEGF1. Residues that are present in both sequences are highlighted blue, with the exception of cysteines which are highlighted yellow. A white box is shown where the inter-domain linker has been truncated. Comparison of the sequences of human LTBP1 and LTBP4 shows amino acid substitutions in both binding sites. The stronger FBN1 Hyb1-cbEGF1/LTBP1 EGF3 interface has substitutions Q1622E, N1630D, E1633T and T1660A while the weaker FBN1 EGF3-Hyb1/LTBP1 TB3 interface was substitutions D1533A, Q1562D, L1568A and Q1576A. These substitutions alter the electrostatic properties of the binding sites in the LTBPs and could substantially change the mode of interaction with FBN1. (B) N-terminal region of FBN1 implicated in LTBP1 binding aligned with the N-terminal region of FBN2. Grey boxes above the alignment indicate residues highlighted in Figure S6C as interacting with LTBP1 TB3, while black boxes indicate residues highlighted in Figure S6D as interacting with LTBP1 EGF3. Residues that are present in both sequences are highlighted blue, with the exception of cysteines which are highlighted yellow. Comparison of the sequences of human FBN1 and FBN2 shows complete conservation of residues involved in the strong FBN Hyb1-cbEGF1/LTBP1 EGF3 interface whereas three substitutions (V161I, A162G and T169V) are observed in the weaker FBN EGF3-Hyb1/LTBP1 TB3 interface. A reduction of binding affinity in the latter site could explain the observed differences in binding for FBN1/FBN2. N164, associated with dominant ectopia lentis via the N164S substitution (Comeglio et al., 2002), is conserved in FBN1/FBN2.

Supplemental Table S1. Related to Figure 8. – Restraints for final round of HADDOCK docking

	$LTBP1^{TB3}$ - $FBN1^{E2cbEGF1}$ docking	$LTBP1^{E3cbEGF15}$ – $FBN1^{E2cbEGF1}$ docking
FBN1 active residues	159,162,167,168,170,177,182	204,227,232,235,239,241,242,243,244,245,246, 249
FBN1 passive residues	125,126,128,138,140,145,147,148,149, 154,156,157,158,160,161,163,164,165, 166,169,171,172,173,174,175,176,178, 179,180,181,183,184,185	203,205,206,207,208,210,211,212,217,224,225, 226,228,229,230,231,233,234,236,237,238,240, 247,248,251,252,253,254,256,264,266,267,269
FBN1 fully flexible regions	111-118, 178-185, 236-243	111-118, 178-185, 236-243
LTBP1 active residues	1523,1562,1563,1569,1571,1574,1577, 1580	1622,1623,1625,1626,1628,1630,1631,1632, 1636,1638,1639,1641,1642,1643,1644,1646, 1647,1648,1656,1655
LTBP1 passive residues	1529,1539,1548,1549,1550,1561,1570, 1524,1525,1526,1528,1531,1532,1533, 1534,1535,1536,1537,1546,1547,1553, 1554,1559,1560,1564,1565,1566,1567, 1568,1572,1573,1575,1576,1578,1579, 1581,1582	1627,1628,1633,1634,1635,1637,1640,1644, 1645,1649,1650,1651,1652,1653,1654,1657, 1658,1659,1660,1661,1662,1620,1621,1622, 1623,1624,1625,1626,1630,1631,1632,1636, 1638,1639,1641,1642,1643,1646,1647,1648, 1656
LTBP1 fully flexible residues	1521- 1527, 1579-1582	1617- 1625,1638-1645, 1653-1660
Other restraints	ASSIGN (RESID 182 and NAME CZ and SEGID A) (RESID 1573 and NAME CG and SEGID C) 0.0 0.0 6.0	ASSIGN (RESID 232 and NAME CZ and SEGID A) (RESID 1655 and NAME CG and SEGID B) 0.0 0.0 6.0
HADDOCK results for highest scoring clusters		
HADDOCK score	-102.1 +/- 5.8	-131.1 +/- 15.5
Cluster size	15	7
RMSD from the overall lowest-energy structure	5.1 +/- 0.3	3.6 +/- 2.2
Van der Waals energy	-56.3 +/- 3.9	-81.6 +/- 4.9
Electrostatic energy	-162.3 +/- 16.6	-220.3 +/- 52.5
Desolvation energy	-28.5 +/- 4.2	-26.9 +/- 3.9
Restraints violation energy	152.5 +/- 22.24	214.6 +/- 12.64
Buried Surface Area	1707.7 +/- 55.4	2221.3 +/- 131.6
Z-Score	-1.3	-1.5

Supplemental Table S2. Related to Figure 7. – Primers for site-directed mutagenesis

Construct	Primer 5’ > 3’
**LTBP1:**	
$LTBP1^{TB3E3}$ D1521K fwd	CAAATAGAAGAAACTAAGGTCTACCAAGATTTGTGCTGGGAAC
$LTBP1^{TB3E3}$ D1521K rev	GTTCCCAGCACAAATCTTGGTAGACCTTAGTTTCTTCTATTTG
$LTBP1^{TB3E3}$ D1573R fwd	CCCTGAAGGATTCAGATCGCTATGCTCAGCTGTGTAAC
$LTBP1^{TB3E3}$ D1573R rev	GTTACACAGCTGAGCATAGCGATCTGAATCCTTCAGGG
$LTBP1^{TB3E3}$ E1625R fwd	GTTACAGGCTGAGCGATGCGGCATCCTCAATGG
$LTBP1^{TB3E3}$ E1625R rev	CCATTGAGGATGCCGCATCGCTCAGCCTGTAAC
$LTBP1^{TB3E3}$ E1642R fwd	CTGTGTGAGGGTCCAGCGAGGTTACACCTGCG
$LTBP1^{TB3E3}$ E1642R rev	CGCAGGTGTAACCTCGCTGGACCCTCACACAG
$LTBP1^{TB3E3}$ D1655R fwd	GGGTATCACTTGCGTACGGCCAAGATGACCTGTG
$LTBP1^{TB3E3}$ D1655R rev	CACAGGTCATCTTGGCCGTACGCAAGTGATACCC
**Fibrillin1**	
$FBN1^{E2cbEGF1}$ K138D fwd	CACTGTCTATGCCAGGATGGATACATAGGGACTCACTG
$FBN1^{E2cbEGF1}$ K138D rev	CAGTGAGTCCCTATGTATCCATCCTGGCATAGACAGTG
$FBN1^{E2cbEGF1}$ R182E fwd	CCAGTGTGAAAGAGATTACGAGACAGGCCCATGTTTTAC
$FBN1^{E2cbEGF1}$ R182E rev	GTAAAACATGGGCCTGTCTCGTAATCTCTTTCACACTGG
$FBN1^{E2cbEGF1}$ R232E fwd	CCAGCCTCACCCCTGCGAACGTGGCTTCATTCC
$FBN1^{E2cbEGF1}$ R232E rev	GGAATGAAGCCACGTTCGCAGGGGTGAGGCTGG
$FBN1^{E2cbEGF1}$ C204S fwd	CAACTCAGCGGGATTGTCTCCACAAAAACGCTCTGCTGTG
$FBN1^{E2cbEGF1}$ C204S rev	CACAGCAGAGCGTTTTTGTGGAGACAATCCCGCTGAGTT
